# Supplementary material for: Vector control strategies in Brazil: a qualitative investigation into community knowledge, attitudes and perceptions following the 2015–2016 Zika virus epidemic
Source: BMJ Open. 2022 Jan 27;12(1):e050991. doi: 10.1136/bmjopen-2021-050991 (PMC8808399; doi:10.1136/bmjopen-2021-050991)
Supplement: Supplementary data [file bmjopen-2021-050991supp001.pdf]

Supplementary File 1: Topic Guide

Bancroft *et al.* Vector control strategies in Brazil: A qualitative investigation into community knowledge, attitudes and perceptions following the 2015–16 Zika virus epidemic. *BMJ Open* 2021 [Manuscript ID: bmjopen-2021-050991]

SOCIODEMOGRAPHIC SURVEY:

Socio-demographic data collected during recruitment.
Age: 18–30 [ ]   31–49 [ ]   **Socioeconomic status:** High [ ]   Middle [ ]   Low [ ]

Date of interview	
Location of interview	
Name of interviewer	
Name of observer/note taker	
Time interview started	
Time interview ended	

INTERVIEW OPEN

- **Introduction by interviewer to the study**
- **Review and signing of informed consent form**
- **Start recording**

[12 questions]

1. **Mosquito control by families and the community.**

   a. What do you do in your home to reduce the number of mosquitoes that exist in your region and the number of bites that you and your family receive?
   Prompts: environmental cleaning, repellents, long sleeves, screens, bed nets etc.
   b. Is there any kind of community effort to reduce mosquito outbreaks?
   Detail (investigate this aspect well).

2. **Mosquito control by local authorities**
   a. What mosquito control activities, if any, are undertaken by the local authorities in your community?

3. **Changes in mosquito control practices**
   Has there been a change in mosquito control practices in your community, and in your own personal protection, since the emergence of Zika? If so, please provide details.

4. **Preferred mosquito control activities**
   Zika mosquitoes bite during the day. Given that, what kind of mosquito control would you <u>like</u> to see? *Better detail on personal protection including clothing.*

5. **Personal protection interventions**
   a. What do you think of personal protection interventions / alternatives / practices such as mosquito repellent clothing?
   b. How likely are you to use these alternatives and what would be your considerations?
   Prompt for cost, safety, comfort, fashion, duration of effectiveness.

6. **Concern about mosquito-borne diseases**
    a. In relation to various issues that you and your family have to manage on a daily basis, how much are you concerned about diseases transmitted by mosquitoes, such as dengue, chikungunya, yellow fever and Zika?
    b. Are these four diseases of equal concern to you, or is one of more concern than the other three? *Detail.*

7. **Knowledge about Zika**
    a. Turning specifically to Zika, do you know anyone personally who has had Zika?
    b. If so, what is your relationship with this person / people?
    c. What do you know about Zika?
    d. Are there any aspects of the disease you would like to know more about?

8. **Sources of knowledge about Zika**
    a. Where did you receive your knowledge about Zika? *(Prompt to include social media)*
    b. Which of the Zika information sources do you think was the best, and which have been the least useful?

9. **Messages from Zika**
    a. What are the main messages about Zika that you received from the authorities?
    Poll for mosquito control, bite reduction and pregnancy issues.
    b. Were these messages useful for you, or not? Explain.
    Prompt for understanding, action, relevance, communication channel and key messages.

10. **Postponement of pregnancy**
    a. Do you know women in your community who wanted to postpone pregnancy as a means of avoiding a baby with microcephaly?
    b. Has this issue been a matter of concern or discussion in your community?

11. **Sexual transmission of Zika**
    a. The Zika virus can be sexually transmitted to women by infected men. Do you think that the men in your community would be willing to practice safe sex in the recommended six months if their partner was pregnant, and they knew they were infected with Zika?
    Prompt for condom use, sex without penetration etc.
    b. Can you think of any messages that could be used to encourage men recently infected with Zika to practice safer sex? [MEN ONLY]
    c. The Zika virus can be sexually transmitted to women by infected men. Would you be willing to use a condom for the recommended six months if your partner was pregnant and knew you were infected with Zika?

12. **Abortion**
    a. Are you aware of cases in your community of pregnant women who have sought abortions because they feared they were carrying a baby with microcephaly?
    b. If so, what did people say about it?
    c. Do people in your community agree that a woman should have the right to terminate the pregnancy in these circumstances, or not? Or do they think she should carry the baby to term even if the baby may have microcephaly?

CLOSURE

- **Provide an opportunity for participants to discuss and ask questions about anything about Zika that they are in doubt about.**

- **Provide an official Zika information leaflet from the Ministry of Health website.**

- **Final question:** ***Would you be willing to attend a meeting to discuss the results of our study in 2 or 3 months? If so, please provide us with your contact details so that we can contact you.***

- **Thank all participants for their involvement and valuable responses.**
