# Supplementary material for: Vector control strategies in Brazil: a qualitative investigation into community knowledge, attitudes and perceptions following the 2015–2016 Zika virus epidemic
Source: BMJ Open. 2022 Jan 27;12(1):e050991. doi: 10.1136/bmjopen-2021-050991 (PMC8808399; doi:10.1136/bmjopen-2021-050991)
Supplement: Supplementary data [file bmjopen-2021-050991supp002.pdf]

Supplementary File 2: Consolidated criteria for reporting qualitative research (COREQ): a 32-item checklist [1]

Bancroft *et al.* Vector control strategies in Brazil: A qualitative investigation into community knowledge, attitudes and perceptions following the 2015–16 Zika virus epidemic. *BMJ Open* 2021 [Manuscript ID: bmjopen-2021-050991]

**Domain 1:** Research team and reflexivity		Response		Referenced
**1.**	**Interviewer/ facilitator**<br>Which author/s conducted the interview or focus group?	Yes	**Salvador lead:** Jorge Iriart<br>Facilitators: Vera Lucia Zaher-Rutherford, Tania Boccia, Mônica Manir.<br>**Jundiaí lead:** Eduardo Massad (Principal investigator)<br>Facilitators: Ana Maria Rico, Greice Bezerra Viana, Fernanda Macedo da Silva Lima.	p.6 (123-125)<br>p.20 (573-575)<br>p.20 (578)
**2.**	**Credentials**<br>What were the researcher's credentials?	Yes	**Grace Power:** Project Manager at the Global Vector Hub, London School of Hygiene & Tropical Medicine (LSHTM), UK.<br>**Dani Bancroft:** MSc student, Department of Public Health, Environments and Society, Faculty of Public Health and Policy, LSHTM, UK.<br>**Robert Jones:** Research Fellow in Department of Disease Control, Faculty of Infectious and Tropical Diseases, LSHTM, UK.<br>**Jorge Iriat:** Associate Professor, Institute of Collective Health (ISC), Federal University of Bahia, Brazil.<br>**Eduardo Massad:** Professor, School of Medicine, University of São Paulo and Fundação Getulio Vargas, Brazil.<br>**Raman Preet:** Research Coordinator, Department of Epidemiology and Global Health, Faculty of Medicine, Umeå University, Sweden.<br>**John Kinsman:** Associate Professor, Department of Epidemiology and Global Health, Faculty of Medicine, Umeå University, Sweden.<br>**James Logan:** Head of Department of Disease Control, Faculty of Infectious and Tropical Diseases, LSHTM, UK.<br>**Interview facilitators:** local MDs, nurses, psychologists and sociologists.	All authors:<br>p.20 (578-582)<br><br>Facilitators:<br>p.6 (111-112)<br>p.20 (573-575)
**3.**	**Occupation**<br>What was their occupation at the time of the study?	Yes		

**4.**	**Gender**<br>Was the researcher male or female?	Yes	Both Salvador and Jundiaí interview teams consisted of one male coordinator and three female interview facilitators.	p.20 (573-575)
**5.**	**Experience and Training**<br>What experience or training did the researcher have?	Yes	The principal investigators in Salvador and Jundiaí are native Brazilian Portuguese speakers familiar with the local context of Zika virus in Bahia and São Paulo. The ZikaPLAN team carried out training and pilot testing of instrument with LSHTM visiting researchers. This was designed following 17 in-depth interviews with health professionals, including Salvador health professionals working in a Primary Care Unit and in private clinics, and community leaders, with three religious leaders from Kardecism, Candomblé (an Afro-Brazilian religion) and an evangelical Christian church.	p.6 (123-128)<br>p.20 (578-582)
**6.**	**Relationship**<br>Was a relationship established prior to study commencement?	No	No prior relationship was established.	N/A
**7.**	**Participant knowledge**<br>What did the participants know about the researcher? e.g. personal goals, reasons for doing the research	Yes	There were no direct benefits to participating in the study. Participants were provided information on the study objectives and relevance of the research, and a leaflet on Zika virus published by the Brazilian Ministry of Health at the end of the study.	N/A
**8.**	**Interviewer**<br>What characteristics were reported about the interviewer/facilitator?	Yes	The principal investigators in Salvador and Jundiaí are native Brazilian Portuguese speakers familiar with the local context of Zika virus in Bahia and São Paulo states. Interview facilitators were also local to the study sites.	p.6 (111-112)<br>p.6 (123-125)

**Domain 2:** Study design		Response		Referenced
**9.**	**Methodological orientation and Theory**<br>What methodological orientation was stated to underpin the study? e.g. content analysis.	Yes	Thematic analysis guided by Braun and Clarke (2006).[2]	p.6 (134)
**10.**	**Sampling**<br>How were participants selected?<br>e.g. purposive, convenience, consecutive.	Yes	Purposive sample for women of reproductive age (18–49). Not all men recruited into the study were the intimate partners of female participants.	p.6 (115-120)

**11.**	**Method of approach**<br>How were participants approached?<br>e.g. face-to-face, telephone, email.	No	Face-to-face recruitment at outpatient clinics, NGO settings and through researcher networks in the community.	p.6 (115-120)<br>p.6 (125-128)
**12.**	**Sample size**<br>How many participants were in the study?	Yes	A total of 120 participants: 103 women in focus groups (60 in Jundiaí and 43 in Salvador) and 17 men in semi-structured interviews (9 in Jundiaí and 8 in Salvador).	p.2 (18-19)<br>p.7 (141-145)<br>p.7 (147)
**13.**	**Non-participation**<br>How many people refused to participate or dropped out? Reasons?	No	The original study protocol proposed 6–8 women per focus group. Salvador groups ranged from 4–7. For Jundiaí, the size of number of participants in each focus group was not provided for data analysis.	p.7 (141-145)<br>[Table 1, p.7]
**14.**	**Setting of data collection**<br>Where was the data collected?<br>e.g. home, clinic, workplace	Yes	In Salvador, interviews were conducted in outpatient rooms at Primary Care Clinics (FGDs) and at private residences (for men). FGDs in Jundiaí were conducted at an NGO-run community centre and in University Hospital faculty buildings.	p.6 (117-120)
**15.**	**Presence of non-participants**<br>Was anyone else present besides the participants and researchers?	Yes	Visiting LSHTM researchers observed the interviews. No non-ZikaPLAN staff were present for the interviews.	p.6 (124-125)<br>p.20 (573-575)<br>p.20 (578)
**16.**	**Description of sample**<br>What are the important characteristics of the sample? e.g. demographic data, date	Yes	Interviews took place between March and August 2017. Sociodemographic data was not collected during all interviews; stratified age groups were provided for the majority of female FGDs but not for male participants.	p.6 (106-108)<br>p.6 (115-117)<br>p.7 (143-145)<br>p.18 (526-528)
**17.**	**Interview guide**<br>Were questions, prompts, guides provided by the authors? Was it pilot tested?	Yes	The topic guide, which includes questions, prompts and the sociodemographic data collected is provided in Supplementary File 1. This was pilot tested during training of interview facilitators with LSHTM research team present.	p.6 (108-111)<br>p.6 (124-125)<br>[Supplementary file 1]
**18.**	**Repeat interviews**<br>Were repeat interviews carried out? If yes, how many?	No	No follow up interviews were carried out, although all interview participants were invited to attend a follow-up session in September 2017 for dissemination of initial findings.	p.6 (128-130)<br>[Supplementary file 1]
**19.**	**Audiovisual**<br>Did the research use audio or visual recording to collect the data?	Yes	The source data was audio recordings that were transcribed into Brazilian Portuguese by the Brazil ZikaPLAN team. This was then translated into English, with excerpts of transcripts verified for accuracy and credibility by the University College London Digital Media translation service. The source data was not shared for data analysis.	p.6 (111-112)<br>p.18 (523-526)<br>p.20 (578-580)

**20.**	**Field notes**<br>Were field notes made during and/or after the interview or focus group?	Yes	ZikaPLAN observers and facilitators took field notes during the sessions.	N/A
**21.**	**Duration**<br>What was the duration of the interviews or focus group?	Yes	Each interview was arranged to last 60–90 minutes. Timestamps for interviews were not shared for analysis, but the wordcount of each transcript was presented in Table 1.	p.7 (142-145)<br>[Table 1, p.7]
**22.**	**Data saturation**<br>Was data saturation discussed?	Yes	Yes, regarding participant responses to question 5 in the topic guide on novel repellents for personal protection.	p.19 (539-541)<br>[Supplementary file 1].
**23.**	**Member checking**<br>Were transcripts returned to participants for comment and/or correction?	No	No, although all interview participants were invited to attend a follow-up session in September 2017 for dissemination of initial findings.	p.6 (128-130)<br>[Supplementary file 1]

**Domain 3:** Analysis and findings		Response		Referenced
**24.**	**Coders**<br>How many data coders coded the data?	Yes	One researcher for initial coding and three authors of one full FGD transcript. The principal investigators in Brazil carried out an initial analysis of transcripts following data collection. The data was then passed on to LSHTM for independent data analysis. The initial coding framework was presented to the principal investigators in Brazil for confirmability and triangulation purposes prior to theme generation.	p.6 (128-130)<br>p.6 (134-136)<br>p.18 (523-526)
**25**	**Coding tree**<br>Did authors provide a description of the coding tree?	Yes	The full codebook is provided in Supplementary File 3. A summary table of the key and major themes and a concept map of minor themes are provided in the manuscript.	p.7 (152-154)<br>[Table 2, p.8]<br>[Supplementary file 3]
**26**	**Derivation of themes**<br>Were themes identified in advance or derived from the data?	Yes	Coding was derived from the data. Theme generation was mostly inductive, with some deductive elements from grouping of codes together as responses to a certain question in the topic guide. Major themes were later mapped against constructs in a pre-defined conceptual framework for behaviour change for a potential fit (Rosenstock's Health Belief Model).	p.5 (89-92)<br>p.6 (133-138)<br>p.7 (148-154)<br>[Figure 1; Figure 2]<br>p.18 (528-532)
**27.**	**Software**<br>What software, if applicable, was used to manage the data?	Yes	Microsoft Excel was used to record sociodemographic data for each interview and observations, as well as administrative data, such as wordcount, date and file names for the Brazilian and English transcripts as an audit trail. NVivo 12 (QSR International, 2012) was used for coding and mapping Figure 2. Figure 2 was later redesigned in Lucidchart (Lucid Software Inc., 2021).	p.6 (133-134)<br>[Figure 1; Figure 2]

**28.**	**Participant checking**<br>Did participants provide feedback on the findings?	No	At the end of each interview participants were invited to consent for their contact information to be collected to disseminate the research findings. However, participant checking was not possible for this study.	p.6 (128-130)<br>[Supplementary file 1]
**29.**	**Quotations presented**<br>Were participant quotations presented to illustrate the findings? Was each quotation identified?	Yes	Quotations in the manuscript were identified by focus group or interview site and number (unit of analysis), with the corresponding age group (18–30 or 31–49) in Table 1.	[Results section]<br>p/6 (106-107)<br>p.7 (141-145)<br>[Table 1, p.7]
**30.**	**Consistency**<br>Was there consistency between the data presented and the findings?	Yes	–	p.18 (528-533)<br>p.19 (559-560)
**31.**	**Clarity of major themes**<br>Were major themes clearly presented in the findings?	Yes	A concept maps for themes was produced and this was used to navigate description of findings in relation to one another.	p.6 (137-138)<br>p.7 (152-154)<br>[Figure 2; Table 2, p.8]
**32.**	**Clarity of minor themes**<br>Is there a description of diverse cases or discussion of minor themes?	Yes	Key and major themes are defined in Table 2 in the manuscript, and minor themes described in the findings. All themes are defined fully in the codebook (Supplementary File 3).	p.7 (148-154)<br>[Table 2, p.8]<br>[Results section]<br>[Supplementary file 3]

1 Tong A, Sainsbury P, Craig J. Consolidated criteria for reporting qualitative research (COREQ): a 32-item checklist for interviews and focus groups. *International Journal for Quality in Health Care* 2007;19(6):349–357. doi:10.1093/intqhc/mzm042

2 Braun, V, Clarke V. Using thematic analysis in psychology. *Qual Res Psychol* 2006;3:77–101. doi:10.1191/1478088706qp063oa
