# Supplementary material for: Vector control strategies in Brazil: a qualitative investigation into community knowledge, attitudes and perceptions following the 2015–2016 Zika virus epidemic
Source: BMJ Open. 2022 Jan 27;12(1):e050991. doi: 10.1136/bmjopen-2021-050991 (PMC8808399; doi:10.1136/bmjopen-2021-050991)
Supplement: Supplementary data [file bmjopen-2021-050991supp003.pdf]

Supplementary File 3: Final transcript coding framework (Codebook)

Bancroft *et al.* Vector control strategies in Brazil: A qualitative investigation into community knowledge, attitudes and perceptions following the 2015–16 Zika virus epidemic. *BMJ Open* 2021 [Manuscript ID: bmjopen-2021-050991]

**1. KNOWLEDGE**	Knowledge of MBD and ZIKV at the time of the study, and cues to action which are recalled stimuli for a decision-making process that may lead to behaviour change.[32]
**1.1 Knowledge of MBDs**	Depth of understanding of ZIKV/MBDs, vector control and misinformation.
Key messages	Reponses to Question 9 in the topic guide: “What are the main messages about Zika that you received from the authorities?” (Poll for mosquito control, bite reduction and changes in behaviour for reproductive health).
MBD outbreaks	General knowledge on other mosquito-borne diseases: yellow fever, chikungunya, dengue fever. For example, references to outbreaks and epidemics, changes in prevalence/incidence, pathophysiology and vaccination campaigns.<br>Excluded: comments where ZIKV is the focus (coded as ‘ZIKV General’), unless being compared to other MBDs.
Misinformed	Comments made by participants that may indicate misinformation or uncertainty around key messages related to MBDs.
Sexual transmission	Knowledge related to sexual transmission of ZIKV of both the participant and others in their social circle.<br>Excluded: content of messaging related to sexual transmission (coded as ‘Key messages’).
ZIKV (General)	Other knowledge related to ZIKV that does not fall into codes sexual transmission, severity of ZIKV symptoms, perceived risk (susceptibility), or experience of ZIKV (internal cues to action).
**1.2 External cues to action**	Stimuli from members of participants social network, the media, healthcare providers, the workplace or other community groups that trigger a decision-making process to seek additional information, engage in vector control or mosquito-bite reduction strategies, or other health seeking behaviours.
Health campaign	Alerts, visits from health agents for risk communication, billboards, posters and pamphlets, or messaging in the media explicitly described by the participant as being official public health information.
Zika Projects	The *Zika Project*, official NGO or volunteer projects taking place in hospitals (not always clear).<br>Excluded: activities identified as being conducted by local or national authorities (e.g. City Hall, Ministry of Health).
Healthcare	Accessing different forms of healthcare, such as maternity services, community clinics, dentists etc.<br>Excluded: experiences of having ZIKV or other MBDs, descriptions of symptoms of poor health (coded ‘Other poor health’).

Bancroft D, *et al. BMJ Open* 2022; 12:e050991. doi: 10.1136/bmjopen-2021-050991

Media	Parent code for references to media. Excluded: Official health campaign content (when clearly identified).
Broadcast media	Any media source that has been broadcast for entertainment purposes, such as television soaps and radio, or TV advertisements and print media, such as magazines and newspapers.
Online and social media	Casual or purposeful research online: accessing websites that may provide information about ZIKV.<br>Messages and advertisements through social media, such as WhatsApp, Facebook, Instagram etc.
Social circle	Friends, neighbours, family members. Excluded: co-workers or acquaintances in formal settings (e.g. university, volunteer groups)
Work or education	Parent code for references to formal settings. Excluded: volunteering positions (e.g. in hospitals or ZIKV projects).
Higher Education	Participant is a current or former university/college student where ZIKV messaging has been delivered as part of a formal curriculum. Or there have been opportunities to access lectures and seminars on the epidemic.
Schools	Recalling experience of formal education for participants (e.g. high school).<br>Or messages that children in the participants social circle have passed on to the participant informally.
Workplace	Participant either works in healthcare, formal education (teachers) or other profession where Zika messaging has been delivered at their workplace (e.g. works for the City Hall).
Community groups	Observing preventive activities or other stimuli in the community: informal groups (e.g. women's groups, gangs), community volunteer groups, gangs, centres of worship, neighbourhood associations, sports teams (e.g. capoeira, football) etc.
Government	National, state and municipal levels of government responsible for defining activities and protocols for *Aedes* interventions, including *"budget, personnel, technical guidelines, approved substances, routines, evaluation, and relationships with other sectors, such as education and public health"*.[4]
Local authorities	Aldermen, City Hall urban planning including waste management services. Health agents from the City Hall.<br>Excluded: 'health agents' described as being from an NGO, Ministry of Health or other national body.
National authorities	References to the national government: politicians, deployment of the army, legislation and policy makers, the Ministry of Health (e.g. official surveillance staff from the Brazilian MoPH) or other national bodies.
No action	No vector control strategies are recalled to have taken place in the community, except for examples of vector control activities that have taken place more than one year prior to the start of epidemic in 2015.

**1.2 Internal cues to action**	Personal or secondary experience of confirmed/suspected cases of MBDs provide a stimulus for a decision-making process that leads to health seeking behaviour.
Experience of other MBDs	Confirmed or suspected cases of non-ZIKV mosquito-borne arboviruses by the participant or in the participant's social network.
Experience of Zika	Confirmed or suspected cases of ZIKV infection of the participant or in the participants social network.
Other poor health	Discussion of poor health that might be: non-communicable (e.g. disability or chronic conditions); related to non-ZIKV pregnancy complications; infectious diseases such as measles and H1N1 viruses; and other vector borne diseases such as Leptospirosis, tick-borne diseases, Chaga's disease etc. Excluded: MBDs.

**3. ATTITUDES & NORMATIVE BELIEFS**	Personal attitudes are internal assessments of knowledge and cues to action for MBD preventive behaviours. Normative beliefs may inform personal attitudes according to how others perceive the behaviour in a social setting, such as the community.[1]
**3.1 Perceived Susceptibility**	A subjective assessment of risk of ZIKV infection or a CZS pregnancy. Combines with perceived severity for perceived threat.[2]
Mosquito population	Comments on the burden of the mosquito population in a specific geographical area, mosquito physiology and behaviour. Other observations made by the participant or members of the participants social circle on the activity of mosquitoes in that area.
Risk response	Perceived risk of ZIKV transmission and CZS. For example: the periodomicile does not have a large mosquito population; the participant is not pregnant or has undergone the menopause; perceptions that the risk of contracting ZIKV to be very low. (Also includes responses to question 5 of the topic guide).
**3.2 Perceived Severity**	A subjective assessment of the severity of ZIKV and potential consequences of infection or a CZS pregnancy. "The combination of perceived severity and perceived susceptibility is referred to as perceived threat".[2]
CZS severity	Experience of caring for a child with microcephaly in the in the participants social network. Perceptions of the severity of microcephaly in the community, e.g. the burden of care giving for a child with microcephaly (the financial or social implications). Excluded: comments around male support to care for a child with CZS.
ZIKV Severity	Perceptions related to the severity of symptoms of ZIKV. Comments about concern or even fear related to ZIKV. Excluded: comments about CZS caregiving.
Other MBD Severity	Perceptions related to the severity of symptoms of other MBDs. Comments about concern or fear related to other MBDs. Excluded: Perceptions of poor health due to non mosquito-borne arboviruses.

**3.3 Perceived Barriers**	An individual's assessment of the obstacles to ZIKV preventive behaviours, including condom use to prevent sexual transmission, mosquito bite-reduction and vector control strategies.
Abortion	Awareness of individuals in the community that have terminated a pregnancy due to ZIKV or has undergone an abortion themselves as a result of concern of giving carrying a microcephaly child. Also includes community perspectives on the acceptability of abortion. Excluded: rights to abort and legislation.
Abortion rights	Participant responses to Question 12 in the topic guide: "Do people in your community agree that a woman should have the right to terminate pregnancy in these circumstances? Or do you understand that she should carry the pregnancy through to the end even if the baby has microcephaly?"
Depends on circumstances	More consideration around abortion. Comments that it is both acceptable and unacceptable, with examples of scenarios where abortion may be necessary or comments such as 'it's difficult' or 'it's complicated'. Includes discussion of financial circumstances and male partner support to evidence reasoning (only in reference to abortion). Excluded: caring for a child with CZS.
Opposed to abortion	Explicit opposition to the rights to abort. May cite religious grounds and morality e.g. perceptions of foetal viability and human rights. Normative beliefs around responsibility of pregnant mothers and their male partners. Unspecified negative responses, or strong opposed even when prompted by thee interviewer about microcephaly.
Supports rights to abort	Explicit support for the right to choose abortion. May express the need for legislative change, or cite perceptions of women's rights and autonomy regarding reproductive health.
Unclear response to abortion	Conflicted, contradictory or unintelligible response. May indicate discomfort expressing personal attitudes that conflict with the majority position.
Repellent acceptance	Parent code for likelihood of community acceptance of novel repellents adoption (response to question 5 of the topic guide).
Appearance response	Aesthetic criteria related to the perception of wearing novel novel repellents in the community (e.g. smell, fashion).
Comfort response	Negative responses related to comfort of repellent clothing such as overheating, restricting physical movement and allergies or discomfort caused by repellent products.
Repellent effectiveness	Responses related to perceived effectiveness of novel repellents for mosquito bite reduction. Scepticism or expression of interest may be contingent on how effective novel repellents are in practice (response to question 5 of the topic guide).

Bancroft D, *et al. BMJ Open* 2022; 12:e050991. doi: 10.1136/bmjopen-2021-050991

Repellent accessibility	Parent code for perceptions of the ability to access novel novel repellents (response to question 5 of the topic guide).
Affordability response	Comments related to cost of novel novel repellents being a barrier to their adoption.
Availability response	Comments related to local availability of repellent tools for purchase, such references to vendor stock outs and likelihood of vendors in their community to sell novel repellent tools like clothing. Also included are comments around provision of novel repellents as gifts-in-kind from NGOs or the local or national authorities (e.g. through Bolsa Familia).
Awareness response	Participants awareness of novel repellent tools for personal protection. Comments about being unaware or vague.
Community cohesion	Social cohesion is defined as the *"extent of connectedness and solidarity among groups within society",*[3] such as support from the community for vector control or being able to seek social support when unwell. Comments about absent or poor relationships with neighbours, or not allowing unsolicited calls to household due to concerns about neighbourhood violence
Responsibility	Observation about participants expressing frustration over current preventive practices or ZIKV messaging, or being unable to negotiate shared responsibility for communal spaces for vector control. Blame of third parties or authorities.
Internal responsibility	Expressing perceived locus of control for behaviour change lies with individual.
External responsibility	Expressing that the perceived locus of control in relation to behaviour change around ZIKV and messaging as lying further upstream, such as with authorities (local, national).
Male support	Perceptions of male partners and the level of support participants feel they have from partners for ZIKV prevention. Perceptions of other male members of participants social circles, including family members, including normative beliefs related to gender (e.g. machísimo). Excluded: references to condom negotiation.
Negotiating condom use	Responses to question 11 of topic guide: "Do you think that the men in your community would be willing to practice safe sex (condom use, sex without penetration)? Do you think that if a man knew he was infected he would use a condom for six months?"

**3.4 Perceived benefits and self- efficacy**	"Perceived benefits refer to an individual's assessment of the value or efficacy of engaging in a health-promoting behaviour to decrease risk of disease."[2] Self-efficacy refers to an individual's perception of their competence to successfully undergo a behaviour change.[2]
Comfort	Positive perceptions of novel repellents use such as avoiding discomfort from bed nets, overheating from having to close windows and doors at night time, 'stickiness' or dislike of wearing topical repellents and allergic reactions (if referring to clothing).
Protection	Responses to question 5 of the topic guide related to enhanced protection of themselves or others in their social network from MBD infection. e.g. during pregnancy, family members such as children or the elderly.
Likelihood of adoption	Willingness or likelihood to adopt novel repellents. Describes being motivated or unmotivated to take responsibility for household level behaviours or community participation to reduce transmission of ZIKV. Excluded: change in behaviour that has happened.
Negative response	Unspecified negative response to Q5 of the topic guide indicating disinterest or not willing to adopt novel repellents.
Positive response	Unspecified positive response to question 5 of the topic guide indicating willingness or interest to adopt novel repellents.

**3. BEHAVIOUR CHANGE**	Behaviour changes attributed to the ZIKV epidemic, existing MBD preventive behaviours or no changes to mosquito population control or bite-reduction strategies, including use of novel repellent tools.
**3.1 Household Level**	Practices to prevent mosquito breeding sites, mosquito-bite reduction and mosquito entry to the household.
Mosquito bites	Preventive practices taken personally to reduce risk of mosquito bites.
Avoidance behaviour	Avoiding certain times of day or areas known to have more mosquitoes. Closing of windows or doors to prevent mosquito entry.
Bed nets, screens	Insecticide treated or untreated mosquito bed nets, window or door screens to prevent mosquito entry.
Electronic devices	Plug in mosquito repellent devices, air conditioning and fans, electric 'racket' killing devices, sonic devices.
Long clothing	Covering up with long sleeves or legs to prevent exposed skin to mosquitoes.

Other topical products	Applying moisturiser, sun screen or other topical lotions that are not manufactured to function as mosquito repellents.
Repellents	Chemical or citronella repellents, room sprays or alternative methods like burning coils, egg shells, cardboard etc. Excluded: electronic plug-in repellents or sonic devices.
Supplements	Participants describe taking oral supplements due to belief this will reduce likelihood of mosquito bites (e.g. vitamin B complex).
Mosquito population control	Parent code for preventive practices related to vector control in the household.
Animals	Wild dogs, pets or other non-arthropod animals. Coded for potential implications for One Health.
Garbage disposal	Further detail relating to garbage collection or recycling to prevent water accumulation.
Hygiene	Using soap, scrubbing surfaces, applying disinfectant, sweeping and references to hygiene and cleanliness.
Insecticide	Water treatments to stop larval growth cycle (larvicides), or spraying chemical insecticides indoors or around the periodomicile.
Stagnant water	Practices to prevent pooling of water in the periodomicile: filling plant pots or receptacles with sand; removing rubble; turning over pots and drinks bottles; wiping condensation down from surfaces, or other measures to encourage drainage and prevent stagnancy.
Behaviour adoption	Behaviour change attributed to ZIKV; including comments on increased or decreased frequency of an activity.
Delaying pregnancy	Decision to prevent or delay pregnancy, detailing methods that include use of contraceptives, non-penetrative sex, abstinence etc. Also referrals to members of the social circle or their wider network that delayed pregnancy. Excluded: abortion.
No change	Behaviours were practiced before ZIKV epidemic, or no adoption of preventive practices since the ZIKV epidemic.
**3.2 Community Participation**	Participant has engaged with others in the community, describing activities for collective action for vector control since the arrival of the ZIKV epidemic.
Collective Action	Engaging with others for activities specific to vector control, e.g. consulting with neighbours or community groups, exchanging advice with members of their immediate social circle.

Bancroft D, *et al. BMJ Open* 2022; 12:e050991. doi: 10.1136/bmjopen-2021-050991

Reporting	Reporting of sources of concern for mosquito control (e.g. communal spaces and garbage, larval growth) to landlords or building maintenance staff, local authorities, health agents or other third parties in position of power.

**4. COMMUNITY PREFERENCES**	Expressed needs or elaboration of preferences for mosquito-abatement products, or coordination of vector control strategies and health promotion related to ZIKV.
**4.1 Personal protection**	Novel topical mosquito repellents, repellent-impregnated clothing or other wearables (e.g. plastics) designed to repel and prevent mosquito bites.
Preferred criteria	Preferred criteria for novel repellents and repellent wearables that would encourage adoption, such as responses relating to comfort, appearance, affordability, effectiveness and other responses to question 5 of the topic guide.
Suggestions	Responses where participant mention a criterion for novel repellents not coded for in the other responses, e.g. suggestions for alternative repellent products (e.g. microencapsulated bracelets). Any other responses to question 5 of the topic guide.
**4.2 ZIKV Messaging**	Preferred risk communication and community engagement for MBD surveillance, mosquito bite-reduction and vector control strategies. Responses to: "Which of the Zika information sources do you think was the best and which was the least useful?"
Preferred delivery	Preferred format, frequency and source of delivery of risk communication (e.g. social media, in person).
Preferred target audience and messaging	Preferred target for risk communication and community engagement where participants express there is the most need (e.g. men, school children) and preferred key messages or specific topics related to ZIKV and MBDs.
Questions	Expressing lack of understanding or requests for clarification on topics related to ZIKV or other MBDs.
**4.3 Vector control**	Preferred activities for mosquito population control; perceptions of where the responsibility lies for vector control.
Community Level	Suggestions for action related to community groups, local authorities or within their local social network. e.g. health inspections or appointment of community members for capacity building and mobilisation of funding.
National Level	Preferred activities at the national level. For example, suggestions for action related to government policy and legislation, funding, public health campaigns or vaccine research and development.

Bancroft D, *et al. BMJ Open* 2022; 12:e050991. doi: 10.1136/bmjopen-2021-050991

**Discard pile**	Participant responses do not answer the topic guide questions or are considered relevant to the research question to justify creation of a new code.

1 Cislaghi B, Heise L. Theory and practice of social norms interventions: Eight common pitfalls. *Global Health* 2018;14(1):1–10.

2 Champion VL, Skinner CS. The Health Belief Model. In: Glanz, K., Rimer, B.K. & Viswanath, K., eds. Health Behavior and Health Education: Theory, Research, and Practice. San Francisco: Jossey-Bass 2008:45–66

3 Manca AR. Social Cohesion. In: *Encyclopedia of Quality of Life and Well-Being Research* Dordrecht: Springer Netherlands; 2014 [cited 2020 Sep 23]. p. 6026–8.

4 Carvalho MS, Honorio NA, Garcia LMT, *et al*. Aedes ægypti control in urban areas: A systemic approach to a complex dynamic. *PLoS Negl Trop Dis* 2017;11(7):1–15.

Bancroft D, *et al. BMJ Open* 2022; 12:e050991. doi: 10.1136/bmjopen-2021-050991
