# Supplementary material for: Vector control strategies in Brazil: a qualitative investigation into community knowledge, attitudes and perceptions following the 2015–2016 Zika virus epidemic
Source: BMJ Open. 2022 Jan 27;12(1):e050991. doi: 10.1136/bmjopen-2021-050991 (PMC8808399; doi:10.1136/bmjopen-2021-050991)
Supplement: Author's manuscript [file bmjopen-2021-050991.draft_revisions.pdf]

BMJ Open

BMJ Open is committed to open peer review. As part of this commitment we make the peer review history of every article we publish publicly available.

When an article is published we post the peer reviewers' comments and the authors' responses online. We also post the versions of the paper that were used during peer review. These are the versions that the peer review comments apply to.

The versions of the paper that follow are the versions that were submitted during the peer review process. They are not the versions of record or the final published versions. They should not be cited or distributed as the published version of this manuscript.

BMJ Open is an open access journal and the full, final, typeset and author-corrected version of record of the manuscript is available on our site with no access controls, subscription charges or pay-per-view fees (http://bmjopen.bmj.com).

If you have any questions on BMJ Open's open peer review process please email info.bmjopen@bmj.com

BMJ Open

Post-epidemic community knowledge, attitudes and perceptions of Zika virus and vector-control strategies in Brazil.

Journal:	*BMJ Open*
Manuscript ID	bmjopen-2021-050991
Article Type:	Original research
Date Submitted by the Author:	05-Mar-2021
Complete List of Authors:	Bancroft, Dani; London School of Hygiene & Tropical Medicine, Department of Public Health<br>Power, Grace; London School of Hygiene & Tropical Medicine, Department of Disease Control; University of Bristol, MRC Integrative Epidemiology Unit, Bristol Medical School<br>Jones, Robert; London School of Hygiene & Tropical Medicine, Department of Disease Control<br>Massad, Eduardo; University of Sao Paulo, School of Medicine; Fundacao Getulio Vargas<br>Iriat, Jorge Bernstein; Federal University of Bahia, Institute of Collective Health<br>Preet, Raman; Umeå University, Department of Epidemiology and Global Health<br>Kinsman, J; Umea University, Department of Epidemiology and Global Health<br>Logan, James; London School of Hygiene & Tropical Medicine, Department of Disease Control
Keywords:	Public health < INFECTIOUS DISEASES, Entomology < TROPICAL MEDICINE, Epidemiology < TROPICAL MEDICINE, Infection control < INFECTIOUS DISEASES

SCHOLARONE™
Manuscripts

I, the Submitting Author has the right to grant and does grant on behalf of all authors of the Work (as defined in the below author licence), an exclusive licence and/or a non-exclusive licence for contributions from authors who are: i) UK Crown employees; ii) where BMJ has agreed a CC-BY licence shall apply, and/or iii) in accordance with the terms applicable for US Federal Government officers or employees acting as part of their official duties; on a worldwide, perpetual, irrevocable, royalty-free basis to BMJ Publishing Group Ltd ("BMJ") its licensees and where the relevant Journal is co-owned by BMJ to the co-owners of the Journal, to publish the Work in this journal and any other BMJ products and to exploit all rights, as set out in our licence.

The Submitting Author accepts and understands that any supply made under these terms is made by BMJ to the Submitting Author unless you are acting as an employee on behalf of your employer or a postgraduate student of an affiliated institution which is paying any applicable article publishing charge ("APC") for Open Access articles. Where the Submitting Author wishes to make the Work available on an Open Access basis (and intends to pay the relevant APC), the terms of reuse of such Open Access shall be governed by a Creative Commons licence – details of these licences and which Creative Commons licence will apply to this Work are set out in our licence referred to above.

Other than as permitted in any relevant BMJ Author's Self Archiving Policies, I confirm this Work has not been accepted for publication elsewhere, is not being considered for publication elsewhere and does not duplicate material already published. I confirm all authors consent to publication of this Work and authorise the granting of this licence.

For peer review only

Title: Post-epidemic community knowledge, attitudes and perceptions of Zika virus and vector-control strategies in Brazil.

Version date: March 4, 2021

Manuscript word count: 5000 (excluding tables and legends: 557). Figure legends at end of reference list.

Abstract word count: 300

References: 71

Supplementary files: 1. Topic guide; 2. COREQ checklist; 3. Codebook.

Key words: Medical entomology, Public health, Arboviruses, Qualitative study.

Dani Bancroft [1,†], Grace M. Power [2, 3, †], Robert T. Jones [2], Eduardo Massad [4], Jorge Bernstein Iriat [5], Raman Preet [6], John Kinsman [6], James G. Logan [2 *]

[1] Department of Public Health, Environments and Society, Faculty of Public Health and Policy, London School of Hygiene & Tropical Medicine, London, United Kingdom; [2] Department of Disease Control, Faculty of Infectious and Tropical Diseases, London School of Hygiene & Tropical Medicine, London, United Kingdom; [3] MRC Integrative Epidemiology Unit, Population Health Sciences, Bristol Medical School, University of Bristol, United Kingdom; [4] School of Medicine, University of Sao Paulo and Fundacao Getulio Vargas, Brazil; [5] Institute of Collective Health, Federal University of Bahia, Brazil; [6] Department of Epidemiology and Global Health, Faculty of Medicine, Umeå University, Umeå, Sweden

[†]Contributed equally to this paper

*Corresponding author

Corresponding author details:

Robert T Jones – Robert.Jones@lshtm.ac.uk

London School of Hygiene & Tropical Medicine, Keppel Street, London, WC1E 7HT

United Kingdom

For peer review only

ABSTRACT

Objective:

The World Health Organization declared the 2015–2016 Zika virus (ZIKV) epidemic in Brazil and emergence of foetal microcephaly following prenatal infection a Public Health Emergency of International Concern. This triggered a national campaign to engage better control of the *Aedes* mosquito vector and uptake of ZIKV preventive behaviours, including use of topical mosquito-repellents. Achieving adherence to vector-control or mosquito-bite reduction strategies is challenging. Co-production of post-epidemic research at the community level is needed to understand and mitigate determinants of low ZIKV preventive measure uptake, particularly within disempowered groups.

Design:

In 2017, the Zika Preparedness Latin America Network (ZikaPLAN) conducted a qualitative study of focus groups and semi-structured interviews to understand barriers and likelihood of preventive behaviour change in response to ZIKV and other mosquito-borne disease outbreaks. Presented here is a thematic analysis of 33 interview transcripts, elaborating community knowledge, attitudes and perceptions of ZIKV and vector-control strategies by applying the Health Belief Model (HBM).

Participants:

123 purposively sampled members of the public; 106 women and 17 men of reproductive age (18–45)

Setting:

Two sociopolitically and epidemiologically distinct cities in Brazil: Jundiaí (57km north of São Paolo) and Salvador (Bahia state capital).

Results:

Four key and 12 major themes emerged from the analyses: (i) knowledge and cues to action; (ii) attitudes and normative beliefs (perceived threat, barriers, benefits and self-efficacy); (iii) behaviour change (household prevention and community participation); and (iv) community preferences for novel repellent tools, vector-control strategies and ZIKV messaging.

Conclusions:

Common barriers to repellent adoption were accessibility, appearance and effectiveness. Nationally, a health campaign targeting men is recommended, in addition to local mobilisation of funding for community volunteer, surveillance and risk communication capacity-building. A strong case is made for the transferability of the HBM to inform epidemic preparedness for mosquito-borne disease outbreaks at the community level.

For peer review only

STRENGTHS AND LIMITATIONS OF THIS STUDY

- There are few examples of direct post-epidemic engagement and research co-production with disempowered groups in Brazil, including pregnant women and communities with lower socioeconomic-position.
- Focus groups and semi-structured interviews provided rich qualitative data on perceptions of vector-control strategies at the community level at the time of the Zika outbreak, and barriers to the adoption of preventive actions.
- A large sample of community members of different age and sociodemographic position promoted generalisability of the study outcomes and recommendations.
- A limitation of focus groups is that some responses on more contentious topics may be influenced in a group setting.
- Since interviews took place in 2017, follow-up sessions may have provided greater understanding of how perceptions of *Aedes*-related diseases changed during the epidemic and have altered with more recent outbreaks of chikungunya and yellow fever in Brazil.

For peer review only

BACKGROUND

Zika virus (ZIKV) is a flavivirus primarily transmitted by *Aedes aegypti*, an aggressive day-biting mosquito found in tropical and subtropical climates.[1] Whilst ZIKV infection is often asymptomatic in adults, it is clinically recognised as causing a self-limiting 7–10 day febrile illness, and has been associated with serious neurological implications, including Guillain-Barré syndrome, encephalitis, and thrombocytopenia.[2–4] In November 2015, the Brazilian Ministry of Health (MoH) declared a national public health emergency due to suspected links between ZIKV and a sharp increase in congenital microcephaly, a rare foetal developmental disorder.[5] As ZIKV cases continued to rise, the World Health Organization (WHO) declared ZIKV a Public Health Emergency of International Concern in February 2016.[6] By February 2017, Brazil accounted for more than half of the confirmed cases of ZIKV in the Americas (n = 201,821), and for 90% of cases of Congenital Zika Syndrome (CZS) (n = 2,366), which includes neonatal microcephaly and other neurological impairments associated with prenatal ZIKV infection.[7,8]

The main line of defence against ZIKV is population control of *Ae. aegypti*.[9] Rapid or unplanned urbanisation has contributed to the metropolitan success of this species, which breeds in areas with poor drainage, waste accumulation and open sewers.[10] Negotiating responsibility for maintenance of communal spaces or failing to identify cryptic breeding sites prevents adequate vector-control.[11] Furthermore, chronic underfunding and intervention siloes undermine control efforts.[12] Individual-level mosquito bite-reduction strategies include wearing long-sleeved clothing to create physical barriers,[13] or applying topical repellents.[13] Non-topical strategies include fabric repellent or insecticide sprays.[15] However, many repellents do not provide long-lasting protection and require re-application.[14] Insecticide treatment of materials, as used for military clothing in some settings, may provide an effective and scalable prevention strategy.[10,16]

ZIKV can also be transmitted horizontally by sexual contact and blood transfusions.[17,18] To reduce sexual transmission of ZIKV and the risk of CZS, Brazil's MoH promoted condom use and postponing pregnancy during the epidemic.[6] Whilst international guidelines also advocated the relaxation of anti-abortion legislation,[19] in Brazil, abortion is only decriminalised for foetal anencephaly (a lethal birth defect), rape or conditions risking maternal death.[20] As a result, abortion was omitted from the MoH protocol on reproduction rights and pre-natal, delivery, and postpartum care in response to ZIKV.[21] Instead, Brazil's policy strategy emphasised vector-control, assurance of access to health care for individuals with ZIKV-related neurological conditions, and technology research and development.[22]

In November 2016, the WHO declared the end of the ZIKV epidemic.[6] However, there is no vaccine for ZIKV, and *Aedes* mosquitoes continue to transmit arboviruses worldwide.[23] The epidemic preparedness community emphasises the high risk of future outbreaks of ZIKV or other emerging mosquito-borne diseases (MBDs). Brazil's limited success at *Aedes* control suggests investigations into the social

For peer review only - http://bmjopen.bmj.com/site/about/guidelines.xhtml

determinants of low adherence to mosquito-bite preventive behaviours are required. [13,28,29] Despite this, there are few examples of direct post-epidemic engagement or research co-production with disempowered groups, including pregnant women, communities with lower socioeconomic position and those experiencing racial discrimination.[26]

Aims

This study aimed to gauge community knowledge, attitudes and perceptions towards ZIKV in two sociopolitically and epidemiologically distinct populations in Brazil: Jundiaí, a suburb of São Paulo with approximately 423,000 residents, and Salvador, the state capital of Bahia, population 2.9 million.[27] Additional study objectives were to: (1) elaborate household preferences for vector-control strategies; (2) identify perceived barriers to adoption of preventive behaviours; (3) contrast perceptions of ZIKV control over other mosquito-borne arboviruses; (4) compare normative beliefs of pregnancy postponement and abortion to reduce foetal susceptibility to CZS; and (5) map themes against a theoretical framework for behaviour change.

METHODS

Participant Recruitment and Data Collection

From March-August 2017, focus-group discussions (FGDs) with women of reproductive age (18–49) and semi-structured interviews (SSIs) with male partners were collected in Jundiaí and Salvador. Both cities have cohorts of CZS children.[28–30] The interview topic guide comprised of 12 questions covering three main areas of enquiry: (i) perceptions and practices of mosquito control, (ii) protecting oneself against mosquito bites, and (iii) knowledge and perceptions of ZIKV.(**Supplementary File 1**) All sessions were delivered in Brazilian Portuguese, and the source data transcribed and translated into English for analysis.

Participants

Participants were purposively sampled and consented to participate in the study. The pregnancy status of women was not taken into account and a sociodemographic survey stratified participants by age (18–30 or 31–49 years). In Jundiaí, recruitment took place in outpatient departments at University Hospital, and data collection in faculty buildings and an NGO-run community centre. In Salvador, recruitment and data collection took place in two Primary Care Units. In both cities, men were recruited through community stakeholders and interviewed at private residences.

Patient and Public Involvement

The principal investigators from Jundiaí and Salvador are native Brazilian speakers familiar with the study setting and context. To ensure the research question was informed by patients' priorities and experiences, it was developed through pilot testing of the topic guide with research teams local to the study sites. In-depth interviews with health professionals and community leaders were held, including with health professionals

For peer review only - http://bmjopen.bmj.com/site/about/guidelines.xhtml

in Salvador working in a Primary Care Unit and in private clinics, and with three religious leaders from Kardecism, Candomblé (an Afro-Brazilian religion), and an evangelical Christian church. To disseminate results, those who expressed interest and provided consent had their contact details collected and were invited to attend a follow up session to discuss initial findings in September 2017.

Analysis

In total, 33 transcripts were analysed.(**Table 1**) Open coding was performed in NVivo (version 12, QSR International). Theme generation followed Braun and Clarke's six phases for thematic analysis.[31] A preliminary coding framework was established from the topic guide. However, coding was mostly inductive, by grouping prevalent response patterns into higher-order categories.[32] Major themes were mapped against the constructs in Rosenstock's Health Belief Model (HBM),[33,34] a widely adopted theoretical framework for behaviour change that has been applied to other MBD studies.[35,36] A concept map for themes was developed to gauge whether there was a credible fit with the HBM.(**Figure 1**) The 32-item Consolidated Criteria for Reporting Qualitative Research (COREQ) tool was used to ensure all key methodological issues were taken into account.[37](**Supplementary File 2**)

Table 1: Units of analysis. A total of 17 focus-group discussions (FGD) and 16 semi-structured interviews (SSI) were included in the analysis. Three FGDs were missing sociodemographic data (age). Unit 9 was selected for triangulation. Unit 18 was a deviant case excluded from the analysis.

Unit		Words	Age	Unit		Words	Age
1	Jundiaí-FGD1	4,338	18–30	19	Salvador-FGD1	14,762	31–49
2	Jundiaí-FGD2	4,399	31–49	20	Salvador-FGD2	3,318	18–30
3	Jundiaí-FGD3	4,067	18–30	21	Salvador-FGD3	16,863	31–49
4	Jundiaí-FGD4	3,409	31–49	22	Salvador-FGD4	10,262	18–30
5	Jundiaí-FGD5	1,691		23	Salvador-FGD5	8,103	18–30
6	Jundiaí-FGD6	4,026	31–49	24	Salvador-FGD6	15,619	31–49
7	Jundiaí-FGD7	1,239		25	Salvador-FGD7	13,138	31–49
8	Jundiaí-FGD8	3,012	31–49	26	Salvador-FGD8	9,256	18–30
**9**	**Jundiaí-FGD9**	**1,860**					
10	Jundiaí-SSI1	41		27	Salvador-SSI1	619	
11	Jundiaí-SSI2	44		28	Salvador-SSI2	346	
12	Jundiaí-SSI3	37		29	Salvador-SSI3	208	
13	Jundiaí-SSI4	65		30	Salvador-SSI4	407	
14	Jundiaí-SSI5	73		31	Salvador-SSI5	269	
15	Jundiaí-SSI6	147		32	Salvador-SSI6	367	
16	Jundiaí-SSI7	276		33	Salvador-SSI7	298	
17	Jundiaí-SSI8	105		34	Salvador-SSI8	239	
**18**	**Jundiaí-SSI9**	**4,312**	**18–30**				

For peer review only - http://bmjopen.bmj.com/site/about/guidelines.xhtml

RESULTS

A total of 120 individuals participated in the study: 103 women (60 in Jundiaí, 43 in Salvador); and 17 men. Salvador focus-groups had higher engagement than those in Jundiaí (**Table 1**) Responses to questions on novel repellents were initially coded: effectiveness; affordability; availability; appearance; comfort; protection; risk; and other. These were mapped against the HBM as: risk (perceived susceptibility); positive responses, such as protection (perceived benefits); willingness to adopt (self-efficacy); negative responses for effectiveness, acceptance or accessibility (perceived barriers); and alternative suggestions (preferred criteria). The finalised concept map comprised of 44 minor themes and 12 major themes grouped under four higher-order key themes.(**Figure 2**; **Table 2**) Definitions are provided in the codebook.(**Supplementary File 3**).

For peer review only

For peer review only - http://bmjopen.bmj.com/site/about/guidelines.xhtml

Table 2: Summary table of definitions for key and major themes.

Theme		Definition
**1.**	**Knowledge and Cues to Action**	Depth of understanding of ZIKV, MBDs, vector-control, and recalled key messages. Stimuli for a decision-making process that may have led to behaviour change, as recalled at the time of study.[38]
1.1	Knowledge of MBDs	Participant awareness of MBDs and ZIKV, as well as the community and national response to outbreaks at the time of the study.
1.2	External Cues to Action	External stimuli, such as a health campaign, triggered a decision-making process for a behaviour change.
1.3	Internal Cues to Action	Direct and indirect experiences of confirmed or suspected cases of MBDs triggered a decision-making process that leads to behaviour change.
**2.**	**Attitudes and Normative beliefs**	Personal attitudes are internal assessments of knowledge and cues to action for MBD preventive behaviours. Normative beliefs may inform personal attitudes according to how others perceive the behaviour in a social setting, such as the community.[57]
2.1	Perceived Susceptibility	A subjective assessment of risk of ZIKV infection or a CZS pregnancy. The first component of perceived threat.[38]
2.2	Perceived Severity	A subjective assessment of the severity of ZIKV symptoms and CZS. The second component of perceived threat.[38]
2.3	Perceived Barriers	An individual's assessment of the obstacles to ZIKV preventive behaviours for sexual transmission, mosquito bite-reduction and vector-control.
2.4	Perceived Benefits and Self-efficacy	An individual's perception of the benefits of novel repellent technologies and their ability to successfully undergo a behaviour change to adopt new preventive strategies.[38]
**3.**	**Behaviour Change**	Behaviours either attributed to the ZIKV epidemic, are pre-existing practices against MBDs (no change), or no preventive measures were taken.
**3.1**	Household level	Practices to prevent mosquitos from breeding and exposure to mosquito bites at the household.
**3.2**	Community Participation	Engaging with others in the community; describing activities for collective action for vector-control.
**4.**	**Community Preferences**	Expressed needs and preferences for mosquito bite-reduction strategies, coordination of vector-control and ZIKV messaging, including questions.
4.1	Novel Repellents	Preferred criteria for novel topical mosquito repellents, repellent-impregnated clothing or other wearables designed to prevent mosquito bites.
4.2	Vector-control strategy	Preferred activities for mosquito population control, including surveillance.
4.3	ZIKV messaging	Preferred content, source and format for delivery of ZIKV risk communication and community engagement.

For peer review only - http://bmjopen.bmj.com/site/about/guidelines.xhtml

Knowledge and Cues to Action

Participants expressed uncertainty around which vectors transmit ZIKV. More participants could describe *Ae. aegypti* in Salvador, but not all could differentiate the mosquito from other biting insects. Dengue was the second most commonly discussed MBD, although chikungunya and yellow fever were also discussed. Most participants were aware of ZIKV's impact on pregnancy. However, sexual transmission of ZIKV was poorly understood, and questions from women that disclosed higher levels of education often related to the pathophysiology of ZIKV and unknown sequalae.

> *[P1]: So, [microcephaly] sparked people's interest: "Pow, then really, that's the difference between Zika and dengue and H1N1."*
>
> **Salvador-FGD1**

> *[P2]: [ZIKV is transmitted by] the host, yes. But not from person to person…This has not been clear to me until today.*
>
> **Salvador-FGD3**

Many women first learned about ZIKV and were advised to use condoms when accessing maternity services. Often exposure to public health information in broadcast or print media, including pamphlets and posters, was described. Several mentioned learning about ZIKV online, via social media, the workplace or higher-education. Other external cues to action included direct contact with political representatives, NGOs, or community volunteers involved with Zika projects. Health agents were described to inspect households and disseminate health information about *Aedes* and preventive strategies. One key message recalled was to remove standing water; participants from four FGDs also recalled a visit from military personnel to promote clearing communal spaces.

> *There was a joint effort that the government [made] in the neighbourhood, like this... It was like D-Day against Zika, dengue…*
>
> **Salvador-FGD4**

Visibility of vehicle-mounted thermal spraying/fogging in previous years was recalled by several groups, although most activities were described as having ended. Most agreed that ZIKV messaging had slowed or stopped at the time of their interview, and several participants recalled no community vector-control interventions at all. Internal cues to action comprised direct or indirect experiences of confirmed/suspected cases of MBDs. In Salvador more women had experience of ZIKV, whereas in Jundiaí few participants knew someone infected.

For peer review only - http://bmjopen.bmj.com/site/about/guidelines.xhtml

> *[P1]: I think [during] the outbreak I [became] more attentive…everyone was contracting Zika…Wow! My father had it too, and he had that anxiety thing – if you saw anything, even if it had water in a little while, you'd turn it [upside down].*
>
> **Salvador-FGD4**

Attitudes and Normative Beliefs

There was consensus across all groups that pregnant women were most susceptible to ZIKV infection, followed by children, the elderly and those with chronic diseases. Participants described avoiding travel to areas perceived to have elevated risk of MBDs and some understood outbreak seasonality. Several described the belief that infection by one MBD increased their susceptibility to others, although there was a lot of uncertainty and misinformation around ZIKV case confirmation. The spread of misinformation was a concern to participants, and several misinterpreted or distrusted information about the origin of the virus.

> *[P2]: In my opinion, I knew that Dengue and Zika is the same thing…I think that's evolution from one disease to another.*
>
> **Salvador-FGD6**

Living in an area of perceived low risk commonly diminished participants interest in adopting preventive measures ("*It's only worrisome when there's an epidemic*," **Jundiaí-FGD1**). However, there was less consensus between FGDs regarding where population density of *Ae. aegypti* vectors was highest, and several participants described their absence from their neighbourhood altogether. Perceived severity of ZIKV infection also varied considerably. Some likened ZIKV symptoms to mild influenza, although women perceived a higher threat from ZIKV than men, recalling inflammation of the joints and fatigue with extended recovery times, even risk of death.

> *[P1]: It caused a panic, right? Many women gave up being mothers, or they delayed, right? Fear of disease.*
>
> *[P2]: In fact, all the [mosquito-borne] diseases mentioned are worrisome, right? Even the flu is scary.*
>
> **Salvador-FGD8**

Some participants also described differences in bites from mosquitoes carrying ZIKV as eliciting an allergic reaction, perhaps a perception of the maculopapular rash. Several women demonstrated higher awareness of ZIKV sequelae from working or study in health care, or volunteering with local ZIKV projects. Although collective awareness was described to have peaked and waned, several participants commented on the visibility of families caring for a child with CZS in broadcast media, and they believed a greater disease burden was in more deprived communities.

For peer review only - http://bmjopen.bmj.com/site/about/guidelines.xhtml

...usually the people most affected [by CZS] are low-level people right...people who have poor conditions, who live in more inappropriate places.

Jundiaí-Male-SSI-8

Several participants disclosed they would be willing to access abortion services to reduce risk of having a child with CZS, or having previously terminated a pregnancy, but perceptions of rights to terminate a pregnancy were influenced by strong social norms and religious beliefs, and there was often reluctance to disclose or elaborate on personal attitudes due to its criminalisation. Some conceded community attitudes and norms were more nuanced surrounding perceptions of quality-of-life and severe disability with CZS. However, for one group, partial legislation of abortion in the case of microcephaly was criticised as inadequate, or perpetuating discrimination.

...I think it depends on where she congregates because religion weighs in a lot...She will not do it because of religion, and if she dares [abort], she will not be accepted.

Jundiaí-FGD4

Women aged 18–30 were more supportive of the right to abort, as were participants that disclosed as working in health or having accessed higher-education. Despite adequate levels of perceived threat from ZIKV and recognition of potential benefits of a behaviour, participants described many barriers to reproductive health decision-making. There was frustration about the burdens of preventing ZIKV and caring for children with CZS falling on women. Discordant attitudes towards abortion between pregnant women and male partners were also discussed. For example, women reported diminished self-efficacy to negotiate condom use with an intimate partner during the epidemic, attributed to stability of the relationship or harmful gender norms:

[P2]: We've already talked about machismo, right? I've heard of a husband dropping his wife and saying "No, if you do not [abort], I'll let you go," because she already knew she had a microcephaly [baby].

…

[P3]: Yes, but the question of the condom? OK! One part would accept, but this question of non-penetrative sex for six months? No!

Salvador-FGD5

Another barrier was low participant awareness and accessibility of repellent clothing. Owning a single item was not perceived to provide sufficient protection, yet buying 'a whole wardrobe' a significant investment. Interest was also strongly affected by their appearance in the community, such as smell, fabric quality and design. In both cities, references were made to repellents being less accessible for individuals of lower-socioeconomic position.

For peer review only - http://bmjopen.bmj.com/site/about/guidelines.xhtml

[P4]: It's just one more expense, right? [ALL]: Yes!

[P5]: It would probably be very expensive. Because it would be, say, for the elite.

Salvador-FGD5

Maybe he is bothered about having to wear clothes that would be, in this case, also an indicator of poverty, right?

Male-Salvador-SSI-4

For several participants, skin allergies were a barrier for topical repellents, whereas for some this motivated investment into alternative brands or non-chemical bite-reduction strategies. A common benefit of repellent clothing was protection of children and pregnant women, although overheating was a concern. Overall, there was a positive reception to adoption of novel repellent tools if they were affordable, looked and smelled nice. However, similarity was observed between shorter responses and interviewer prompts, and men often expressed disinterest. Scepticism around long-term effectiveness of repellent clothing was also observed, including concern for areas of skin left exposed.

[P4]:…an entire population can't be protected that way. In particularly endemic regions and for high-risk group like babies or pregnant [women] it works, but it's not good for you to dress a whole neighbourhood with the same clothes!

Salvador-FGD4

At the community level, contextual factors were often linked to MBD outbreaks, such inadequate coverage of urban planning, e.g. sanitation services. In Salvador, the former administration was criticised for poor management of the ZIKV epidemic, including the cost of testing, financial support for families with CZS children and an over-reliance on mass-media campaigns. Surveillance teams were often perceived as undermotivated, not being trusted to search for cryptic breeding sites, or failing to enter all households due to neighbourhood violence.

[P1]: Where are the community agents themselves? I'm not talking about treatment, I'm talking about preventive measures. Community agents are not effective by municipal power…it's a type of unstable work, you know? There are months without receiving [them].

Salvador-FGD3

There is a lot of suspicion…total distrust in the [Zika] project…The resistance with men is great.

Jundiaí-FGD4

For peer review only - http://bmjopen.bmj.com/site/about/guidelines.xhtml

Behaviour Change

The most frequent vector-control strategy described by participants at the household level was preventing water stagnating by recycling, using sand, covering receptacles, applying detergents or insecticides. Bite-reduction strategies included physical barriers: fans, air-conditioning, bed-nets, window screens, long clothing. Several described using plug-in appliances or burning coils to repel mosquitoes with increased frequency during the epidemic. Electric-shock devices to kill adult mosquitoes were also popular. Some avoided travel to places or during times when mosquitoes are most active. Women in every focus-group described knowing someone in their social circle having delayed pregnancy to mitigate risk of CZS.

> *I have two sisters-in-law who wanted to get pregnant, but because of the epidemic they were afraid and postponed it.*
>
> **Jundiaí-FGD3**

Community participation comprised reporting mosquito breeding sites to public health authorities, which was frequently discussed in Jundiaí. Several women described generally observing and encouraging behaviour change in others, including use of repellents.

> *[P1]: …it's not just the authorities, everyone has to do their part…to be able to openly reach the neighbour and say, "Oh, look at your bottles [they're] full of water, focus."*
>
> **Salvador-FGD8**

Some participants described skin irritation from topical repellents, but only one participant recalled women avoiding chemical repellents during pregnancy. Methods for mixing plant-based oils or alcohol with chemical formulations and sunscreen were described to soothe and prevent bites from becoming infected, mask product smell and reduce cost of re-purchase.

> *[In]Bahia, the desperation is greater than here, and pregnant women are afraid to use any product and use homemade products [instead]…*
>
> **Jundiaí-FGD2**

Community Preferences

Subsidy of contraceptives and repellents were suggestions for lower-income or high-risk groups during outbreaks, or as gifts-in-kind from local health clinics, NGOs or the national social welfare program, Bolsa Família. Repellent school uniforms to reduce children's risk of MBDs were of interest, as was repellent sleepwear to mitigate discomfort from bednets or topical repellents. However, conforming to fashion and renewing effectiveness of existing clothing was important. Microencapsulation of repellents in wearable plastics were also suggested by participants, such as bracelets.

For peer review only - http://bmjopen.bmj.com/site/about/guidelines.xhtml

For peer review only

[P4]: The government should give repellent to the people since you have this yellow fever outbreak. Make a campaign. The same people who have family-grants should be entitled…

Salvador-FGD6

[P1]: …you would have to change your wardrobe to buy only mosquito repellent clothes. It would be [a] more effective process [if] you make your clothes have this substance.

[P2]: It makes more sense. Like a lotion.

[P1]: A lotion that you put on to do laundry...

[P3]: Yeah, like a fabric softener.

Salvador-FGD4

For vector-control, often improved City Hall management was expressed as a priority need, citing open drains or infrequent collection of household waste. One focus-group was interested in reintroducing thermal spraying of insecticides. Another explored the idea of financing the coordination of neighbourhood associations to mobilise the community, including financial compensation of volunteers.

[P1]: How are we going to complain about our problems? We do not have a person who can get there and settle for us. If we make a petition, everybody in the neighbourhood will sign, but who will take it?…our neighbourhood is abandoned, we have no association…

[P2]: I think every neighbourhood should have [an association].

[P1]: [The former volunteer] did everything for us there. My street was clean, everything was clean. There should be someone to count, take care, understand?

[P3]: If she's doing it, she has to get something too...

[P4]: But the staff thinks the person [must] work for free.

Salvador-FGD6

There was disagreement regarding saturation of ZIKV key messages. The majority of women expressed feeling under-equipped, whereas others asserted technical information was not a sufficient call to action. Preference was placed on sustained delivery of messages between outbreaks, via social media or health promotion materials in public spaces. A campaign targeting men was suggested to escalate perceptions of health consequences of ZIKV and sexual transmission. A sexual and reproductive health curriculum for schools and community events was suggested to improve participatory engagement with messaging and amplify the effect of annual campaigns like 'World Dengue Day'.

No, it's not a lack of information, it's education…it has to start very early with sex education. Because human beings only change their habits when something very serious happens. I think information alone does not [do it].

Jundiaí-FGD3

For peer review only - http://bmjopen.bmj.com/site/about/guidelines.xhtml

[P4]: If it's not in the extreme, [messaging] will not work. It's like cigarette campaigns.
Salvador-FGD5

DISCUSSION

In the outbreak beginning 2015, Brazil experienced more cases of ZIKV than any other country. Its MoH responded with a policy strategy focused on vector-control, provision of health care access, and technology and research development. However, it has been argued that these policies failed to reach those most vulnerable.[22] The northeast of Brazil was particularly hard-hit, as a region with some of the lowest state Human Development Indices (HDI) in the country, a composite measure of indicators for life expectancy, education and Gross National Income per capita.[40] In comparison, in 2017 Jundiaí was ranked has having the 11th highest HDI in Brazil out of 5,564 municipalities.[30] Individuals from communities in Salvador and Jundiaí were invited to provide their knowledge and perceptions of ZIKV and MBD control.

The sessions revealed that participant understanding of their susceptibility to infection was a key influence on their decision-making to engage in protective behaviours. Direct or indirect experience of ZIKV and dengue was a common internal cue to action in Salvador, a city with a long history of MBDs,[41] which is consistent with previous findings.[29,42] However, how some participants thought there was not any ZIKV in their area, and perceptions varied as to where in Brazil the prevalence of this and other MBDs was greatest. At the time of the study, a national yellow fever vaccination campaign was communicating outbreaks in non-human primates,[43] and some participants discussed fearing its urbanisation.[6] Participants describing a potential relationship between ZIKV and other MBDs was not unwarranted, as arboviruses transmitted by *Aedes* tend to cluster.[12] Sequential arboviral infection is also still poorly understood,[44] with some studies suggesting limited cross-immunity following dengue infection.[45,46]

The majority of women interviewed were unaware of the risk of ZIKV transmission from unprotected sex. This is consistent with findings in other studies on ZIKV risk communication, including in Colombia.[25] Since interviews were conducted towards the end of the outbreak, this suggests there was a missed opportunity to prevent at least some of the spread of ZIKV. Although the ultimate importance of sexual transmission may be small compared to that of mosquito-borne transmission,[47] the public should receive clear messaging around the relative contributions of mosquito-borne, vertical, sexual, and bloodborne transmission, so individuals can make informed choices about adopting preventive measures.

MBDs, including ZIKV, predominantly affect individuals in economically deprived areas.[29,48,49] Inadequate access to clean water, sanitation and other infrastructural deficits allow mosquito populations to thrive,[26] and individuals in these communities are also less able to afford tools for personal protection, and may have poor access to good quality health care. In our focus groups, the perceived severity of ZIKV

For peer review only - http://bmjopen.bmj.com/site/about/guidelines.xhtml
For peer review only

was most often framed through the lens of disadvantage: the availability and affordability of amniocentesis or ZIKV testing; female agency to negotiate abstinence or long-term condom use with their male partners; access and acceptance of contraceptives to delay pregnancy or abortion, and uncertainty around a financial and social support network to care for children with CZS. These themes were consistent with other study findings.[40,50–52]

There was also strong disagreement around the criminalisation of abortion, which has been dismissed as a paternalistic policy inconsistent with MoH advice to avoid or delay pregnancy in ZIKV endemic areas.[53] The sense that ZIKV has been emasculated, where the responsibility to prevent sexual transmission has fallen to women, has also been described in other studies.[20,54,55] Despite being strongly advocated by international multi-lateral agencies and Brazilian legislators,[56] important questions remain outstanding on reproductive health rights for ZIKV seropositive individuals.[57]

Perceptions of Novel Repellents

Topical repellents are uncomfortable for some users, and may not be seen as long-term solutions for preventing mosquito bites.[15,36,58,59] The pay-off for adherence to the repeated use of repellents may also be less certain for ZIKV than other MBDs, where the onset of symptoms and potential consequences of infection is comparatively short.[60] Novel, non-topical repellent technologies are not yet widely known or understood, and perceived safety of synthetic repellents was anticipated to be a key barrier to their adoption, as seen in other qualitative studies.[25,36] Instead, the key barriers discussed were the effectiveness and accessibility of novel products.

In Salvador, it was also important that repellent clothing was not perceived to be a 'uniform' associated with low-socioeconomic position, whilst in Jundiaí, participants discussed the need for clothing designs to reflect local preferences in fashion. The concept of repellent school uniforms to protect school-going children from MBDs was well received, and has demonstrated strong potential in a cluster randomised controlled trial in Thailand.[10] Participants expressed an interest in being able to renew the repellent effect of clothing to overcome barriers like affordability and durability, negating a need for replacements. For example, using sprays to reapply repellents to clothing was perceived as more feasible than clothes treated prior to purchase. Some also acknowledged the attractiveness of formulated washes for ease of application, and incorporation of perfumes to mask repellent smell.

Community Prevention of Zika Transmission

Mosquito prevention at the household level was often perceived to be a burden. However, many participants described removal or treatment of potential mosquito-breeding sites as being incorporated into daily routines. Despite this, several individuals expressed their personal control beliefs for vector-control were fatigued when neighbours did not also do their part. Abandoned buildings or communal space 'contaminating' maintained areas contributed to some participants' sense of futility; even if they were well-

For peer review only - http://bmjopen.bmj.com/site/about/guidelines.xhtml
For peer review only

informed, a public health challenge as prevalent as *Aedes* was not something the community could 'combat' alone. This was also evident in Peru,[54] where both men and women expressed a need for intensification of government support.

Minor themes of blame, mistrust and responsibility were also frequently allocated upstream, especially in Salvador. Poor access and quality of free health clinics was often attributed to chronic under-investment, a common theme in other studies.[12,61] Likewise, reporting to the City Hall mosquito-breeding sites in communal areas in more deprived neighbourhoods was deemed unlikely to result in change due to broader inadequacies in local urban planning. Some participants were also frustrated by a lack of consistent and thorough household inspections by surveillance teams, confusion over the different stakeholders involved, and follow-up visits or clarifications needed for ZIKV messaging.

Risk Communication and Community Engagement Related to ZIKV Prevention

Freire posits that structural inequalities in Brazil creates a loss of agency,[62] which constrains self-efficacy for behaviour change.[39] A systems model for *Aedes* vector-control also argues that the pathway between collective awareness, collective action, community attitudes and normative beliefs is simply too long for effective control of MBD outbreaks.[64] The opportunity to communicate barriers in a more timely manner would improve collective awareness, as well as political will for local authorities to act.[12] Carvalho *et al.* proposed one solution could be investing in improved frequency of household visits from community health workers (CHWs) under the Family Health Strategy,[64] which covers 66.5% of Brazil's population.[65] Although task-shifting of CHW responsibilities to include ZIKV case reporting was possible during the epidemic, their catchment area excludes middle or high-income neighbourhoods,[66] like Jundiaí. Instead, a community-participation model is proposed as an alternative. Grassroots approaches, such as neighbourhood associations, may serve as a more trusted point of contact for community engagement during infectious disease outbreaks.[12,67] In a meta-analysis on uptake of novel repellent technologies,[68] participatory models were found most effective at improving self-efficacy.[69] Decentralising responsibility and triage of risk communication would also mitigate the marginalisation of individuals in more deprived settings caused by top-down approaches in health promotion.[62]

Limitations

Interview prompts around preferences for novel repellents may have enabled acquiescence response bias from participants.[31] Personal attitudes may have also been conflated with social norms when focus groups discussed more contentious topics, such as abortion, where some women may have felt unable to disclose disagreement with the majority.[39] Although facilitators were able to detect non-verbal cues for each, subtext may have been lost during analysis. To mitigate this, an independent translation service was used to verify the credibility of transcript excerpts, and the preliminary findings discussed with principal investigators for triangulation. One disadvantage of selecting the HBM as a conceptual framework is

For peer review only - http://bmjopen.bmj.com/site/about/guidelines.xhtml

disagreement over which order the components lead to behaviour change.[33,38] The literature was therefore consulted post-analysis for transferability of findings.[31]

Recommendations

This investigation recommends that in settings where MBD outbreaks are regular occurrences national authorities provide effective repellent tools to families entitled to social-welfare, and during outbreaks extend this provision to include high risk groups. Capacity-building of MBD surveillance teams is also recommended to strengthen multi-level governance and mitigate gaps in frequency of interventions to prevent infectious disease transmission, such as household inspections. A degree of data saturation for preferred criteria of novel repellents in this study lends weight to the finding there was an unmet need for personal protective tools like topical repellents.

The WHO Global Vector Control Response advises cross-disciplinary community engagement to improve context-sensitive messaging and reduce barriers to uptake of MBD preventive strategies.[27] Designing a mass-media campaign targeting men would improve awareness of ZIKV sexual transmission and highlight for them the importance of protecting the health of their female intimate partners. Further focus group studies, or design of a Likert scale-based survey that operationalises the HBM during data collection,[38] may also prove fruitful for understanding how perceived severity and susceptibility to MBDs has changed in Salvador and Jundiaí following outbreaks of chikungunya and yellow fever.[6,70]

Financing participatory models for community engagement would demonstrate a firm commitment to translating politicised slogans into an effective, bottom-up control strategy for *Aedes*-related MBDs. It is worth noting our recommendations are also pertinent to the response to the SARS-CoV-2 pandemic. Brazil has amongst the highest numbers of confirmed COVID-19 cases in the world, and its MoH has been criticised for not developing a national plan to combat the disease.[71] Internationally sanctioned public health measures, such as social isolation and mask use, should be mandated nationally, with additional assurances to protect pregnant women.[68]

Conclusion

Since the initial outbreak in Brazil in 2015, the fall of the perceived threat from ZIKV, normalization of CZS symptoms and poorly understood relationship to other viruses transmitted by *Aedes* has weakened community self-efficacy and perceptions of the government response. It is argued that the historical failure to control *Aedes* outbreaks in Brazil lies in placing too much responsibility on the individual, particularly women. This study makes a strong case for the value of qualitative investigations and transferability of the HBM to inform bottom-up approaches in health protection. By investing in evidence-based epidemic preparedness, and by stimulating a sense of community agency, Brazil may indeed be better placed to 'beat' the *Aedes* mosquito.

For peer review only - http://bmjopen.bmj.com/site/about/guidelines.xhtml

Acknowledgements

We would like to thank all study participants, and the ZikaPLAN research teams in Salvador and Jundiaí: Ana Maria Rico, Greice Bezerra Viana, Fernanda Macedo da Silva Lima, Mônica Manir, Tania Boccia and Vera Lucia Zaher-Rutherford. We would also like to thank Alexandra Levitas for her support during analysis.

Contributors

JGL conceived the study. JBI and EM led data collection in Salvador and Jundiaí, coordinated by JK. DB led the analysis and the University College London Digital Media service was used to translate select excerpts of Brazilian transcripts for verification against the translations made by EM. GMP and RTJ performed triangulation of coding. DB, GMP and RTJ authored the manuscript for publication. All authors read and approved the final manuscript.

Funding

This study was financed by the European Union's Horizon 2020 research and innovation programme awarded to Zika Preparedness Latin American Network, (ZikaPLAN) under Grant Agreement No. 734584

Competing interests

All authors declare no competing interests.

Patient consent for publication

Informed written consent was obtained before data collection. Additional consent for publication is not required.

Ethics approval

Approval for the study in both Jundiaí and Salvador was granted by the Jundiaí School of Medicine Ethical Review Board in January 2017 (Ref: 1.875.618). For analysis, approval was granted by the MSc Research Ethics Committee at LSHTM in July 2020 (Ref: 21978).

Data availability statement

The topic guide, figures and COREQ checklist supporting the conclusions of this article are included within the article and its supplementary files. The consent form and topic guide are available at the London School of Hygiene & Tropical Medicine (LSHTM) Data Compass repository [https://doi.org/10.17037/DATA.00002097]. The transcripts of focus-groups and semi-structured interviews supporting the conclusions of this article cannot be made available for confidentiality reasons.

For peer review only - http://bmjopen.bmj.com/site/about/guidelines.xhtml

References

1 Kauffman EB, Kramer LD. Zika Virus Mosquito Vectors: Competence, Biology, and Vector Control. *J Infect Dis* 2017;216:976–90. doi:10.1093/infdis/jix405

2 Brasil P, Pereira JP, Moreira ME, et al. Zika virus infection in pregnant women in Rio de Janeiro. *N Engl J Med* 2016;375:2321–34. doi:10.1056/NEJMoa1602412

3 Panchaud A, Stojanov M, Ammerdorffer A, et al. Emerging role of Zika virus in adverse fetal and neonatal outcomes. *Clin Microbiol Rev* 2016;29:659–94. doi:10.1128/CMR.00014-16

4 Wahid B, Ali A, Waqar M, et al. An updated systematic review of Zika virus-linked complications. *Asian Pac J Trop Med* 2018;11:1–8. doi:10.4103/1995-7645.223527

5 Wen Z, Song H, Ming GL. How does Zika virus cause microcephaly? *Genes Dev*. 2017;31:849–61. doi:10.1101/gad.298216.117

6 Epidemiological Alerts and Updates: Annual Report 2017. Washington D.C: 2018. www.paho.org/en/documents/epidemiological-alerts-and-updates-annual-report-2017

7 Zika suspected and confirmed cases reported by countries and territories in the Americas cumulative cases, 2015-2017. Washington D.C: Pan American Health Organization and World Health Organization 2017 [Update] 4 January, 2018. www.paho.org/hq/index.php?option=com_content&view=article&id=12390:zika-cumulative-cases (accessed 6 Dec 2020).

8 Holtzman M, Golden WC, Sheffield JS. Zika Virus Infection in the Pregnant Woman. *Clin Obstet Gynecol* 2018;61:177–85. doi:10.1097/GRF.0000000000000343

9 Handbook for Integrated Vector Management in the Americas. Washington, D.C.: Pan American Health Organization 2019.

10 Kittayapong P, Olanratmanee P, Maskhao P, et al. Mitigating Diseases Transmitted by Aedes Mosquitoes: A Cluster-Randomised Trial of Permethrin-Impregnated School Uniforms. *PLoS Negl Trop Dis* 2017;11:1–12. doi:10.1371/journal.pntd.0005197

11 Achee NL, Grieco JP, Vatandoost H, et al. Alternative strategies for mosquito-borne arbovirus control. *PLoS Negl Trop Dis* 2019;13:1–22. doi:10.1371/journal.pntd.0006822

12 Bardosh KL, Ryan S, Ebi K, et al. Addressing vulnerability, building resilience: Community-based adaptation to vector-borne diseases in the context of global change. *Infect. Dis. Poverty* 2017;6:166. doi:10.1186/s40249-017-0375-2

13 Orsborne J, Banks SDR, Hendy A, et al. Personal protection of permethrin-treated clothing against aedes aegypti, the vector of dengue and zika virus, in the laboratory. *PLoS One* 2016;11:1–18. doi:10.1371/journal.pone.0152805

14 DeRaedt Banks S, Orsborne J, Gezan SA, et al. Permethrin-Treated Clothing as Protection against the Dengue Vector, Aedes aegypti: Extent and Duration of Protection. *PLoS Negl Trop Dis* 2015;9:1–16. doi:10.1371/journal.pntd.0004109

15 Banks SD, Murray N, Wilder-Smith A, et al. Insecticide-treated clothes for the control of vector-borne

For peer review only

For peer review only - http://bmjopen.bmj.com/site/about/guidelines.xhtml

diseases: A review on effectiveness and safety. *Med Vet Entomol* 2014;28:14–25. doi:10.1111/mve.12068

16 Wilder-Smith A, Preet R, Brickley EB, et al. ZikaPLAN: addressing the knowledge gaps and working towards a research preparedness network in the Americas. *Glob Health Action* 2019;12:1666566. doi:10.1080/16549716.2019.1666566

17 Turmel JM, Abgueguen P, Hubert B, et al. Late sexual transmission of Zika virus related to persistence in the semen. Lancet 2016;387:2501. doi:10.1016/S0140-6736(16)30775-9

18 Musso D, Stramer SL, Busch MP. Zika virus: A new challenge for blood transfusion. Lancet 2016;387:1993–4. doi:10.1016/S0140-6736(16)30428-7

19 Collucci C. Brazilian attorneys demand abortion rights for women infected with Zika. *BMJ* 2016;354:i4657. doi:10.1136/bmj.i4657

20 Carabali M, Austin N, King NB, et al. The Zika epidemic and abortion in Latin America: a scoping review. *Int J Infect Dis* 2018;73:297. doi:10.1016/j.ijid.2018.04.4091

21 Lowe R, Barcellos C, Brasil P, et al. The zika virus epidemic in brazil: From discovery to future implications. *Int J Environ Res Public Health* 2018;15:96. doi:10.3390/ijerph15010096

22 Gómez EJ, Perez FA, Ventura D. What explains the lacklustre response to Zika in Brazil? Exploring institutional, economic and health system context. *BMJ Glob Heal* 2018;3:e000862. doi:10.1136/bmjgh-2018-000862

23 Wilson AL, Courtenay O, Kelly-Hope LA, et al. The importance of vector control for the control and elimination of vector-borne diseases. *PLoS Negl Trop Dis* 2020;14:e0007831. doi:10.1371/journal.pntd.0007831

24 Gunn JKL, Ernst KC, Center KE, et al. Current strategies and successes in engaging women in vector control: A systematic review. *BMJ Glob Heal* 2018;3:1–10. doi:10.1136/bmjgh-2017-000366

25 Mendoza C, Jaramillo G-I, Ant TH, et al. An investigation into the knowledge, perceptions and role of personal protective technologies in Zika prevention in Colombia. *PLoS Negl Trop Dis* 2020;14:e0007970. doi:10.1371/journal.pntd.0007970

26 The Global vector control response 2017–2030 (GVCR). Geneva: World Health Organization 2017. www.who.int/vector-control/publications/global-control-response/en/

27 Estimates of Resident Population in Brazilian Municipalities on July 1, 2020. Rio de Janeiro: Brazilian Institue of Geography and Statistics (IBGE) 2020. https://ftp.ibge.gov.br/Estimativas_de_Populacao/Estimativas_2020/estimativa_dou_2020.pdf (accessed 2 Dec 2020).

28 Sanchez Clemente N, Rodrigues M, Pascalicchio AP, *et al.* Cohort profile: The Jundiaí Zika cohort (JZC), a pregnancy and birth cohort in São Paulo state, Brazil. *BMJ Open* 2019;9:e027947. doi:10.1136/bmjopen-2018-027947

29 Netto EM, Moreira-soto A, Pedroso C, et al. High Zika Virus Seroprevalence in Salvador, Northeastern Brazil Limits the Potential for Further Outbreaks. *Am Soc Microbiol* 2017;8:1–14. doi:10.1128/mBio.01390-17

For peer review only - http://bmjopen.bmj.com/site/about/guidelines.xhtml

30 Clemente NS, Rodrigues M, Pascalicchio AP, et al. Cohort profile: the Jundiaí Zika cohort (JZC), a pregnancy and birth cohort in São Paulo state, Brazil. *BMJ Open* 2019;9:e027947--.http://researchonline.lshtm.ac.uk/id/eprint/4654300/

31 Braun, V. & Clarke V. Using Thematic Analysis in Psychology. *Qual Res Psychol* 2006;3:77–101. doi:10.1191/1478088706qp063oa

32 Krueger RA, Casey MA. Focus groups: A practical guide for applied research. 3rd ed. SAGE 2000.

33 Champion VL, Skinner CS. The Health Belief Model. In: Glanz, K., Rimer, B.K. & Viswanath, K., eds. Health Behavior and Health Education: Theory, Research, and Practice. San Francisco: Jossey-Bass 2008:45–66

34 Rosenstock IM, Strecher VJ, Becker MH. Social Learning Theory and the Health Belief Model. *Heal Educ Behav* 1988;15:175–83. doi:10.1177/109019818801500203

35 Phuanukoonnon S, Brough M, Bryan JH. Folk knowledge about dengue mosquitoes and contributions of health belief model in dengue control promotion in Northeast Thailand. *Acta Trop* 2006;99:6–14. doi:10.1016/j.actatropica.2006.05.012

36 Wong LP, AbuBakar S. Health Beliefs and Practices Related to Dengue Fever: A Focus Group Study. *PLoS Negl Trop Dis* 2013;7:2310. doi:10.1371/journal.pntd.0002310

37 Tong A, Sainsbury P, Craig J. Consolidated criteria for reporting qualitative research (COREQ): A 32-item checklist for interviews and focus groups. *Int J Qual Heal Care* 2007;19:349–57. doi:10.1093/intqhc/mzm042

38 Abraham C, Sheeran P. The Health Belief Model. In: Conner M, Norman P, eds. Predicting Health Behaviour: Research and Practice with Social Cognition Models. Maidenhead, Berkshire: Open University Press, McGraw Hill Education 2005:28–81

39 Cislaghi B, Heise L. Theory and practice of social norms interventions: Eight common pitfalls. *Global Health* 2018;14:1–10. doi:10.1186/s12992-018-0398-x

40 Peiter PC, Pereira R dos S, Moreira MCN, et al. Zika epidemic and microcephaly in Brazil: Challenges for access to health care and promotion in three epidemic areas. *PLoS One* 2020;15:e0235010. doi:10.1371/journal.pone.0235010

41 Barreto FR, Teixeira MG, Costa MDCN, et al. Spread pattern of the first dengue epidemic in the city of Salvador, Brazil. *BMC Public Health* 2008;8:51. doi:10.1186/1471-2458-8-51

42 Paixão ES, Teixeira MG, Rodrigues LC. Zika, chikungunya and dengue: The causes and threats of new and reemerging arboviral diseases. *BMJ Glob Heal* 2018;3:e000530. doi:10.1136/bmjgh-2017-000530

43 Callender DM. Factors contributing to and strategies to combat emerging arboviruses. *Glob Public Health* 2018;13:1846–52. doi:10.1080/17441692.2018.1464588

44 Khandia R, Munjal A, Dhama K, et al. Modulation of Dengue/Zika Virus pathogenicity by antibody-dependent enhancement and strategies to protect against enhancement in Zika Virus infection. *Front. Immunol.* 2018;9:597. doi:10.3389/fimmu.2018.00597

45 Gordon A, Gresh L, Ojeda S, et al. Prior dengue virus infection and risk of Zika: A pediatric cohort in

For peer review only

For peer review only - http://bmjopen.bmj.com/site/about/guidelines.xhtml

Nicaragua. *PLoS Med* 2019;16:e1002726. doi:10.1371/journal.pmed.1002726

46 Serrano-Collazo C, Pérez-Guzmán EX, Pantoja P, et al. Effective control of early zika virus replication by dengue immunity is associated to the length of time between the 2 infections but not mediated by antibodies. *PLoS Negl Trop Dis* 2020;14:1–28. doi:10.1371/journal.pntd.0008285

47 Althaus CL, Low N. How Relevant Is Sexual Transmission of Zika Virus? *PLoS Med* 2016;13:e1002157. doi:10.1371/journal.pmed.1002157

48 Power GM, Francis SC, Sanchez Clemente N, et al. Examining the Association of Socioeconomic Position with Microcephaly and Delayed Childhood Neurodevelopment among Children with Prenatal Zika Virus Exposure. *Viruses* 2020;12. doi:10.3390/v12111342

49 Souza WV De, Albuquerque MDFPM De, Vazquez E, et al. Microcephaly epidemic related to the Zika virus and living conditions in Recife, Northeast Brazil. *BMC Public Health* 2018;18. doi:10.1186/s12889-018-5039-z

50 Prata ARS, Pedroso D, Menezes G, et al. Juridical perspectives of interruption of pregnancy with zika virus infection regarding medical emotional and social consequences. *J Hum Growth Dev* 2018;28:77–81. doi:10.7322/jhgd.143875

51 Albuquerque MSV, Lyra TM, Melo APL, et al. Access to healthcare for children with Congenital Zika Syndrome in Brazil: Perspectives of mothers and health professionals. *Health Policy Plan* 2019;34:499–507. doi:10.1093/heapol/czz059

52 Diniz D, Ali M, Ambrogi I, et al. Understanding sexual and reproductive health needs of young women living in Zika affected regions: A qualitative study in northeastern Brazil. *Reprod Health* 2020;17. doi:10.1186/s12978-020-0869-4

53 Campos TC de. Zika, public health, and the distraction of abortion. *Med Heal Care Philos* 2017;20:443–6. doi:10.1007/s11019-016-9739-9

54 Weldon CT, Riley-Powell AR, Aguerre IM, et al. 'Zika is everywhere': A qualitative exploration of knowledge, attitudes and practices towards Zika virus among women of reproductive age in Iquitos, Peru. *PLoS Negl Trop Dis* 2018;12:e0006708. doi:10.1371/journal.pntd.0006708

55 Borges ALV, Moreau C, Burke A, et al. Women's reproductive health knowledge, attitudes and practices in relation to the Zika virus outbreak in northeast Brazil. *PLoS One* 2018;13:e0190024. doi:10.1371/journal.pone.0190024

56 Linde-Arias AR, Roura M, Siqueira E. Solidarity, vulnerability and mistrust: How context, information and government affect the lives of women in times of Zika. *BMC Infect Dis* 2020;20:263. doi:10.1186/s12879-020-04987-8

57 Wenham C, Arevalo A, Coast E, et al. Zika, abortion and health emergencies: a review of contemporary debates. *Global Health* 2019;15:49. doi:10.1186/s12992-019-0489-3

58 Wylie BJ, Hauptman M, Woolf AD, et al. Insect Repellants during Pregnancy in the Era of the Zika Virus. *Obstet Gynecol* 2016;128:1111–5. doi:10.1097/AOG.0000000000001685

59 Dos Santos SL, Cabral AC dos SP, Augusto LG da S. Knowledge, attitude and practice on dengue, the vector and control in an urban community of the northeast region, Brazil. *Cienc e Saude Coletiva*

For peer review only

For peer review only - http://bmjopen.bmj.com/site/about/guidelines.xhtml

2011;16:1319–30. doi:10.1590/S1413-81232011000700066

60 Raude J, MCColl K, Flamand C, et al. Understanding health behaviour changes in response to outbreaks: Findings from a longitudinal study of a large epidemic of mosquito-borne disease. *Soc Sci Med* 2019;230:184–93. doi:10.1016/j.socscimed.2019.04.009

61 Anderson EJ, Ernst KC, Martins FF, et al. Women's health perceptions and beliefs related to Zika virus exposure during the 2016 outbreak in northern Brazil. *Am J Trop Med Hyg* 2020;102:629–33. doi:10.4269/ajtmh.19-0311

62 Freire P, Bergman Ramos M, Macedo D. Pedagogy of the Oppressed. New York: Continuum International 2005:29–33

63 Nading A, Lowe L. Social Justice as Epidemic Control: Two Latin American Case Studies. *Med Anthropol Cross Cult Stud Heal Illn* 2018;37:458–71. doi:10.1080/01459740.2018.1485021

64 Carvalho MS, Honorio NA, Garcia LMT, et al. Aedes aegypti control in urban areas: A systemic approach to a complex dynamic. *PLoS Negl Trop Dis* 2017;11:1–15. doi:10.1371/journal.pntd.0005632

65 Hone T, Rasella D, Barreto ML, et al. Association between expansion of primary healthcare and racial inequalities in mortality amenable to primary care in Brazil: A national longitudinal analysis. *PLoS Med* 2017;14:1–19. doi:10.1371/journal.pmed.1002306

66 Wadge H, Bhatti Y, Carter A, et al. Brazil's Family Health Strategy: Using Community Health Workers to Provide Primary Care. *Commonw Fund* 2016;40. https://www.commonwealthfund.org/publications/case-study/2016/dec/brazils-family-health-strategy-using-community-health-care-workers (accessed 5 Dec 2020).

67 McNaughton D. The Importance of Long-Term Social Research in Enabling Participation and Developing Engagement Strategies for New Dengue Control Technologies. *PLoS Negl Trop Dis* 2012;6:1785. doi:10.1371/journal.pntd.0001785

68 Lima EP, Goulart MOF, Rolim Neto ML. Meta-analysis of studies on chemical, physical and biological agents in the control of Aedes aegypti Infectious Disease epidemiology. *BMC Public Health* 2015;15:858. doi:10.1186/s12889-015-2199-y

69 Nowell B, Boyd NM. Sense of Community Responsibility in Community Collaboratives: Advancing a Theory of Community as Resource and Responsibility. *Am J Community Psychol* 2014;54:229–42. doi:10.1007/s10464-014-9667-x

70 Atif M, Azeem M, Sarwar MR, et al. Congenital Chikungunya Virus Infection after an Outbreak in Salvador, Bahia, Brazil. *PLoS One* 2018;14:325–30. doi:10.1055/s-0036-1587323

71 Takemoto MLS, Menezes M de O, Andreucci CB, et al. The tragedy of COVID-19 in Brazil: 124 maternal deaths and counting. *Int J Gynecol Obstet* 2020;151:154–6. doi:10.1002/ijgo.13300

For peer review only - http://bmjopen.bmj.com/site/about/guidelines.xhtml

Figure 1: The Health Belief model

Adapted from Rosenstock et al. (1988),[34] the HBM predicts the decision making process to engage in a new health seeking behaviour. The individual assesses a perceived threat, potential barriers, benefits and their ability to undergo a behaviour change in response to knowledge, internal or external cues to action.[38]

Figure 2: Concept map of key, major and minor themes for community knowledge, attitudes and perceptions of Zika virus and vector-control in Salvador and Jundiaí, Brazil. Four key themes were mapped to determine their distance in relation to one another and whether they credibly fit constructs in the Health Belief Model.

For peer review only

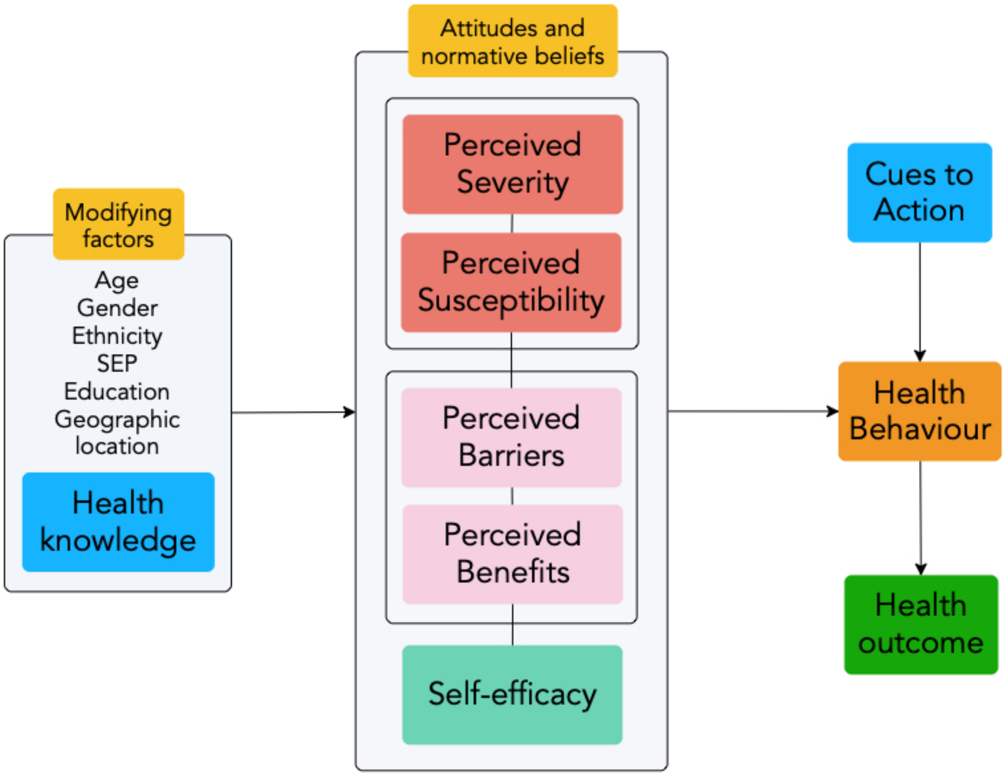

63x49mm (300 x 300 DPI)

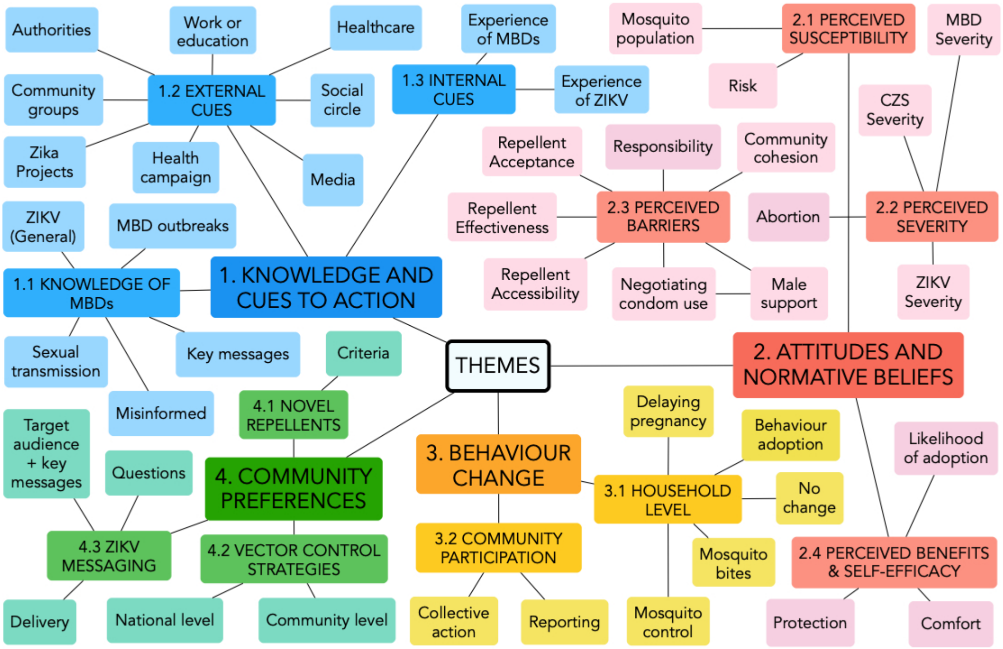

391x252mm (72 x 72 DPI)

Supplementary File 1: Topic Guide

"Post-epidemic community knowledge, attitudes and perceptions of Zika virus and vector-control strategies in Brazil"

Salvador and Jundiaí male semi-structured interviews or female focus-group discussions.

SOCIODEMOGRAPHIC SURVEY:

Socio-demographic data collected during recruitment.
Age: 18–30 [ ] 31–49 [ ] **Socioeconomic status:** High [ ] Middle [ ] Low [ ]

Date of interview	
Location of interview	
Name of interviewer	
Name of observer/note taker	
Time interview started	
Time interview ended	

INTERVIEW OPEN

- **Introduction by interviewer to the study**
- **Review and signing of informed consent form**
- **Start recording**

[12 questions]

1. **Mosquito control by families and the community.**

   a. What do you do in your home to reduce the number of mosquitoes that exist in your region and the number of bites that you and your family receive?
   Prompts: environmental cleaning, repellents, long sleeves, screens, bed nets etc.
   b. Is there any kind of community effort to reduce mosquito outbreaks?
   Detail (investigate this aspect well).

2. **Mosquito control by local authorities**
   a. What mosquito control activities, if any, are undertaken by the local authorities in your community?

3. **Changes in mosquito control practices**
   Has there been a change in mosquito control practices in your community, and in your own personal protection, since the emergence of Zika? If so, please provide details.

4. **Preferred mosquito control activities**
   Zika mosquitoes bite during the day. Given that, what kind of mosquito control would you like to see? *Better detail on personal protection including clothing.*

5. **Personal protection interventions**
   a. What do you think of personal protection interventions / alternatives / practices such as mosquito repellent clothing?
   b. How likely are you to use these alternatives and what would be your considerations?
   Prompt for cost, safety, comfort, fashion, duration of effectiveness.

6. **Concern about mosquito-borne diseases**
   a. In relation to various issues that you and your family have to manage on a daily basis, how much are you concerned about diseases transmitted by mosquitoes, such as dengue, chikungunya, yellow fever and Zika?

For peer review only

b. Are these four diseases of equal concern to you, or is one of more concern than the other three? *Detail.*

7. Knowledge about Zika

a. Turning specifically to Zika, do you know anyone personally who has had Zika?
b. If so, what is your relationship with this person / people?
c. What do you know about Zika?
d. Are there any aspects of the disease you would like to know more about?

8. Sources of knowledge about Zika

a. Where did you receive your knowledge about Zika? *(Prompt to include social media)*
b. Which of the Zika information sources do you think was the best, and which have been the least useful?

9. Messages from Zika

a. What are the main messages about Zika that you received from the authorities?
Poll for mosquito control, bite reduction and pregnancy issues.
b. Were these messages useful for you, or not? Explain.
Prompt for understanding, action, relevance, communication channel and key messages.

10. Postponement of pregnancy

a. Do you know women in your community who wanted to postpone pregnancy as a means of avoiding a baby with microcephaly?
b. Has this issue been a matter of concern or discussion in your community?

11. Sexual transmission of Zika

a. The Zika virus can be sexually transmitted to women by infected men. Do you think that the men in your community would be willing to practice safe sex in the recommended six months if their partner was pregnant, and they knew they were infected with Zika?
Prompt for condom use, sex without penetration etc.
b. Can you think of any messages that could be used to encourage men recently infected with Zika to practice safer sex? [MEN ONLY]
c. The Zika virus can be sexually transmitted to women by infected men. Would you be willing to use a condom for the recommended six months if your partner was pregnant and knew you were infected with Zika?

12. Abortion

a. Are you aware of cases in your community of pregnant women who have sought abortions because they feared they were carrying a baby with microcephaly?
b. If so, what did people say about it?
c. Do people in your community agree that a woman should have the right to terminate the pregnancy in these circumstances, or not? Or do they think she should carry the baby to term even if the baby may have microcephaly?

CLOSURE

- **Provide an opportunity for participants to discuss and ask questions about anything about Zika that they are in doubt about.**
- **Provide an official Zika information leaflet from the Ministry of Health website.**
- **Final question:** ***Would you be willing to attend a meeting to discuss the results of our study in 2 or 3 months? If so, please provide us with your contact details so that we can contact you.***
- **Thank all participants for their involvement and valuable responses.**

For peer review only

Supplementary File 2: Consolidated criteria for reporting qualitative research (COREQ): a 32-item checklist[1]

"Post-epidemic community knowledge, attitudes and perceptions of Zika virus and vector-control strategies in Brazil"

1. Tong A, Sainsbury P, Craig J. Consolidated criteria for reporting qualitative research (COREQ): a 32-item checklist for interviews and focus groups. *International Journal for Quality in Health Care* 2007;19(6):349–357. doi:10.1093/intqhc/mzm042

**Domain 1:** Research Team and Reflexivity		Response	
**1.**	**Interviewer/ facilitator**<br>Which author/s conducted the interview or focus group?	Yes	**Salvador:** Jorge Iriart (Coordinator), Vera Lucia Zaher-Rutherford, Tania Boccia, Mônica Manir.<br>**Jundiaí:** Eduardo Massad (Coordinator), Ana Maria Rico, Greice Bezerra Viana, Fernanda Macedo da Silva Lima.
**2.**	**Credentials**<br>What were the researcher's credentials?	Yes	**Grace Power:** Project Manager at the Global Vector Hub, London School of Hygiene & Tropical Medicine (LSHTM), UK.<br>**Dani Bancroft:** MSc student, Department of Public Health, Environments and Society, Faculty of Public Health and Policy, LSHTM, UK.<br>**John Kinsman:** Associate Professor, Department of Epidemiology and Global Health, Faculty of Medicine, Umeå University, Sweden.<br>**Robert Jones:** Research Fellow in Department of Disease Control, Faculty of Infectious and Tropical Diseases, LSHTM, UK.<br>**James Logan**: Head of Department of Disease Control, Faculty of Infectious and Tropical Diseases, LSHTM, UK.<br>**Jorge Iriat:** Associate Professor, Institute of Collective Health (ISC), Federal University of Bahia, Brazil.<br>**Eduardo Massad:** Professor, School of Medicine, University of São Paulo and Fundacao Getulio Vargas, Brazil.<br>**Raman Preet:** Research Coordinator, Department of Epidemiology and Global Health, Faculty of Medicine, Umeå University, Sweden.<br>**Interview facilitators:** MDs, nurses, pscyhologists and sociologists.
**3.**	**Occupation**<br>What was their occupation at the time of the study?	Yes	
**4.**	**Gender**<br>Was the researcher male or female?	Yes	

For peer review only

			Both Salvador and Jundiaí interview teams consisted of one male coordinator and three female interview facilitators.
**5.**	**Experience and Training**<br>What experience or training did the researcher have?	Yes	The principal investigators in Salvador and Jundiaí are native Brazilian Portuguese speakers familiar with the context of Zika virus in Bahia and Sao Paulo. The ZikaPLAN team carried out training and pilot testing of instrument with LSHTM visiting researchers.
**6.**	**Relationship**<br>Was a relationship established prior to study commencement?	No	No prior relationship was established.
**7.**	**Participant knowledge**<br>What did the participants know about the researcher? e.g. personal goals, reasons for doing the research	Yes	There were no direct benefits to participating in the study. Participants were provided information on the study objectives and relevance of the research, and a leaflet on Zika virus published by the Brazilian Ministry of Health at the end of the study.
**8.**	**Interviewer**<br>What characteristics were reported about the interviewer/facilitator?	No	

**Domain 2:** Study design		Response	
**9.**	**Methodological orientation and Theory**<br>What methodological orientation was stated to underpin the study? e.g. content analysis.	Yes	Thematic analysis guided by Braun and Clarke (2006).
**10.**	**Sampling**<br>How were participants selected?<br>e.g. purposive, convenience, consecutive.	Yes	Purposive sample for women of reproductive age (18–49). Not all men recruited into the study were the intimate partners of female participants.
**11.**	**Method of approach**<br>How were participants approached?<br>e.g. face-to-face, telephone, email.	No	Face-to-face recruitment at outpatient clinics, NGO settings and through researcher networks in the community.
**12.**	**Sample size**<br>How many participants were in the study?	Yes	A total of 120 participants: 103 women in focus groups (60 in Jundiaí and 43 in Salvador) and 17 men in semi-structured interviews (9 in Jundiaí and 8 in Salvador).

For peer review only

13.	**Non-participation**<br>How many people refused to participate or dropped out? Reasons?	No	Original study proposed 6–8 women per focus group. Salvador groups ranged from 4–7. For Jundiaí size of groups was not provided for secondary data analysis.
14.	**Setting of data collection**<br>Where was the data collected?<br>e.g. home, clinic, workplace	Yes	In Salvador, interviews were conducted in outpatient clinic rooms (FGDs) and at private residences (for male partners). FGDs in Jundiaí were conducted at an NGO-run community centre and in University Hospital rooms and faculty buildings.
15.	**Presence of non-participants**<br>Was anyone else present besides the participants and researchers?	Yes	LSHTM observers. No non-ZikaPLAN staff present.
16.	**Description of sample**<br>What are the important characteristics of the sample? e.g. demographic data, date	Yes	Interviews took place between April and May 2017. Sociodemographic data was collected but partially blinded for data analysis. Stratified age groups were provided for the majority of FGDs but not male participants.
17.	**Interview guide**<br>Were questions, prompts, guides provided by the authors? Was it pilot tested?	Yes	The topic, which includes questions, prompts and the sociodemographic data collected is provided as Supplementary File 1. This was pilot tested during training of interview facilitators with LSHTM research team present.
18.	**Repeat interviews**<br>Were repeat interviews carried out? If yes, how many?	No	No follow up interviews were carried out.
19.	**Audiovisual**<br>Did the research use audio or visual recording to collect the data?	Yes	The source data was audio recordings that was transcribed into Brazilian Portuguese by the Brazil ZikaPLAN team. This was then translated into English, with excerpts of transcripts verified for accuracy and credibility by the University College London Digital Meedia translation service. The source data was not shared for secondary data analysis.
20.	**Field notes**<br>Were field notes made during and/or after the interview or focus group?	Yes	ZikaPLAN observers and facilitators took field notes during the sessions.
21.	**Duration**<br>What was the duration of the interviews or focus group?	Yes	Each interview was arranged to last 60–90 minutes.Time stamps for interviews were not shared for analysis, but the wordcount of each transcript was presented in Table 1.
22.	**Data saturation**<br>Was data saturation discussed?	Yes	Yes, regarding participant responses to Question 5 in the topic guide on novel repellents (personal protective technologies).

For peer review only

**23.**	**Member checking**<br>Were transcripts returned to participants for comment and/or correction?	No	No.

	**Domain 3:** Analysis and Findings	Response	
**24.**	**Coders**<br>How many data coders coded the data?	Yes	One researcher for initial coding and three authors of one full FGD transcript. The principal investigators in Brazil carried out an initial analysis of transcripts following data collection. The data was then passed on to LSHTM for independent data analysis. The initial coding framework was presented to the principal investigators in Brazil for confirmability and triangulation purposes prior to theme generation.
**25**	**Coding tree**<br>Did authors provide a description of the coding tree?	Yes	The full codebook is provided in Supplementary File 3. A summary table of the key and major themes and a concept map of minor themes are provided in the manuscript.
**26**	**Derivation of themes**<br>Were themes identified in advance or derived from the data?	Yes	Coding was derived from the data. Theme generation was mostly inductive, with some deductive elements from grouping of codes together as responses to a certain question in the topic guide. Major themes were later mapped against constructs in a pre-defined conceptual framework for behaviour change for a potential fit (Rosenstock's Health Belief Model).
**27.**	**Software**<br>What software, if applicable, was used to manage the data?	Yes	Microsoft Excel was used to record sociodemographic data for each interview and observations, as well as administrative data, such as length, date and file names for the Brazilian and English transcripts to serve as an audit trail. NVivo 12 (QSR International, 2012) was used for coding and producing Figures 1 and 2.
**28.**	**Participant checking**<br>Did participants provide feedback on the findings?	No	Although considered, participant checking was not possible for this study. At the end of each interview participants were invited to consent for their contact information to be collected to disseminate the research findings.
**29.**	**Quotations presented**<br>Were participant quotations presented to illustrate the findings? Was each quotation identified?	Yes	Quotations in the manuscript were identified by focus group or interview site and number (unit of analysis), with the corresponding age group (18–30 or 31–49) in Table 1.
**30.**	**Consistency**<br>Was there consistency between the data presented and the findings?	Yes	–

For peer review only

**31.**	**Clarity of major themes**<br>Were major themes clearly presented in the findings?	Yes	A concept maps for themes was produced and this was used to navigate description of findings in relation to one another.
**32.**	**Clarity of minor themes**<br>Is there a description of diverse cases or discussion of minor themes?	Yes	Key and major themes are defined in Table 2 in the manuscript, and minor themes described in the findings. All themes are defined fully in the codebook (Supplementary File 3).

For peer review only

Supplementary File 3: Finalised Coding Framework (Codebook)

"Post-epidemic community knowledge, attitudes and perceptions of Zika virus and vector-control strategies in Brazil."

KEY THEMES [5] | **Major themes** [15] | Minor themes | *Child codes*

CODE	DESCRIPTION
**1. KNOWLEDGE**	Knowledge of MBD and ZIKV at the time of the study.
**1.1 Knowledge of MBDs**	Depth of understanding of ZIKV/MBDs, vector-control and misinformation.
Key messages	Reponses to Question 9 in the topic guide: "What are the main messages about Zika that you received from the authorities?" (Poll for mosquito control, bite reduction and pregnancy issues).
MBD knowledge	General knowledge on other mosquito-borne arboviruses: yellow fever, chikungunya, dengue fever. For example, references to outbreaks and epidemics, changes in prevalence/incidence, pathophysiology and vaccination campaigns.<br>Excluded: comments where ZIKV is the focus (coded as 'ZIKV General'), unless being compared to other MBDs.
Misinformed	Comments made by participants that may indicate misinformation or uncertainty around key messages related to MBDs.
Sexual transmission	Knowledge related to sexual transmission of ZIKV of both the participant and others in their social circle.<br>Excluded: content of messaging related to sexual transmission (coded as 'Key messages').
ZIKV (General)	Other knowledge related to ZIKV that does not fall into codes sexual transmission, severity of ZIKV symptoms, perceived risk (susceptibility), or experience of ZIKV (internal cues to action).

CODE	DESCRIPTION
**2. CUES TO ACTION**	Recalled stimuli for a decision-making process that may lead to behaviour change at the time of the study.[32]
**2.1 External Cues**	Stimuli from members of participants social network, the media, healthcare providers, the workplace or other community groups that trigger a decision-making process to seek additional information, engage in vector control or mosquito-bite reduction strategies, or other health seeking behaviours.
Health campaign	Alerts, visits from health agents for risk communication, billboards, posters and pamphlets, or messaging in the media explicitly described by the participant as being official public health information.
Zika Projects	The *Zika Project*, official NGO or volunteer projects taking place in hospitals (not always clear).<br>Excluded: activities identified as being conducted by local or national authorities (e.g. City Hall, Ministry of Health).
Healthcare	Accessing different forms of healthcare, such as maternity services, community clinics, dentists etc.<br>Excluded: experiences of having ZIKV or other MBDs, descriptions of symptoms of poor health (coded 'Other poor health').

For peer review only - http://bmjopen.bmj.com/site/about/guidelines.xhtml

Media	Parent code for references to media. Excluded: Official health campaign content (when clearly identified).
Broadcast media	Any media source that has been broadcast for entertainment purposes, such as television soaps and radio, or TV advertisements and print media, such as magazines and newspapers.
Online and social media	Casual or purposeful research online: accessing websites that may provide information about ZIKV. Messages and advertisements through social media, such as WhatsApp, Facebook, Instagram etc.
Social circle	Friends, neighbours, family members. Excluded: co-workers or acquaintances in formal settings (e.g. university, volunteer groups)
Work or education	Parent code for references to formal settings. Excluded: volunteering positions (e.g. in hospitals or ZIKV projects).
Higher Education	Participant is a current or former university/college student where ZIKV messaging has been delivered as part of a formal curriculum. Or there have been opportunities to access lectures and seminars on the epidemic.
Schools	Recalling experience of formal education for participants (e.g. high school). Or messages that children in the participants social circle have passed on to the participant informally.
Workplace	Participant either works in healthcare, formal education (teachers) or other profession where Zika messaging has been delivered at their workplace (e.g. works for the City Hall).
Community groups	Observing preventive activities or other stimuli in the community: informal groups (e.g. women's groups, gangs), community volunteer groups, gangs, centres of worship, neighbourhood associations, sports teams (e.g. capoeira, football) etc.
Government	National, state and municipal levels of government responsible for defining activities and protocols for *Aedes* interventions, including *"budget, personnel, technical guidelines, approved substances, routines, evaluation, and relationships with other sectors, such as education and public health"*.[4]
Local authorities	Aldermen, City Hall urban planning including waste management services. Health agents from the City Hall.. Excluded: 'health agents' described as being from an NGO, Ministry of Health or other national body.
National authorities	References to the national government: politicians, deployment of the army, legislation and policy makers, the Ministry of Health (e.g. official surveillance staff from the Brazilian MoPH) or other national bodies.
No action	No vector-control strategies are recalled to have taken place in the community, except for examples of vector control activities that have taken place more than one year prior to the start of epidemic in 2015.

For peer review only

**2.2 Internal cues**	Personal or secondary experience of confirmed/suspected cases of MBDs provide a stimulus for a decision-making process that leads to health seeking behaviour.
Experience of other MBDs	Confirmed or suspected cases of non-ZIKV mosquito-borne arboviruses by the participant or in the participant's social network.
Experience of Zika	Confirmed or suspected cases of ZIKV infection of the participant or in the participants social network.
Other poor health	Discussion of poor health that might be: non-communicable (e.g. disability or chronic conditions); related to non-ZIKV pregnancy complications; infectious diseases such as measles and H1N1 viruses; and other vector borne diseases such as Leptospirosis, tick borne diseases, Chaga's disease etc. Excluded: MBDs.

**3. ATTITUDES & NORMATIVE BELIEFS**	Personal attitudes are internal assessments of knowledge and cues to action for MBD preventive behaviours. Normative beliefs may inform personal attitudes according to how others perceive the behaviour in a social setting, such as the community.[1]
**3.1 Perceived Barriers**	An individual's assessment of the obstacles to ZIKV preventive behaviours, including condom use to prevent sexual transmission, mosquito bite-reduction and vector control strategies.
Abortion in the community	Awareness of individuals in the community that have terminated a pregnancy due to ZIKV or has undergone an abortion themselves as a result of concern of giving carrying a microcephaly child. Also includes community perspectives on the acceptability of abortion. Excluded: rights to abort and legislation.
Abortion rights	Participant responses to Question 12 in the topic guide: "Do people in your community agree that a woman should have the right to terminate pregnancy in these circumstances? Or do you understand that she should carry the pregnancy through to the end even if the baby has microcephaly?"
Depends on circumstances	More consideration around abortion. Comments that it is both acceptable and unacceptable, with examples of scenarios where abortion may be necessary or comments such as 'it's difficult' or 'it's complicated'. Includes discussion of financial circumstances and male partner support to evidence reasoning (only in reference to abortion). Excluded: caring for a child with CZS.
Opposed to abortion	Explicit opposition to the rights to abort. May cite religious grounds and morality e.g. perceptions of foetal viability and human rights. Normative beliefs around responsibility of pregnant mothers and their male partners. Unspecified negative responses, or strong opposed even when prompted by thee interviewer about microcephaly.
Supports rights to abort	Explicit support for the right to choose abortion. May express the need for legislative change, or cite perceptions of women's rights and autonomy regarding reproductive health.
Unclear response to abortion	Conflicted, contradictory or unintelligible response. May indicate discomfort expressing personal attitudes that conflict with the majority position.

For peer review only - http://bmjopen.bmj.com/site/about/guidelines.xhtml

Acceptance of novel repellents	Parent code for likelihood of community acceptance of novel repellents adoption (response to question 5 of the topic guide).
Appearance response	Aesthetic criteria related to the perception of wearing novel novel repellents in the community (e.g. smell, fashion).
Comfort response	Negative responses related to comfort of repellent clothing such as overheating, restricting physical movement and allergies or discomfort caused by repellent products.
Effectiveness of novel repellents	Responses related to perceived effectiveness of novel repellents for mosquito bite reduction. Scepticism or expression of interest may be contingent on how effective novel repellents are in practice (response to question 5 of the topic guide).
Accessibility of novel repellents	Parent code for perceptions of the ability to access novel novel repellents (response to question 5 of the topic guide).
Affordability response	Comments related to cost of novel novel repellents being a barrier to their adoption.
Availability response	Comments related to local availability of repellent tools for purchase, such references to vendor stock outs and likelihood of vendors in their community to sell novel repellent tools like clothing. Also included are comments around provision of novel repellents as gifts-in-kind from NGOs or the local or national authorities (e.g. through Bolsa Familia).
Awareness response	Participants awareness of novel repellent tools for personal protection. Comments about being unaware or vague.
Community cohesion	Social cohesion is defined as the *"extent of connectedness and solidarity among groups within society"*,[3] such as support from the community for vector-control or being able to seek social support when unwell. Comments about absent or poor relationships with neighbours, or not allowing unsolicited calls to household due to concerns about neighbourhood violence
Responsibility	Observation about participants expressing frustration over current preventive practices or ZIKV messaging, or being unable to negotiate shared responsibility for communal spaces for vector control. Blame of third parties or authorities.
Internal Responsibility	Expressing perceived locus of control for behaviour change lies with individual.
External Responsibility	Expressing that the perceived locus of control in relation to behaviour change around ZIKV and messaging as lying further upstream, such as with authorities (local, national).
Male support	Perceptions of male partners and the level of support participants feel they have from partners for ZIKV prevention. Perceptions of other male members of participants social circles, including family members, including normative beliefs related to gender (e.g. machísimo). Excluded: references to condom negotiation.

For peer review only - http://bmjopen.bmj.com/site/about/guidelines.xhtml

Negotiating condom use	Responses to question 11 of topic guide: "Do you think that the men in your community would be willing to practice safe sex (condom use, sex without penetration)? Do you think that if a man knew he was infected he would use a condom for six months?"
**3.2 Perceived Benefits**	*"Perceived benefits refer to an individual's assessment of the value or efficacy of engaging in a health-promoting behaviour to decrease risk of disease.*"[2]
Comfort of novel repellents	Positive perceptions of novel repellents use such as avoiding discomfort from bed nets, overheating from having to close windows and doors at night time, 'stickiness' or dislike of wearing topical repellents and allergic reactions (if referring to clothing).
Protection of novel repellents	Responses to question 5 of the topic guide related to enhanced protection of themselves or others in their social network from MBD infection. e.g. during pregnancy, family members such as children or the elderly.
**3.3 Perceived Severity**	A subjective assessment of the severity of ZIKV and potential consequences of infection or a CZS pregnancy.<br>*"The combination of perceived severity and perceived susceptibility is referred to as perceived threat"*.[2]
CZS caregiving	Experience of caring for a child with microcephaly in the in the participants social network.<br>Perceptions of the severity of microcephaly in the community, e.g. the burden of care giving for a child with microcephaly (the financial or social implications). Excluded: comments around male support to care for a child with CZS.
Other MBD Severity	Perceptions related to the severity of symptoms of other MBDs. Comments about concern or fear related to other MBDs.<br>Excluded: Perceptions of poor health due to non mosquito-borne arboviruses.
ZIKV Severity	Perceptions related to the severity of symptoms of ZIKV. Comments about concern or even fear related to ZIKV.<br>Excluded: comments about CZS caregiving.
**3.4 Perceived Susceptibility**	A subjective assessment of risk of ZIKV infection or a CZS pregnancy. Combines with perceived severity for perceived threat.[2]
Mosquito population	Comments on the burden of the mosquito population in a specific geographical area, mosquito physiology and behaviour. Other observations made by the participant or members of the participants social circle on the activity of mosquitoes in that area.
Risk response	Perceived risk of ZIKV transmission and CZS. For example: the periodomicile does not have a large mosquito population; the participant is not pregnant or has undergone the menopause; perceptions that the risk of contracting ZIKV to be very low.<br>(Also includes responses to question 5 of the topic guide).

**3.5 Self Efficacy**	An individual's perception of their competence to successfully undergo a behaviour change.[2]
Likelihood of adoption	Willingness or likelihood to adopt novel repellents. Describes being motivated or unmotivated to take responsibility for household level behaviours or community participation to reduce transmission of ZIKV. Excluded: change in behaviour that has happened.
Negative response	Unspecified negative response to Q5 of the topic guide indicating disinterest or not willing to adopt novel repellents.
Positive response	Unspecified positive response to question 5 of the topic guide indicating willingness or interest to adopt novel repellents.

**4. BEHAVIOUR CHANGE**	Behaviour changes attributed to the ZIKV epidemic, existing MBD preventive behaviours or no changes to mosquito population control or bite-reduction strategies, including use of novel repellent tools.
**4.1 Community Participation**	Participant has engaged with others in the community, describing activities for collective action for vector control since the arrival of the ZIKV epidemic.
Collective Action	Engaging with others for activities specific to vector-control, e.g. consulting with neighbours or community groups, exchanging advice with members of their immediate social circle.
Reporting	Reporting of sources of concern for mosquito control (e.g. communal spaces and garbage, larval growth) to landlords or building maintenance staff, local authorities, health agents or other third parties in position of power.
**4.2 Household Level**	Practices to prevent mosquito breeding sites, mosquito-bite reduction and mosquito entry to the household.
Mosquito bites	Preventive practices taken personally to reduce risk of mosquito bites.
Avoidance behaviour	Avoiding certain times of day or areas known to have more mosquitoes. Closing of windows or doors to prevent mosquito entry.
Bed nets, screens	Insecticide treated or untreated mosquito bed nets, window or door screens to prevent mosquito entry.
Electronic devices	Plug in mosquito repellent devices, air conditioning and fans, electric 'racket' killing devices, sonic devices.
Long clothing	Covering up with long sleeves or legs to prevent exposed skin to mosquitoes.
Other topical emollients	Applying moisturiser, sun screen or other topical lotions that are not manufactured to function as mosquito repellents.
Repellents	Chemical or citronella repellents, room sprays or alternative methods like burning coils, egg shells, cardboard etc. Excluded: electronic plug-in repellents or sonic devices.

For peer review only - http://bmjopen.bmj.com/site/about/guidelines.xhtml

Supplements	Participants describe taking oral supplements due to belief this will reduce likelihood of mosquito bites (e.g. vitamin B complex).
Mosquito population control	Parent code for preventive practices related to vector-control in the household.
Animals	Wild dogs, pets or other non-arthropod animals. Coded for potential implications for One Health.
Garbage disposal	Further detail relating to garbage collection or recycling to prevent water accumulation.
Hygiene	Using soap, scrubbing surfaces, applying disinfectant, sweeping and references to hygiene and cleanliness.
Insecticide	Water treatments to stop larval growth cycle (larvicides), or spraying chemical insecticides indoors or around the periodomicile.
Stagnant water	Practices to prevent pooling of clean or stagnant water in the periodomicile: filling plant pots or receptacles with sand; removing rubble; turning over pots and drinks bottles; wiping condensation down from surfaces, or other measures to encourage drainage.
Behaviour adoption	Behaviour change attributed to ZIKV; including comments on increased or decreased frequency of an activity.
Delaying pregnancy	Decision to prevent or delay pregnancy, detailing methods that include use of contraceptives, non-penetrative sex, abstinence etc. Also referrals to members of the social circle or their wider network that delayed pregnancy. Excluded: abortion.
No change	Behaviours were practiced before ZIKV epidemic, or no adoption of preventive practices since the ZIKV epidemic.

**5. COMMUNITY PREFERENCES**	Expressed needs or elaboration of preferences for mosquito-abatement products, or coordination of vector-control strategies and health promotion related to ZIKV.
**5.1 Novel Repellents**	Novel topical mosquito repellents, repellent-impregnated clothing or other wearables (e.g. plastics) designed to repel and prevent mosquito bites.
Preferred criteria	Preferred criteria for novel repellents and repellent wearables that would encourage adoption, such as responses relating to comfort, appearance, affordability, effectiveness and other responses to question 5 of the topic guide.
Suggestions	Responses where participant mention a criterion for novel repellents not coded for in the other responses, e.g. suggestions for alternative repellent products (e.g. microencapsulated bracelets). Any other responses to question 5 of the topic guide.

For peer review only - http://bmjopen.bmj.com/site/about/guidelines.xhtml

**5.2 Vector-control**	Preferred activities for mosquito population control; perceptions of where the responsibility lies for vector-control.
Community Level	Suggestions for action related to community groups, local authorities or within their local social network. e.g. health inspections or appointment of community members for capacity building and mobilisation of funding.
National Level	Preferred activities at the national level. For example, suggestions for action related to government policy and legislation, funding, public health campaigns or vaccine research and development.
**5.3 ZIKV Messaging**	Preferred risk communication and community engagement for MBD surveillance, mosquito bite-reduction and vector control strategies. Responses to: "Which of the Zika information sources do you think was the best and which was the least useful?"
Preferred delivery	Preferred format, frequency and source of delivery of risk communication (e.g. social media, in person).
Preferred target audience and messaging	Preferred target for risk communication and community engagement where participants express there is the most need (e.g. men, school children) and preferred key messages or specific topics related to ZIKV and MBDs.
Questions	Expressing lack of understanding or requests for clarification on topics related to ZIKV or other MBDs.

**Discard pile**	Participant responses do not answer any of the topic guide questions or are useful to the research question to justify creation of a new code.

1. Cislaghi B, Heise L. Theory and practice of social norms interventions: Eight common pitfalls. *Global Health* 2018;14(1):1–10.
2. Stretcher V, Rosenstock IM. The Health Belief Model. In: Glanz K, Lewis, F.M., Rimer BK, editors. *Health behavior and health education: Theory, research, and practice* San Francisco: Jossey-Bass; 1997.
3. Manca AR. Social Cohesion. In: *Encyclopedia of Quality of Life and Well-Being Research* Dordrecht: Springer Netherlands; 2014 [cited 2020 Sep 23]. p. 6026–8.
4. Carvalho MS, Honorio NA, Garcia LMT, Carvalho LC de S. Aedes ægypti control in urban areas: A systemic approach to a complex dynamic. *PLoS Negl Trop Dis* 2017;11(7):1–15.

For peer review only - http://bmjopen.bmj.com/site/about/guidelines.xhtml

BMJ Open

Vector control strategies in Brazil: A qualitative investigation into community knowledge, attitudes and perceptions following the 2015–16 Zika virus epidemic

Journal:	*BMJ Open*
Manuscript ID	bmjopen-2021-050991.R1
Article Type:	Original research
Date Submitted by the Author:	21-Oct-2021
Complete List of Authors:	Bancroft, Dani; London School of Hygiene & Tropical Medicine, Department of Public Health<br>Power, Grace; London School of Hygiene & Tropical Medicine, Department of Disease Control; University of Bristol, MRC Integrative Epidemiology Unit, Bristol Medical School<br>Jones, Robert; London School of Hygiene & Tropical Medicine, Department of Disease Control<br>Massad, Eduardo; University of Sao Paulo, School of Medicine; Fundacao Getulio Vargas<br>Iriat, Jorge Bernstein; Federal University of Bahia, Institute of Collective Health<br>Preet, Raman; Umeå University, Department of Epidemiology and Global Health<br>Kinsman, J; Umea University, Department of Epidemiology and Global Health<br>Logan, James; London School of Hygiene & Tropical Medicine, Department of Disease Control
**Primary Subject Heading**:	Public health
Secondary Subject Heading:	Infectious diseases, Qualitative research, Global health, Health policy
Keywords:	Entomology < TROPICAL MEDICINE, Epidemiology < TROPICAL MEDICINE, Infection control < INFECTIOUS DISEASES, Public health < INFECTIOUS DISEASES

SCHOLARONE™
Manuscripts

I, the Submitting Author has the right to grant and does grant on behalf of all authors of the Work (as defined in the below author licence), an exclusive licence and/or a non-exclusive licence for contributions from authors who are: i) UK Crown employees; ii) where BMJ has agreed a CC-BY licence shall apply, and/or iii) in accordance with the terms applicable for US Federal Government officers or employees acting as part of their official duties; on a worldwide, perpetual, irrevocable, royalty-free basis to BMJ Publishing Group Ltd ("BMJ") its licensees and where the relevant Journal is co-owned by BMJ to the co-owners of the Journal, to publish the Work in this journal and any other BMJ products and to exploit all rights, as set out in our licence.

The Submitting Author accepts and understands that any supply made under these terms is made by BMJ to the Submitting Author unless you are acting as an employee on behalf of your employer or a postgraduate student of an affiliated institution which is paying any applicable article publishing charge ("APC") for Open Access articles. Where the Submitting Author wishes to make the Work available on an Open Access basis (and intends to pay the relevant APC), the terms of reuse of such Open Access shall be governed by a Creative Commons licence – details of these licences and which Creative Commons licence will apply to this Work are set out in our licence referred to above.

Other than as permitted in any relevant BMJ Author's Self Archiving Policies, I confirm this Work has not been accepted for publication elsewhere, is not being considered for publication elsewhere and does not duplicate material already published. I confirm all authors consent to publication of this Work and authorise the granting of this licence.

For peer review only

Title: Vector control strategies in Brazil: A qualitative investigation into community knowledge, attitudes and perceptions following the 2015–16 Zika virus epidemic

Version date: October 15, 2021

Manuscript word count: 5,718 (excluding tables and legends: 638).

Abstract word count: 300

References: 76 (including reference [39] to data repository)

Supplementary files: 1. Topic guide; 2. COREQ checklist; 3. Codebook.

Key words: Public health, Infection control, Epidemiology, Entomology

For peer review only

Dani Bancroft [1,†], Grace M. Power [2, 3, †], Robert T. Jones [2 *], Eduardo Massad [4], Jorge Bernstein Iriat [5], Raman Preet [6], John Kinsman [6], James G. Logan [2]

[1] Department of Public Health, Environments and Society, Faculty of Public Health and Policy, London School of Hygiene & Tropical Medicine, London, United Kingdom; [2] Department of Disease Control, Faculty of Infectious and Tropical Diseases, London School of Hygiene & Tropical Medicine, London, United Kingdom; [3] MRC Integrative Epidemiology Unit, Population Health Sciences, Bristol Medical School, University of Bristol, United Kingdom; [4] School of Medicine, University of São Paulo and Fundação Getulio Vargas, Brazil; [5] Institute of Collective Health, Federal University of Bahia, Brazil; [6] Department of Epidemiology and Global Health, Faculty of Medicine, Umeå University, Sweden.

† Joint first authors

* Corresponding author

Corresponding author:

Robert T. Jones – Robert.jones@lshtm.ac.uk

Department of Disease Control, Faculty of Infectious and Tropical Disease

London School of Hygiene & Tropical Medicine, Keppel Street, London, WC1E 7HT

United Kingdom

For peer review only - http://bmjopen.bmj.com/site/about/guidelines.xhtml

ABSTRACT

Objective:

The World Health Organization declared a Public Health Emergency of International Concern following the rapid emergence of neonatal microcephaly in Brazil during the 2015–2016 Zika virus (ZIKV) epidemic. In response, a national campaign sought to control *Aedes* mosquito populations and reduce ZIKV transmission. Achieving adherence to vector control or mosquito-bite reduction behaviours, including the use of topical mosquito-repellents, is challenging. Co-production of research at the community level is needed to understand and mitigate social determinants of lower engagement with *Aedes* preventive measures, particularly within disempowered groups.

Design:

In 2017, the Zika Preparedness Latin America Network (ZikaPLAN) conducted a qualitative study to understand individual and community level experiences of ZIKV and other mosquito-borne disease outbreaks. Presented here is a thematic analysis of 33 transcripts from community focus groups and semi-structured interviews, applying the Health Belief Model (HBM) to elaborate knowledge, attitudes and perceptions of ZIKV and vector control strategies.

Participants:

120 purposively sampled adults of approximate reproductive age (18–45); 103 women participated in focus groups and 17 men in semi-structured interviews.

Setting:

Two sociopolitically and epidemiologically distinct cities in Brazil: Jundiaí (57km north of São Paolo) and Salvador (Bahia state capital).

Results:

Four key and 12 major themes emerged from the analysis: (i) knowledge and cues to action; (ii) attitudes and normative beliefs (perceived threat, barriers, benefits and self-efficacy); (iii) behaviour change (household prevention and community participation); and (iv) community preferences for novel repellent tools, vector control strategies and ZIKV messaging.

Conclusions:

Common barriers to repellent adherence were accessibility, appearance and effectiveness. A strong case is made for the transferability of the HBM to inform epidemic preparedness for mosquito-borne disease outbreaks at the community level. Nationally, a health campaign targeting men is recommended, in addition to local mobilisation of funding to strengthen surveillance, risk communication and community engagement.

For peer review only

For peer review only - http://bmjopen.bmj.com/site/about/guidelines.xhtml

STRENGTHS AND LIMITATIONS OF THIS STUDY

- There are limited examples of direct post-epidemic engagement and research co-production with disempowered groups in Brazil, including pregnant women and communities with lower socioeconomic position.
- Focus groups and semi-structured interviews provided rich qualitative data on perceptions of vector control strategies and barriers to community engagement with preventive measures during the Zika epidemic.
- A large sample of community members of different ages from two geographically distinct cities in Brazil promoted generalisability of the study outcomes and recommendations.
- A limitation of the focus groups is that participants were asked about their awareness and interest in repellent clothing, and most were not familiar with these as options for personal protection.
- Since interviews took place in 2017, follow-up sessions may have strengthened understanding of how perceptions of *Aedes*-related diseases changed over time, particularly following subsequent outbreaks of chikungunya and yellow fever in Brazil.

For peer review only

For peer review only - http://bmjopen.bmj.com/site/about/guidelines.xhtml

BACKGROUND

Zika virus (ZIKV) is a flavivirus primarily transmitted by *Aedes aegypti*, an aggressive day-biting mosquito found in tropical and subtropical climates.[1] Secondary modes of transmission include sexual contact and blood transfusions, as well as vertical transmission in ZIKV-seropositive women.[2,3] Vertical transmission of ZIKV during pregnancy has been associated with devastating developmental consequences in infected offspring, including microcephaly and other neurological impairments that are collectively recognised as Congenital Zika Syndrome (CZS).[4–6]

On 12 November 2015, following a significant increase in the number of children born with microcephaly in Northeast Brazil, the Ministry of Health (MoH) declared ZIKV a national emergency.[7] Given the temporal and spatial overlap of microcephaly cases and ZIKV outbreaks, in February 2016, the World Health Organization (WHO) subsequently declared ZIKV a Public Health Emergency of International Concern.[8] By February 2017, Brazil accounted for more than half of the confirmed cases of ZIKV (n = 201,821) and 90% of cases of CZS (n = 2,366) in the Americas.[9]

Population control of *Ae. aegypti* is the main line of defence against ZIKV transmission.[10] In addition to natural reservoirs, rapid or unplanned urbanisation has contributed to the metropolitan success of this species, which breeds in areas with poor drainage, such as open drains, water tanks and receptacles created by household waste.[11] Negotiating responsibility in relation to maintenance of communal spaces (e.g. the individual, community, government or society more broadly) and failure to identify persistent *Ae. aegypti* or *Ae. albopictus* cryptic breeding sites hinders adequate vector control.[12] Chronic underfunding and intervention siloes also further undermine efforts to prevent mosquito-borne disease (MBD) outbreaks.[13]

Individual-level mosquito bite-reduction strategies include wearing long-sleeved clothing to create physical barriers, as well as applying topical mosquito repellents.[14] Non-topical strategies include fabric repellent or insecticide sprays.[14,15] However, many repellents do not provide long-lasting protection and often require re-application.[16] Integration of repellents or insecticides into wearable materials, a method used to treat military clothing in some settings,[17] may instead provide an effective and scalable prevention strategy that is of value to at-risk communities in Brazil.[18]

To reduce sexual transmission of ZIKV, Brazil's MoH promoted condom use and postponement of planned pregnancy during the epidemic.[19] Whilst international guidelines also advocated the relaxation of anti-abortion legislation, in Brazil, abortion is only decriminalised for foetal anencephaly (a lethal birth defect), rape or conditions that risk maternal death.[20,21] As a result, abortion was omitted from the MoH protocol on reproduction rights and pre-natal, delivery and postpartum care in response to ZIKV.[19] Instead, Brazil's policy strategy emphasised vector control, technology research and development, and assurance of access to health care for individuals with long-term sequelae of ZIKV infection.[22]

For peer review only - http://bmjopen.bmj.com/site/about/guidelines.xhtml

In November 2016, the WHO declared the end of the ZIKV epidemic.[23] However, as the epidemic waned, development of the most promising vaccine candidates faced challenges in clinical efficacy trials.[24] Since *Aedes* mosquitoes continue to transmit arboviruses worldwide, the epidemic preparedness community remains concerned about the high risk of future outbreaks of ZIKV and other emerging MBDs.[24–27] Brazil's limited success in controlling *Aedes* populations therefore indicates the importance of investigating the social determinants underlying the 2015–16 ZIKV epidemic.[22,26]

Successful uptake of mosquito-bite preventive strategies is contingent on the broader sociopolitical context, as behaviour change is strongly informed by family, community, cultural, political and economic factors.[13,26,28] The WHO Global Vector Control Response 2017–2030 outlined engagement and mobilisation of communities as one of its four pillars for effective, locally adapted and sustainable vector control.[26] Despite this, during the 2015–2016 ZIKV epidemic, few examples of direct post-epidemic engagement or research co-production with populations at highest-risk of adverse health outcomes following ZIKV infection were observed, including with pregnant women and communities experiencing lower socioeconomic position.[29,30] Funding allocated for social research was also markedly lower in comparison to other disciplines.[29] Therefore, to analyse community experiences of Zika virus and vector control strategies in a Brazilian context,[22,31] we consider the application of Rosenstock's Health Belief Model (HBM).[32,33] The HBM is a widely adopted theoretical framework for behaviour change that has been applied to other qualitative studies investigating MBDs.[34,35]

Aims

This study aims to identify determinants of low adherence to mosquito-bite preventive behaviours by applying the HBM as a conceptual model for community knowledge, attitudes and perceptions towards ZIKV and vector control strategies in two sociopolitically and epidemiologically distinct populations in Brazil: Jundiaí, a municipality of São Paulo (pop. 423,000) and Salvador, the state capital of Bahia (pop. 2.9 million).[36] To best contextualise these drivers, our additional study objectives were to: (1) elaborate household preferences for vector control strategies, particularly with regard to treated clothing; (2) identify perceived barriers to adoption of prevention behaviours; (3) contrast perceptions of ZIKV control with other mosquito-borne arboviruses; (4) compare normative beliefs of pregnancy postponement and abortion to reduce foetal susceptibility to CZS; and (5) map themes against a theoretical framework for behaviour change.

For peer review only - http://bmjopen.bmj.com/site/about/guidelines.xhtml

METHODS

Participant Recruitment and Data Collection

From March-August 2017, focus-group discussions (FGDs) with adult women of approximate reproductive age (18–49) and semi-structured interviews (SSIs) with male partners were conducted in Jundiaí and Salvador. Both cities have cohorts of children living with CZS.[37,38] The interview topic guide comprised of 12 questions covering three main areas of enquiry: (i) perceptions and practices of mosquito control, (ii) protecting oneself against mosquito bites, and (iii) knowledge and perceptions of ZIKV (**Supplementary File 1**).[39] All sessions were delivered in Brazilian Portuguese, and the source data transcribed and translated into English for analysis.

Participants

Participants were purposively sampled and consented to participate in the study. The pregnancy status of women was not taken into account and a sociodemographic survey stratified participants by age (18–30 or 31–49 years). In Jundiaí, recruitment took place in outpatient departments at University Hospital, and data collection in both faculty buildings and a non-government organisation (NGO) run community centre. In Salvador, recruitment and data collection took place in two Primary Care Units. In both cities, men were recruited through community stakeholders and interviewed at private residences.

Patient and Public Involvement

The principal investigators from Jundiaí and Salvador are native Brazilian speakers familiar with the study setting and context. To ensure the research question was informed by patients' priorities and experiences, it the topic guide was developed and pilot tested with research teams local to the study sites. Additionally, 17 in-depth interviews were also conducted with health professionals, including Salvador health professionals working in a Primary Care Unit and in private clinics, and community leaders, with three religious leaders from Kardecism, Candomblé (an Afro-Brazilian religion) and an evangelical Christian church. To disseminate results, those who expressed interest and provided consent were invited to attend a follow up session to discuss initial findings in September 2017.

Analysis

In total, 33 transcripts were analysed (**Table 1**). Open coding was performed in NVivo (version 12, QSR International). Theme generation followed Braun and Clarke's six phases for thematic analysis.[40] A preliminary coding framework was established from the topic guide. However, coding was mostly inductive, by grouping prevalent response patterns into higher-order categories.[41] Major themes were mapped against the constructs in the HBM (**Figure 1**).[32,33] A concept map for themes was developed to gauge whether there was a credible fit with the HBM (**Figure 2**). The 32-item Consolidated Criteria for Reporting Qualitative Research (COREQ) tool was used to ensure all key methodological issues were taken into account (**Supplementary File 2**).[42]

For peer review only

Table 1: Summary of interview transcripts provided for analysis

A total of 17 focus-group discussions (FGD) with 103 women and 16 semi-structured interviews (SSI) with 17 men were included in the analysis. Three FGDs and all semi-structured interviews were missing sociodemographic data (age). Jundiaí transcripts were missing the breakdown of participants by focus group. Jundiaí FGD-9 was selected for triangulation. Jundiaí SSI-9 was a deviant case excluded from the analysis.

Transcript		Words	Participants	Age	Transcript		Words	Participants	Age
**Female participants**			**60**					**43**	
1	Jundiaí-FGD-1	4,338		18–30	19	Salvador-FGD-1	14,762	6	31–49
2	Jundiaí-FGD-2	4,399		31–49	20	Salvador-FGD-2	3,318	6	18–30
3	Jundiaí-FGD-3	4,067		18–30	21	Salvador-FGD-3	16,863	5	31–49
4	Jundiaí-FGD-4	3,409		31–49	22	Salvador-FGD-4	10,262	4	18–30
5	Jundiaí-FGD-5	1,691			23	Salvador-FGD-5	8,103	5	18–30
6	Jundiaí-FGD-6	4,026		31–49	24	Salvador-FGD-6	15,619	5	31–49
7	Jundiaí-FGD-7	1,239			25	Salvador-FGD-7	13,138	6	31–49
8	Jundiaí-FGD-8	3,012		31–49	26	Salvador-FGD-8	9,256	7	18–30
**9**	**Jundiaí-FGD-9**	**1,860**							
**Male participants**			**9**					**8**	
10	Jundiaí-SSI-1	41	1		27	Salvador-SSI-1	619	1	
11	Jundiaí-SSI-2	44	1		28	Salvador-SSI-2	346	1	
12	Jundiaí-SSI-3	37	1		29	Salvador-SSI-3	208	1	
13	Jundiaí-SSI-4	65	1		30	Salvador-SSI-4	407	1	
14	Jundiaí-SSI-5	73	1		31	Salvador-SSI-5	269	1	
15	Jundiaí-SSI-6	147	1		32	Salvador-SSI-6	367	1	
16	Jundiaí-SSI-7	276	1		33	Salvador-SSI-7	298	1	
17	Jundiaí-SSI-8	105	1		34	Salvador-SSI-8	239	1	
**18**	**Jundiaí-SSI-9**	**4,312**	**1**	**18–30**					

For peer review only

RESULTS

A total of 120 individuals participated in the study: 103 women (60 in Jundiaí, 43 in Salvador); and 17 men. Responses to questions on novel repellents were initially coded: effectiveness; affordability; availability; appearance; comfort; protection; risk; and other. Each were mapped against the HBM as: risk (perceived susceptibility); positive responses such as protection (perceived benefits); willingness to adopt (self-efficacy); negative responses for effectiveness, acceptance or accessibility (perceived barriers); and alternative suggestions (preferred criteria). A finalised concept map comprised of 44 minor themes and 12 major themes grouped under four higher-order key themes (**Figure 2**; **Table 2**). Definitions are provided in the codebook (**Supplementary File 3**).

Table 2: Summary table of definitions for key and major themes.

Theme		Definition
**1.**	**Knowledge and Cues to Action**	Depth of understanding of ZIKV, MBDs, vector control and key messages identified by participants. Stimuli for a decision-making process that may have led to behaviour change, as recalled at the time of study.[32]
1.1	Knowledge of MBDs	Participant awareness of MBDs and ZIKV, as well as the community and national response to outbreaks at the time of the study.
1.2	External Cues to Action	External stimuli, such as a health campaign, triggered a decision-making process that may have led to a behaviour change.
1.3	Internal Cues to Action	Direct and indirect experiences of confirmed or suspected cases of MBDs triggered a decision-making process that may have led to a behaviour change.
**2.**	**Attitudes and Normative beliefs**	Personal attitudes are internal assessments of knowledge and cues to action for MBD preventive behaviours. Normative beliefs may inform personal attitudes according to how others perceive the behaviour in a social setting, such as the community.
2.1	Perceived Susceptibility	A subjective assessment of the risk of ZIKV infection or a CZS pregnancy and the first component of perceived threat.[32]
2.2	Perceived Severity	A subjective assessment of the severity of ZIKV symptoms and CZS and the second component of perceived threat.[32]
2.3	Perceived Barriers	An individual's assessment of the barriers to uptake of ZIKV preventive behaviours for sexual transmission, mosquito bite-reduction and vector control.
2.4	Perceived Benefits and Self-efficacy	An individual's perception of the benefits of novel repellent technologies and their ability to successfully undergo a behaviour change by adopting preventive strategies.
**3.**	**Behaviour Change**	Behaviours either attributed to the ZIKV epidemic, are pre-existing practices against MBDs (no change), or no preventive measures were taken.
**3.1**	Household level	Practices to prevent mosquitos from breeding and exposure to mosquito bites at the household level.
**3.2**	Community Participation	Engaging with others in the community; participants describe activities for collective action for vector control.
**4.**	**Community Preferences**	Expressed needs and preferences for mosquito bite-reduction strategies, coordination of vector control and ZIKV messaging, including questions.
4.1	Novel Repellents	Preferred criteria for novel topical mosquito repellents, repellent-impregnated clothing or other wearables designed to prevent mosquito bites.
4.2	Vector control strategy	Preferred activities for mosquito population control, including surveillance.
4.3	ZIKV messaging	Preferred content, source and format for delivery of ZIKV risk communication and community engagement.

For peer review only - http://bmjopen.bmj.com/site/about/guidelines.xhtml

Knowledge and Cues to Action

Participants expressed uncertainty around which vectors transmit ZIKV. In Salvador, several participants accurately described the appearance and behaviour of *Ae. aegypti*. However, the majority of participants did not differentiate the mosquito from other biting insects and some were misinformed. Dengue was the second most commonly identified MBD, although chikungunya and yellow fever were also discussed. Most participants were aware of the impact of ZIKV infection on pregnancy as a distinction from other infectious diseases. However, sexual transmission was poorly understood, and questions from women that disclosed higher levels of education often related to the pathophysiology of ZIKV and unknown sequalae.

> *[P1]: So, [microcephaly] sparked people's interest: "Pow, then really, that's the difference between Zika and dengue and H1N1 [influenza]."*
> **Salvador-FGD1**

> *[P1]: There are 3 different mosquitoes, right?*
> **Salvador-FGD2**

> *[P2]: [I understood that ZIKV is transmitted by] the host, yes. But not from person to person…This has not been clear to me until today.*
> **Salvador-FGD3**

Many women first learned about ZIKV and were advised to use condoms when accessing maternity services. Often exposure to public health information in broadcast or print media, including pamphlets and posters, was described. Several mentioned learning about ZIKV online, via social media, as well as in workplace or higher-education settings. Other external cues to action included direct contact with political representatives, NGOs or community volunteers involved with Zika projects. Health agents were described to inspect households and disseminate public health information about *Aedes* and preventive strategies. One key message recalled was to remove standing water from around the household and spaces shared with neighbours; participants from four FGDs also recalled a visit from military personnel to promote clearing of communal spaces.

> *There was a joint effort that the government [made] in the neighbourhood, like this...It was like D-Day against Zika, dengue…*
> **Salvador-FGD4**

Visibility of vehicle-mounted thermal spraying/fogging in previous years was recalled by several groups, although most activities were described as having ended. Most agreed that ZIKV messaging had slowed or stopped at the time of their interview, and several participants recalled no community vector control interventions occurring in their neighbourhood at all. Internal cues to action comprised direct or indirect

For peer review only - http://bmjopen.bmj.com/site/about/guidelines.xhtml

experiences of confirmed/suspected cases of MBDs. In Salvador, more women disclosing having experience of ZIKV infection, whereas in Jundiaí few participants knew someone that had been infected.

> *[P1]: I think [during] the outbreak I [became] more attentive…everyone was contracting Zika…Wow! My father had it too, and he had that anxiety thing – if you saw anything, even if it had water in a little while, you'd turn it [upside down].*
>
> **Salvador-FGD4**

Attitudes and Normative Beliefs

There was consensus across all groups that pregnant women were most susceptible to ZIKV infection, followed by children, the elderly and those with chronic health conditions. Participants described avoiding travel to areas perceived to present an elevated risk of MBDs, and some understood outbreak seasonality. Several described the belief that infection by one MBD increased their susceptibility to others, although there was a lot of uncertainty and misinformation around ZIKV case confirmation. The spread of misinformation was a concern to participants, and several misinterpreted or described as not trusting public information about the origin of the virus.

> *[P2]: In my opinion, I knew that Dengue and Zika is the same thing…I think that's evolution from one disease to another.*
>
> **Salvador-FGD6**

Living in an area of perceived low risk was often described to diminish participants interest in adopting preventive measures ("*It's only worrisome when there's an epidemic*," **Jundiaí-FGD1**). However, there was less consensus between FGDs regarding where population density of *Ae. aegypti* vectors was highest, and several participants described the mosquito as absent from their neighbourhood altogether. Perceived severity of ZIKV infection also varied considerably. Some likened ZIKV symptoms to mild influenza, although women perceived there to be a higher threat from ZIKV than men, with some participants recalling inflammation of the joints and fatigue as symptoms that required extended recovery. Some also described the risk of death as a potential consequence of ZIKV infection.

> *[P1]: It caused a panic, right? Many women gave up being mothers, or they delayed, right? Fear of disease.*
>
> *[P2]: In fact, all the [mosquito-borne] diseases mentioned are worrisome, right? Even the flu is scary.*
>
> **Salvador-FGD8**

Some participants also described differences in the appearance of bites from mosquitoes carrying ZIKV. Several likened the experience to an allergic reaction, which is perhaps a perception of maculopapular rash

For peer review only - http://bmjopen.bmj.com/site/about/guidelines.xhtml

linked to ZIKV infection.[43] Several women demonstrated higher awareness of ZIKV sequelae from work or study in health care, or volunteering with local ZIKV projects. Although collective awareness was described to have peaked and waned, several participants commented on the visibility of families caring for a child with CZS in broadcast media, and they believed a greater disease burden was in more deprived communities.

> *…usually the people most affected [by CZS] are low-level people right…people who have poor conditions, who live in more inappropriate places.*
> **Jundiaí-Male-SSI-8**

Several participants disclosed they would be willing to access abortion services to reduce risk of having a child with CZS or having previously terminated a pregnancy. However, perceptions of rights to terminate a pregnancy were influenced by strong social norms and religious beliefs, and there was often reluctance to disclose or elaborate on personal attitudes due to its criminalisation. Some conceded community attitudes and norms towards abortion were more nuanced given perceptions of quality-of-life and severe disability associated with CZS. However, for one focus group, partial legislation of abortion in the case of microcephaly was criticised as inadequate and perpetuating discrimination.

> *…I think it depends on where she congregates because religion weighs in a lot…She will not do it because of religion, and if she dares [abort], she will not be accepted.*
> **Jundiaí-FGD4**

> *[P1]: Anencephaly in cases of problems was allowed because it makes life unfeasible, but microcephaly does not...So, you're just going to admit normal kids? It'd be a way of sanitizing the population…*
> **Salvador-FGD3**

Women aged 18–30 were more supportive of the right to abort, as were participants that disclosed as working in health or having accessed higher-education. Despite adequate levels of perceived threat from ZIKV and recognition of potential benefits of a behaviour, participants described many barriers to reproductive health decision-making. There was frustration around the burdens of preventing ZIKV and caring for children with CZS falling on women. Discordant attitudes towards abortion between pregnant women and male partners were also discussed. For example, women reported diminished self-efficacy to negotiate condom use with an intimate partner during the epidemic, attributed to stability of the relationship or harmful gender norms.

> *[P2]: We've already talked about machismo, right? I've heard of a husband dropping his wife and saying "No, if you do not [abort], I'll let you go," because she already knew she had a microcephaly [baby].*

For peer review only - http://bmjopen.bmj.com/site/about/guidelines.xhtml

…

[P3]: Yes, but the question of the condom? OK! One part would accept, but this question of non-penetrative sex for six months? No!

Salvador-FGD5

With regards to personal mosquito-bite prevention, for several participants, skin allergies were also a barrier to the use of topical repellents for personal protection. While this motivated some to consider investing in alternative brands or non-chemical bite-reduction strategies, there was broadly low participant awareness of novel repellent tools such as clothing. Whilst participants were relatively unfamiliar with repellent treated clothing, participants recognized the benefit of these for protecting children and pregnant women, although overheating during pregnancy was a concern. However, in both cities, repellents were described as less accessible for individuals of lower-socioeconomic position. Owning a single item was not perceived to provide sufficient protection, yet buying 'a whole wardrobe' a significant investment. Interest was also strongly affected by their appearance in the community, including negative perceptions of the association between MBDs and social deprivation.

[P4]: It's just one more expense, right? [ALL]: Yes!

[P5]: It would probably be very expensive. Because it would be, say, for the elite.

Salvador-FGD5

Maybe he is bothered about having to wear clothes that would be, in this case, also an indicator of poverty, right?

Male-Salvador-SSI-4

Overall, during interviews there was a positive reception to adoption of novel repellent tools. However, similarity was observed between shorter responses and interviewer prompts, and men often expressed disinterest. Scepticism around long-term effectiveness of repellent clothing was also observed, including concern for areas of skin left exposed.

[P4]:…an entire population can't be protected that way. In particularly endemic regions and for high-risk group like babies or pregnant [women] it works, but it's not good for you to dress a whole neighbourhood with the same clothes!

Salvador-FGD4

At the community level, contextual factors were often linked to MBD outbreaks, such inadequate coverage of urban planning, e.g. sanitation services. In Salvador, the former administration was criticised for poor management of the ZIKV epidemic, including the cost of testing, financial support for families with CZS children and an over-reliance on mass-media campaigns. Surveillance teams were often perceived as

For peer review only - http://bmjopen.bmj.com/site/about/guidelines.xhtml

undermotivated, not being trusted to adequately search for cryptic breeding sites and refusing to enter all households, which was sometimes attributed to both concerns around neighbourhood violence and upstream coordination of vector control efforts.

> *[P1]: Where are the community agents themselves? I'm not talking about treatment, I'm talking about preventive measures. Community agents are not effective by municipal power…it's a type of unstable work, you know? There are months without receiving [them].*
> **Salvador-FGD3**

> *There is a lot of suspicion…total distrust in the [Zika] project…The resistance with men is great.*
> **Jundiaí-FGD4**

Behaviour Change

The most frequent vector control strategy described by participants at the household level was preventing water stagnating by recycling, using sand, covering open receptacles and applying detergents or treatments to bodies of water. Bite-reduction strategies included physical barriers: fans, air-conditioning, bed-nets, window screens, long clothing. Several described using plug-in appliances or burning coils to repel mosquitoes with increased frequency during the epidemic. Electric-shock devices to kill adult mosquitoes were also popular. Some participants, particularly pregnant women, avoided travel to places or during times when mosquitoes are most active. Women in every focus-group described knowing someone in their social circle that delayed pregnancy to mitigate the risk of CZS.

> *I have two sisters-in-law who wanted to get pregnant, but because of the epidemic they were afraid and postponed it.*
> **Jundiaí-FGD3**

Community participation comprised reporting mosquito breeding sites to public health authorities, which was frequently discussed in Jundiaí. Several women described generally observing and encouraging behaviour change in others, including the use of repellents and general maintenance of potential *Aedes* breeding sites.

> *[P1]:…it's not just the authorities, everyone has to do their part…to be able to openly reach the neighbour and say, "Oh, look at your bottles [they're] full of water, focus!"*
> **Salvador-FGD8**

Although some participants described skin irritation from topical repellents, only one participant recalled women avoiding chemical repellents during pregnancy due to safety concerns. Methods for mixing plant-based oils or alcohol with chemical formulations and sunscreen were described to soothe and prevent bites

For peer review only - http://bmjopen.bmj.com/site/about/guidelines.xhtml

from becoming infected. Doing so was also used to mask the smell of repellent products and reduce the cost of re-purchase.

> *[In]Bahia, the desperation is greater than here, and pregnant women are afraid to use any product and use homemade products [instead…*
> **Jundiaí-FGD2**

Community Preferences

Subsidy of contraceptives and repellents were suggested for lower-income or high-risk groups during outbreaks, or recommended that they were freely distributed by local health clinics, NGOs or Brazil's national social welfare program, Bolsa Família.

> *[P4]: The government should give repellent to the people since you have this yellow fever outbreak. Make a campaign. The same people who have family-grants should be entitled…*
> **Salvador-FGD6**

When asked what participants thought of treated clothing, repellent school uniforms to reduce children's risk of MBDs and adult sleepwear to mitigate discomfort from bednets or topical repellents were of interest. Microencapsulation of repellents in wearable plastics were also suggested by some, such as bracelets. Generally, participants expressed interest in clothing items if they were affordable, aligned with local preferences in fashion (e.g. fabric quality, design) and the smell of repellent product could not be easily identified. However, the ability to renew the effectiveness of existing items was important.

> *[P1]: ...you would have to change your wardrobe to buy only mosquito repellent clothes. It would be [a] more effective process [if] you make your clothes have this substance.*
> *[P2]: It makes more sense. Like a lotion.*
> *[P1]: A lotion that you put on to do laundry...*
> *[P3]: Yeah, like a fabric softener.*
> **Salvador-FGD4**

For vector control, often improvements in municipal service coordination was expressed as a priority need, citing open drains or infrequent collection of household waste. One focus-group was interested in reintroducing thermal spraying of insecticides. Another explored the idea of financing the coordination of neighbourhood associations to mobilise the community, including financial compensation of volunteers.

> *[P1]: How are we going to complain about our problems? We do not have a person who can get there and settle for us. If we make a petition, everybody in the neighbourhood will sign, but who will take it?…our neighbourhood is abandoned, we have no association…*

For peer review only - http://bmjopen.bmj.com/site/about/guidelines.xhtml

[P2]: I think every neighbourhood should have [an association].

[P1]: [The former volunteer] did everything for us there. My street was clean, everything was clean. There should be someone to count, take care, understand?

[P3]: If she's doing it, she has to get something too...

[P4]: But the staff thinks the person [must] work for free.

Salvador-FGD6

There was disagreement regarding the saturation of ZIKV messaging during public health campaigns. The majority of women expressed feeling under-equipped with the practical knowledge for prevention, whereas others asserted some messages were overly technical and did not provide sufficient support to implement vector control strategies at the household level. Preference was placed on sustained delivery of messages between outbreaks, via social media or print materials in public spaces. A media campaign that targeted men was suggested as one approach to escalate perceptions of the health risks and consequences for intimate partners due to sexual transmission of ZIKV. A sexual and reproductive health-focused curriculum for schools was described as another point of delivery to improve community engagement with messaging. Health promotion materials to facilitate community events were also suggested to amplify the effect of annual awareness campaigns like 'World Dengue Day'.

[P4]: If it's not in the extreme, [messaging] will not work. It's like cigarette campaigns.

Salvador-FGD5

No, it's not a lack of information, it's education…it has to start very early with sex education. Because human beings only change their habits when something very serious happens. I think information alone does not [do it].

Jundiaí-FGD3

DISCUSSION

In the outbreak beginning 2015, Brazil experienced more cases of ZIKV than any other country. Its MoH responded with a policy strategy focused on vector control, provision of health care access, and technology and research development.[44] However, it has been argued that these policies failed to reach those most vulnerable to the virus.[20,45] The northeast of Brazil was particularly hard-hit, as a region with some of the lowest state Human Development Indices (HDI) in the country.[38,46] In comparison, in 2017 Jundiaí was ranked as having the 11th highest HDI of 5,564 municipalities in Brazil.[37] Individuals from communities in Salvador and Jundiaí were invited to provide their knowledge and perceptions of ZIKV and MBD control for this investigation.

For peer review only - http://bmjopen.bmj.com/site/about/guidelines.xhtml

Community Awareness of MBDs

The sessions revealed that participant understanding of their susceptibility to infection was a key influence on their decision-making to engage in health protection measures. Direct or indirect experience of ZIKV and dengue was a common internal cue to action in Salvador, a city with a long history of MBD outbreaks,[47] which is consistent with previous findings.[27,38] However, participants frequently believed that ZIKV-carrying *Aedes* mosquitos were absent in their local area, and perceptions varied as to where in Brazil the prevalence of MBDs was greatest. At the time of the study, a national yellow fever vaccination campaign was communicating outbreaks in non-human primates, and some participants discussed fearing reports of its urbanization.[23,48] Participants describing a potential relationship between ZIKV and other MBDs was not unwarranted, as arboviruses transmitted by *Aedes* tend to cluster.[13] Sequential arboviral infection is also still poorly understood,[46] with some studies suggesting limited cross-immunity following dengue virus infection.[49–51]

The majority of women interviewed were unaware of the risk of ZIKV transmission from unprotected sex. This is consistent with findings from other studies on ZIKV risk communication,[34] including in Colombia.[52] Since interviews were conducted towards the end of the outbreak, this suggests there was a missed opportunity to prevent at least some of the spread of ZIKV. Although the ultimate importance of sexual transmission may be small compared to that of mosquito-borne transmission,[53] the public should receive clear messaging around the relative contributions of mosquito-borne, vertical, sexual, and bloodborne transmission, to enable individuals to make informed choices about adopting preventive measures.

Social Determinants of ZIKV and CZS

There was also strong disagreement around the criminalisation of abortion, which has been dismissed as a paternalistic policy that is inconsistent with MoH advice to avoid or delay pregnancy in ZIKV endemic areas.[20,21,54] The sense that ZIKV has been emasculated, where the responsibility to prevent sexual transmission has fallen to women, has also been described in other studies.[54–58] Despite being strongly advocated by international multi-lateral agencies and Brazilian legislators,[20,21] important questions remain outstanding on reproductive health rights for ZIKV seropositive individuals.[59,60]

MBDs, including ZIKV, predominantly affect individuals in economically deprived areas.[30,31] Inadequate access to clean water, sanitation and other infrastructural deficits allow mosquito populations to thrive.[26] In addition, individuals in these communities may also be less able to afford tools for personal protection and have poorer access to good quality health care.[46,61,62] In our focus groups, the perceived severity of ZIKV was most often framed through the lens of disadvantage: the availability and affordability of amniocentesis or ZIKV testing; female agency to negotiate abstinence or long-term condom use with their male partners; access and acceptance of contraceptives to delay pregnancy or abortion; and uncertainty around a financial and social support network to care for children with CZS. These themes were consistent with other study findings.[58,61–63]

For peer review only - http://bmjopen.bmj.com/site/about/guidelines.xhtml

Personal Protection Strategies

Topical repellents are uncomfortable for some users, and may not be seen as long-term solutions for preventing mosquito bites.[15,64] The pay-off for repeat application of repellents may also be less certain for ZIKV than other MBDs, where the onset of symptoms and potential consequences of infection is comparatively short.[65] Novel, non-topical repellent technologies are not yet widely known or understood, and perceived safety of synthetic repellents was anticipated to be a key barrier to their adoption, as seen in other qualitative studies.[35,52] Instead, the key barriers discussed were the effectiveness and accessibility of novel repellent tools such as clothing.

In Salvador, it was also important that repellent clothing was not perceived to be a 'uniform' associated with low-socioeconomic position, whilst in Jundiaí, participants discussed the need for clothing designs to reflect local preferences in fashion. The concept of repellent school uniforms to protect school-going children from MBDs was well received and has demonstrated strong potential in a cluster randomised-controlled trial in Thailand.[17] Participants also expressed an interest in being able to renew the repellent effect of clothing to overcome barriers like affordability and durability, negating the need for replacements. For example, using sprays to reapply repellents to clothing was perceived as more feasible option to clothes treated prior to purchase. Some also acknowledged the attractiveness of formulated washes for ease of application, and incorporation of perfumes to mask repellent smell.

Vector Control Strategies

Mosquito prevention at the household level was often perceived to be a burden. However, many participants described removal or treatment of potential mosquito-breeding sites as being incorporated into daily routines. Despite this, several individuals expressed their personal control beliefs for vector control were fatigued when neighbours did not also do their part. Abandoned buildings or communal space 'contaminating' maintained areas contributed to some participants' sense of futility; even if they were well-informed, a public health challenge as prevalent as *Aedes* was not something the community could 'combat' alone.

Minor themes of blame, mistrust and responsibility were also frequently allocated upstream, especially in Salvador. Reporting mosquito-breeding sites in communal areas in more deprived neighbourhoods to the City Hall was deemed unlikely to result in change due to broader inadequacies in local urban planning. Some participants also expressed frustration due to a lack of consistent or thorough household inspections by surveillance teams, confusion around the different stakeholders involved and follow-up visits, or a need for clarification of ZIKV key messages. Often this was attributed to chronic under-investment in vector control, a common theme in other studies in South America, where both men and women have expressed a need for intensification of government support.[52,55,57]

For peer review only - http://bmjopen.bmj.com/site/about/guidelines.xhtml

Community Engagement Related to ZIKV Prevention

Freire posits that structural inequalities in Brazil creates a loss of agency,[66] which in the context of the ZIKV epidemic, likely constrained self-efficacy for behaviour change.[67] A systems model for *Aedes* vector control also argues that the pathway between collective awareness, collective action, community attitudes and normative beliefs is simply too long for effective control of MBD outbreaks.[28] The opportunity to communicate barriers in a more timely manner would improve collective awareness, as well as political will for local authorities to act.[13] Carvalho *et al.* proposed one solution could be investing in improved frequency of household visits from community health workers (CHWs) under the Family Health Strategy,[28] which covers 66.5% of Brazil's population.[68] Although task-shifting of CHW responsibilities to include ZIKV case reporting was possible during the epidemic, their catchment area excluded middle- or high-income neighbourhoods,[69] like Jundiaí.

Instead, a community-participation model is proposed as an alternative. Grassroots approaches, such as neighbourhood associations, may serve as a more trusted setting for community engagement during infectious disease outbreaks.[13,70] For example, in a meta-analysis on uptake of novel repellent technologies, participatory models were found most effective at improving self-efficacy,[71] as well as promoting a sense of community responsibility.[72] Financing mechanisms to decentralise and triage risk communication and vector control at the community level may also mitigate the marginalisation of individuals in more deprived settings, largely caused by top-down approaches in health promotion.[66]

Limitations

Some participants were not familiar with questions raised on novel repellents in the topic guide. Additionally, the differentiation between different prevention measures for ZIKV may not have always been clearly understood. Interview prompts, such as preferences for novel repellents, may have therefore enabled acquiescence response bias.[41] When focus groups discussed more contentious topics, such as abortion, personal attitudes may have also been conflated with social norms, which may have led to some women feeling unable to disclose disagreement with the majority.[73] Although facilitators were able to detect non-verbal cues for each, subtext may have been lost during analysis. To mitigate this, an independent translation service was used to verify the credibility of transcript excerpts, and preliminary findings were discussed with principal investigators for triangulation. Additional data were not collected on participants, such as data on socioeconomic position, which along with missing data on age for some Jundiaí focus groups could have provided an interesting overview of the participants in this study. The selection of the HBM as a conceptual framework is also necessarily limited,[74] particularly given the scope of themes raised in the topic guide and context-specific challenges reported by other researchers during the 2015–16 ZIKV epidemic.[29] Nonetheless, the HBM still permitted a relatively deep analysis of individual-level factors, despite disagreement in the literature over which order the components may lead to behaviour change.[74] The literature was thus consulted post-analysis for transferability of findings.

For peer review only - http://bmjopen.bmj.com/site/about/guidelines.xhtml

Recommendations

This investigation recommends that national authorities provide effective repellent tools to families entitled to social-welfare in settings where MBD outbreaks are regular occurrences, and during outbreaks extend this provision to include high risk groups. Capacity-building of MBD surveillance teams is also recommended to strengthen multi-level governance and reduce gaps in the frequency of interventions designed to prevent infectious disease transmission, such as household inspections. A degree of data saturation for preferred criteria of novel repellents in this study lends weight to the finding there was an unmet need for alternative personal protective tools to topical repellents.

The WHO Global Vector Control Response advises cross-disciplinary community engagement to improve context-sensitive messaging and reduce barriers to uptake of MBD preventive strategies.[26] Designing a mass-media campaign that targets men could improve awareness of ZIKV sexual transmission and emphasise the importance of protecting the health of their female intimate partners. Financing participatory models for community engagement would also demonstrate a firm commitment to translating politicised slogans into an effective, bottom-up control strategy for *Aedes*-related MBDs.

It is worth noting our recommendations are also pertinent to the response to the SARS-CoV-2 pandemic. At the time of writing, Brazil also had amongst the highest numbers of confirmed COVID-19 cases in the world, particularly in the North, and its MoH was criticised for not developing a national plan to combat the disease.[75] In light of this, further focus group studies, or design of a Likert scale-based survey that operationalises the HBM during data collection,[32] may also prove fruitful for understanding how perceived severity and susceptibility to MBDs has changed in Salvador and Jundiaí, particularly following outbreaks of chikungunya and yellow fever.[23,76]

Conclusion

This study makes a strong case for the value of qualitative investigations and transferability of the HBM to inform bottom-up approaches in health protection. Since the initial outbreak in Brazil in 2015, the fall of the perceived threat from ZIKV, normalization of CZS symptoms in affected children, and the poorly understood relationship to other arboviruses transmitted by *Aedes* has weakened community self-efficacy and perceptions of the government response. Participant awareness of sexual transmission of ZIKV was low and several focus groups discussed an unmet need for a health campaign that targeted men. Significant barriers were also discussed around the affordability of mosquito-bite prevention strategies, such as topical repellents and novel tools for personal protection, including their perception as a potential marker of socioeconomic position. Household behaviours to control the *Aedes* vector were also often fatigued by a lack of cooperation and coordination at the community and municipal level. It is therefore argued that the historical failure to control *Aedes* outbreaks in Brazil lies in placing too much responsibility on the individual, particularly women. By investing in evidence-based epidemic preparedness, and by stimulating a sense of community agency to tackle vector breeding sites, Brazil may indeed be better placed to 'beat' the *Aedes* mosquito.

For peer review only - http://bmjopen.bmj.com/site/about/guidelines.xhtml

Acknowledgements

We would like to thank all study participants, and the ZikaPLAN research teams in Salvador and Jundiaí: Ana Maria Rico, Greice Bezerra Viana, Fernanda Macedo da Silva Lima, Mônica Manir, Tania Boccia and Vera Lucia Zaher-Rutherford. We would also like to thank Alexandra Levitas for her support during analysis.

Contributors

JGL conceived the study. JBI and EM led data collection in Salvador and Jundiaí, coordinated by JK. DB led the analysis and the University College London Digital Media service was used to translate select excerpts of Brazilian transcripts for verification against the translations made by EM. GMP and RTJ performed triangulation of coding. DB, GMP, RP and RTJ authored the manuscript for publication. All authors read and approved the final manuscript.

Funding

This study was financed by the European Union's Horizon 2020 research and innovation programme awarded to Zika Preparedness Latin American Network, (ZikaPLAN) under Grant Agreement No. 734584

Competing interests

All authors declare no competing interests.

Patient consent for publication

Informed written consent was obtained before data collection. Additional consent for publication is not required.

Ethics approval

Approval for the study in both Jundiaí and Salvador was granted by the Jundiaí School of Medicine Ethical Review Board in January 2017 (REF: 1.875.618). For analysis, approval was granted by the MSc Research Ethics Committee at LSHTM in July 2020 (REF: 21978).

Data availability statement

The topic guide, figures and COREQ checklist supporting the conclusions of this article are included within the article and its supplementary files. The consent form and topic guide are available at the London School of Hygiene & Tropical Medicine (LSHTM) Data Compass repository [https://doi.org/10.17037/DATA.00002097]. The transcripts of focus-groups and semi-structured interviews supporting the conclusions of this article cannot be made available for confidentiality reasons.

For peer review only

For peer review only - http://bmjopen.bmj.com/site/about/guidelines.xhtml

REFERENCES

1 Kauffman EB, Kramer LD. Zika virus mosquito vectors: Competence, biology, and vector control. *J Infect Dis* 2017;216:976–90. doi:10.1093/infdis/jix405

2 Turmel JM, Abgueguen P, Hubert B, *et al.* Late sexual transmission of Zika virus related to persistence in the semen. *Lancet* 2016;387:2501. doi:10.1016/S0140-6736(16)30775-9

3 Musso D, Stramer SL, Busch MP. Zika virus: A new challenge for blood transfusion. *Lancet* 2016;387:1993–4. doi:10.1016/S0140-6736(16)30428-7

4 Lopes Moreira ME, Nielsen-Saines K, Brasil P, *et al.* Neurodevelopment in infants exposed to Zika virus in utero. *N Engl J Med* 2018;379(24):2377–9. doi:10.1056/NEJMc1800098

5 Brasil P, Pereira JP, Moreira ME, *et al.* Zika virus infection in pregnant women in Rio de Janeiro. *N Engl J Med* 2016;375:2321–34. doi:10.1056/NEJMoa1602412

6 de Araújo TVB, Ximenes RAA, Miranda-Filho DB, *et al.* Association between microcephaly, Zika virus infection, and other risk factors in Brazil: final report of a case-control study. *Lancet Infect Dis* 2018;18(3):328–336. doi:10.1016/S1473-3099(17)30727-2

7 Ministry of Health (BR). Portaria no 1.813, de 11 de Novembro de 2015. Declara Emergência em Saúde Pública de Importância Nacional (ESPIN) por alteração do padrão de ocorrência de microcefalias no Brasil. Brazil: Ministério da Saúde 2016. http://bvsms.saude.gov.br/bvs/saudelegis/gm/2015/prt1813_11_11_2015.html [Accessed 02 September 2021]

8 World Health Organization. WHO statement on the first meeting of the International Health Regulations (2005) (IHR 2005) Emergency Committee on Zika virus and observed increase in neurological disorders and neonatal malformations. Geneva: World Health Organization 2016. https://www.who.int/news/item/01-02-2016-who-statement-on-the-first-meeting-of-the-international-health-regulations-(2005)-(ihr-2005)-emergency-committee-on-zika-virus-and-observed-increase-in-neurological-disorders-and-neonatal-malformations [Accessed 08 September 2021]

9 Pan American Health Organization. Zika suspected and confirmed cases reported by countries and territories in the Americas cumulative cases, 2015-2017. [Update] 4 January, 2018. Washington, D.C: Regional Office for the Americas of the World Health Organization 2017. www.paho.org/hq/index.php?option=com_content&view=article&id=12390:zika-cumulative-cases [Accessed 6 Dec 2020]

10 Singh RK, Dhama K, Khandia R, *et al.* Prevention and control strategies to counter Zika virus, a special focus on intervention approaches against vector mosquitoes—Current Updates. *Front Microbiol* 2018;9(87) doi: 10.3389/fmicb.2018.00087

11 Cavalcanti LPG, Oliveira RMAB, Alencar CA. Changes in infestation sites of female *Aedes aegypti* in Northeast Brazil. *Rev Soc Bras Med Trop* 2016;49(04). doi:10.1590/0037-8682-0044-2016

12 Achee NL, Grieco JP, Vatandoost H, *et al.* Alternative strategies for mosquito-borne arbovirus control. *PLoS Negl Trop Dis* 2019;13:1–22. doi:10.1371/journal.pntd.0006822

For peer review only - http://bmjopen.bmj.com/site/about/guidelines.xhtml

13 Bardosh KL, Ryan S, Ebi K, *et al*. Addressing vulnerability, building resilience: Community-based adaptation to vector-borne diseases in the context of global change. *Infect Dis Poverty* 2017;6:166. doi:10.1186/s40249-017-0375-2

14 Orsborne J, Banks SDR, Hendy A, *et al.* Personal protection of permethrin-treated clothing against *Aedes aegypti*, the vector of dengue and Zika virus, in the laboratory. *PLoS One* 2016;11:1–18. doi:10.1371/journal.pone.0152805

15 Banks SDR, Murray N, Wilder-Smith A, *et al.* Insecticide-treated clothes for the control of vector-borne diseases: A review on effectiveness and safety. *Med Vet Entomol* 2014;28:14–25. doi:10.1111/mve.12068

16 Banks SDR, Orsborne J, Gezan SA, *et al.* Permethrin-treated clothing as protection against the Dengue vector, *Aedes aegypti*: Extent and duration of protection. *PLoS Negl Trop Dis* 2015;9:1–16. doi:10.1371/journal.pntd.0004109

17 Kittayapong P, Olanratmanee P, Maskhao P, *et al.* Mitigating diseases transmitted by *Aedes* mosquitoes: A cluster-randomised trial of permethrin-impregnated school uniforms. *PLoS Negl Trop Dis* 2017;11:1–12. doi:10.1371/journal.pntd.0005197

18 Wilder-Smith A, Preet R, Brickley EB, *et al.* ZikaPLAN: Addressing the knowledge gaps and working towards a research preparedness network in the Americas. *Glob Health Action* 2019;12:1666566. doi:10.1080/16549716.2019.1666566

19 Ministry of Health (BR). Plano nacional de enfrentamento à microcefelia: Protocolo de atenção à saúde e resposta à ocorrência de microcefalia (v3). Brazil: Ministério da Saúde 2016. https://bvsms.saude.gov.br/bvs/publicacoes/protocolo_atencao_saude_resposta_ocorrencia_microcefalia.pdf [Accessed 08 February 2021]

20 Collucci C. Brazilian attorneys demand abortion rights for women infected with Zika. *BMJ* 2016;354:i4657. doi:10.1136/bmj.i4657

21 Carabali M, Austin N, King NB, *et al*. The Zika epidemic and abortion in Latin America: a scoping review. *Glob Health Res Policy* 2018;3:15. doi: 10.1186/s41256-018-0069-8.

22 Gómez EJ, Perez FA, Ventura D. What explains the lacklustre response to Zika in Brazil? Exploring institutional, economic and health system context. *BMJ Glob Heal* 2018;3:e000862. doi:10.1136/bmjgh-2018-000862

23 Pan American Health Organization. Epidemiological alerts and updates: Annual report 2017. Washington, D.C: Regional Office for the Americas of the World Health Organization 2018. www.paho.org/en/documents/epidemiological-alerts-and-updates-annual-report-2017 [Accessed 14 Jan 2020]

24 Pattnaik A, Sahoo BR, Pattnaik AK. Current status of Zika virus vaccines: successes and challenges. *Vaccines* 2020;8(2):266. doi: 10.3390/vaccines8020266

25 Wilson AL, Courtenay O, Kelly-Hope LA, *et al.* The importance of vector control for the control and elimination of vector-borne diseases. *PLoS Negl Trop Dis* 2020;14:e0007831. doi:10.1371/journal.pntd.0007831

For peer review only - http://bmjopen.bmj.com/site/about/guidelines.xhtml

26 The Global vector control response 2017–2030 (GVCR). Geneva: World Health Organization 2017. www.who.int/vector-control/publications/global-control-response/en/ [Accessed 1 January 2021]

27 Paixão ES, Teixeira MG, Rodrigues LC. Zika, chikungunya and dengue: The causes and threats of new and reemerging arboviral diseases. *BMJ Glob Heal* 2018;3:e000530. doi:10.1136/bmjgh-2017-000530

28 Carvalho MS, Honorio NA, Garcia LMT, *et al. Aedes aegypti* control in urban areas: A systemic approach to a complex dynamic. *PLoS Negl Trop Dis* 2017;11:1–15. doi:10.1371/journal.pntd.0005632

29 Passos MJ, Matta G, Lyra TM, et al. The promise and pitfalls of social science research in an emergency: lessons from studying the Zika epidemic in Brazil, 2015–2016. BMJ Global Health 2020;5(4):e002307. doi:10.1136/bmjgh-2020-002307

30 Power GM, Francis SC, Sanchez Clemente N, *et al.* Examining the association of socioeconomic position with microcephaly and delayed childhood neurodevelopment among children with prenatal Zika virus exposure. *Viruses* 2020;12. doi:10.3390/v12111342

31 Souza WV De, Albuquerque MDFPM, Vazquez E, *et al.* Microcephaly epidemic related to the Zika virus and living conditions in Recife, Northeast Brazil. *BMC Public Health* 2018;18. doi:10.1186/s12889-018-5039-z

32 Champion VL, Skinner CS. The Health Belief Model. In: Glanz, K., Rimer, B.K. & Viswanath, K., eds. Health Behavior and Health Education: Theory, Research, and Practice. San Francisco: Jossey-Bass 2008:45–66

33 Rosenstock IM, Strecher VJ, Becker MH. Social Learning Theory and the Health Belief Model. *Heal Educ Behav* 1988;15:175–83. doi:10.1177/109019818801500203

34 Clancy IL, Jones RT, Power GM, *et al.* Public health messages on arboviruses transmitted by *Aedes aegypti* in Brazil. *BMC Public Health* 2021;21(1):1362. doi: 10.1186/s12889-021-11339-x

35 Wong LP, AbuBakar S. Health beliefs and practices related to Dengue fever: A focus group study. *PLoS Negl Trop Dis* 2013;7:2310. doi:10.1371/journal.pntd.0002310

36 Brazilian Institue of Geography and Statistics. Estimates of resident population in Brazilian municipalities on July 1, 2020. Rio de Janeiro: Instituto Brasileiro de Geografia e Estatística (IBGE) 2020. https://ftp.ibge.gov.br/Estimativas_de_Populacao/Estimativas_2020/estimativa_dou_2020.pdf [Accessed 2 Dec 2020].

37 Sanchez Clemente N, Rodrigues M, Pascalicchio AP, *et al.* Cohort profile: The Jundiaí Zika cohort (JZC), a pregnancy and birth cohort in São Paulo state, Brazil. *BMJ Open* 2019;9:e027947. doi:10.1136/bmjopen-2018-027947

38 Netto EM, Moreira-soto A, Pedroso C, *et al.* High Zika virus seroprevalence in Salvador, northeastern Brazil limits the potential for further outbreaks. *Am Soc Microbiol* 2017;8:1–14. doi:10.1128/mBio.01390-17

[dataset] [39] Bancroft D, Power G, Jones R, *et al.* ZikaPLAN – Research tools used for focus group discussion and interviews. [Data Collection]. London: LSHTM DataCompass, London School of

For peer review only - http://bmjopen.bmj.com/site/about/guidelines.xhtml

Hygiene and Tropical Medicine 2021. https://doi.org/10.17037/DATA.00002097

40 Braun, V, Clarke V. Using thematic analysis in psychology. *Qual Res Psychol* 2006;3:77–101. doi:10.1191/1478088706qp063oa

41 Krueger RA, Casey MA. Focus groups: A practical guide for applied research (5th Ed). California: SAGE Publications 2015.

42 Tong A, Sainsbury P, Craig J. Consolidated criteria for reporting qualitative research (COREQ): A 32-item checklist for interviews and focus groups. *Int J Qual Heal Care* 2007;19:349–57. doi:10.1093/intqhc/mzm042

43 Paniz-Mondolfi AE, Blohm GM, Hernandez-Perez M, *et al*. Cutaneous features of Zika virus infection: a clinicopathological overview. *Clinical and Experimental Dermatology* 2019;44(1):13-19. doi:10.1111/ced.13793

44 Henriques CMP, Duarte E, Garcia LP. Desafios para o enfrentamento da epidemiologia de microcefalia. *Epidemiol Serv Saúde* 2016;25:7–10. doi:10.5123/S1679-49742016000100001

45 Nading A, Lowe L. Social justice as epidemic control: Two Latin American case studies. M*ed Anthropol Cross Cult Stud Heal Illn* 2018;37:458–71. doi:10.1080/01459740.2018.1485021

46 Peiter PC, Pereira R dos S, Moreira MCN, *et al.* Zika epidemic and microcephaly in Brazil: Challenges for access to health care and promotion in three epidemic areas. *PLoS One* 2020;15:e0235010. doi:10.1371/journal.pone.0235010

47 Barreto FR, Teixeira MG, Costa MDCN, *et al.* Spread pattern of the first dengue epidemic in the city of Salvador, Brazil. *BMC Public Health* 2008;8:51. doi:10.1186/1471-2458-8-51

48 Callender DM. Factors contributing to and strategies to combat emerging arboviruses. *Glob Public Health* 2018;13:1846–52. doi:10.1080/17441692.2018.1464588

49 Khandia R, Munjal A, Dhama K, *et al.* Modulation of Dengue/Zika virus pathogenicity by antibody-dependent enhancement and strategies to protect against enhancement in Zika virus infection. *Front Immunol* 2018;9:597. doi:10.3389/fimmu.2018.00597

50 Gordon A, Gresh L, Ojeda S, *et al.* Prior dengue virus infection and risk of Zika: A pediatric cohort in Nicaragua. *PLoS Med* 2019;16:e1002726. doi:10.1371/journal.pmed.1002726

51 Serrano-Collazo C, Pérez-Guzmán EX, Pantoja P, *et al.* Effective control of early Zika virus replication by dengue immunity is associated to the length of time between the 2 infections but not mediated by antibodies. *PLoS Negl Trop Dis* 2020;14:1–28. doi:10.1371/journal.pntd.0008285

52 Mendoza C, Jaramillo G-I, Ant TH, *et al.* An investigation into the knowledge, perceptions and role of personal protective technologies in Zika prevention in Colombia. *PLoS Negl Trop Dis* 2020;14:e0007970. doi:10.1371/journal.pntd.0007970

53 Althaus CL, Low N. How relevant is sexual transmission of Zika virus? *PLoS Med* 2016;13:e1002157. doi:10.1371/journal.pmed.1002157

54 Campos TC de. Zika, public health, and the distraction of abortion. *Med Heal Care Philos* 2017;20:443–6. doi:10.1007/s11019-016-9739-9

55 Weldon CT, Riley-Powell AR, Aguerre IM, *et al.* 'Zika is everywhere': A qualitative exploration of

For peer review only

For peer review only - http://bmjopen.bmj.com/site/about/guidelines.xhtml

knowledge, attitudes and practices towards Zika virus among women of reproductive age in Iquitos, Peru. *PLoS Negl Trop Dis* 2018;12:e0006708. doi:10.1371/journal.pntd.0006708

56 Borges ALV, Moreau C, Burke A, *et al.* Women's reproductive health knowledge, attitudes and practices in relation to the Zika virus outbreak in northeast Brazil. *PLoS One* 2018;13:e0190024. doi:10.1371/journal.pone.0190024

57 Linde-Arias AR, Roura M, Siqueira E. Solidarity, vulnerability and mistrust: How context, information and government affect the lives of women in times of Zika. *BMC Infect Dis* 2020;20:263. doi:10.1186/s12879-020-04987-8

58 Diniz D, Ali M, Ambrogi I, *et al.* Understanding sexual and reproductive health needs of young women living in Zika affected regions: A qualitative study in northeastern Brazil. *Reprod Health* 2020;17. doi:10.1186/s12978-020-0869-4

59 Wenham C, Arevalo A, Coast E, *et al.* Zika, abortion and health emergencies: a review of contemporary debates. *Global Health* 2019;15:49. doi:10.1186/s12992-019-0489-3

60 Prata ARS, Pedroso D, Menezes G, *et al.* Juridical perspectives of interruption of pregnancy with zika virus infection regarding medical emotional and social consequences. *J Hum Growth Dev* 2018;28:77–81. doi:10.7322/jhgd.143875

61 Albuquerque MSV, Lyra TM, Melo APL, *et al.* Access to healthcare for children with Congenital Zika Syndrome in Brazil: Perspectives of mothers and health professionals. *Health Policy Plan* 2019;34:499–507. doi:10.1093/heapol/czz059

62 Duttine A, Smythe T, de Sá MRC, *et al.* Congenital Zika Syndrome - Assessing the need for a family support programme in Brazil. *Int J Environ Res Public Health* 2020;17(10) doi:10.3390/ijerph17103559 [published Online First: 2020/05/23]

63 Kuper H, Lopes Moreira ME, de Araújo TVB, *et al.* The association of depression, anxiety, and stress with caring for a child with Congenital Zika Syndrome in Brazil; Results of a cross-sectional study. *PLoS Negl Trop Dis* 2019;13(9):e0007768. doi:10.1371/journal.pntd.0007768

64 Wylie BJ, Hauptman M, Woolf AD, *et al.* Insect repellants during pregnancy in the era of the Zika virus. *Obstet Gynecol* 2016;128:1111–5. doi:10.1097/AOG.0000000000001685

65 Funk S, Bansal S, Bauch C, *et al.* Nine challenges in incorporating the dynamics of behaviour in infectious diseases models. *Epidemics* 2015 Mar;10:21-5. doi:10.1016/j.epidem.2014.09.005

66 Freire P, Bergman Ramos M, Macedo D. Pedagogy of the oppressed. New York: Continuum International 2005:29–33

67 Nading A, Lowe L. Social justice as epidemic control: Two Latin American case studies. *Med Anthropol Cross Cult Stud Heal Illn* 2018;37:458–71. doi:10.1080/01459740.2018.1485021

68 Hone T, Rasella D, Barreto ML, *et al.* Association between expansion of primary healthcare and racial inequalities in mortality amenable to primary care in Brazil: A national longitudinal analysis. PLoS Med 2017;14:1–19. doi:10.1371/journal.pmed.1002306

69 Wadge H, Bhatti Y, Carter A, *et al.* Brazil's Family Health Strategy: Using community health workers to provide primary care. *Commonw Fund* 2016;40.

For peer review only

https://www.commonwealthfund.org/publications/case-study/2016/dec/brazils-family-health-strategy-using-community-health-care-workers [Accessed 5 Dec 2020].

70 McNaughton D. The importance of long-term social research in enabling participation and developing engagement strategies for new Dengue control technologies. *PLoS Negl Trop Dis* 2012;6:1785. doi:10.1371/journal.pntd.0001785

71 Lima EP, Goulart MOF, Rolim Neto ML. Meta-analysis of studies on chemical, physical and biological agents in the control of Aedes aegypti infectious disease epidemiology. *BMC Public Health* 2015;15:858. doi:10.1186/s12889-015-2199-y

72 Nowell B, Boyd NM. Sense of community responsibility in community collaboratives: Advancing a theory of community as resource and responsibility. *Am J Community Psychol* 2014;54:229–42. doi:10.1007/s10464-014-9667-x

73 Cislaghi B, Heise L. Theory and practice of social norms interventions: Eight common pitfalls. *Global Health* 2018;14:1–10. doi:10.1186/s12992-018-0398-x

74 Jones CL, Jensen JD, Scherr CL, *et al.* The Health Belief Model as an explanatory framework in communication research: exploring parallel, serial, and moderated mediation. *Health Commun* 2015;30(6):566-76. doi:10.1080/10410236.2013.873363 [published Online First: 2014/07/11]

75 Rocha R, Atun R, Massuda A, *et al.* Effect of socioeconomic inequalities and vulnerabilities on health-system preparedness and response to COVID-19 in Brazil: a comprehensive analysis. *The Lancet Global Health* 2021;9(6):e782-e92. doi:10.1016/S2214-109X(21)00081-4

76 Atif M, Azeem M, Sarwar MR, *et al.* Congenital Chikungunya virus infection after an outbreak in Salvador, Bahia, Brazil. *PLoS One* 2018;14:325–30. doi:10.1055/s-0036-1587323

Figure 1. The Health Belief model

Adapted from Rosenstock et al. (1988),[33] the HBM predicts the decision making process to engage in a new health seeking behaviour. The individual assesses a perceived threat, potential barriers, benefits and their ability to undergo a behaviour change in response to knowledge, internal or external cues to action.[32]

Figure 2. Concept map of key, major and minor themes for community knowledge, attitudes and perceptions of Zika virus and vector control strategies in Salvador and Jundiaí, Brazil. Four key and 12 major themes were mapped to determine whether they credibly fit constructs for behaviour change outlined in the Health Belief Model.[32,33] The key and major themes are further defined in Table 2.

For peer review only

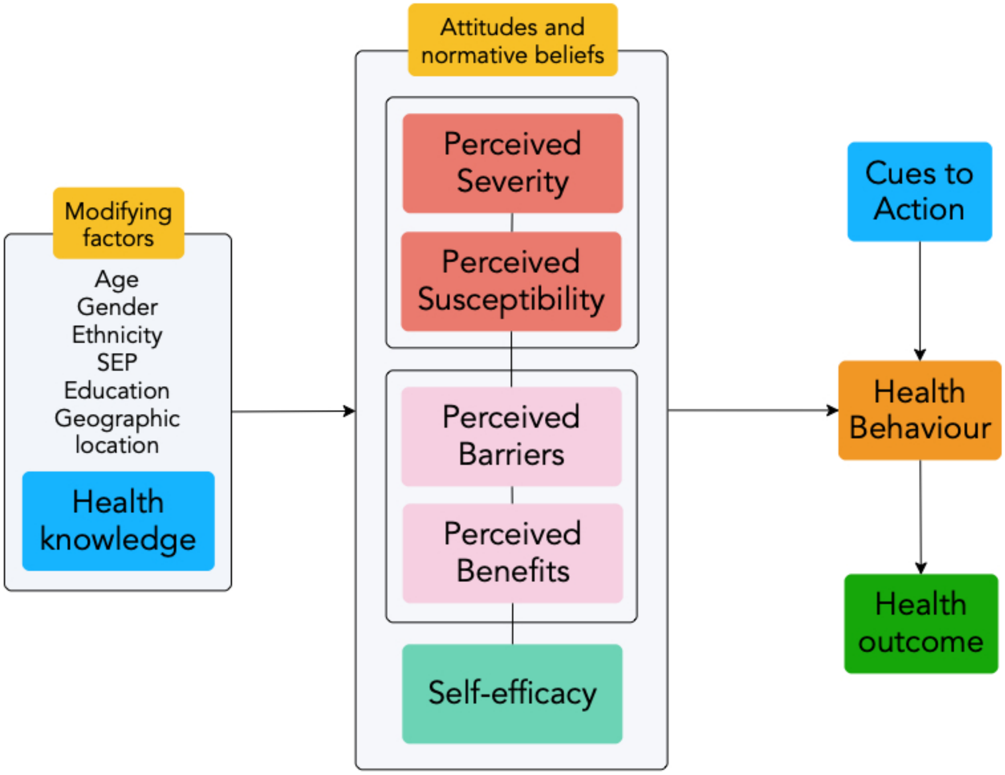

Figure 1. The Health Belief Model Adapted from Rosenstock *et al.* (1988),[33] the HBM predicts the decision making process to engage in a new health seeking behaviour. The individual assesses a perceived threat, potential barriers, benefits and their ability to undergo a behaviour change in response to knowledge and internal or external cues to action.[32]

63x49mm (300 x 300 DPI)

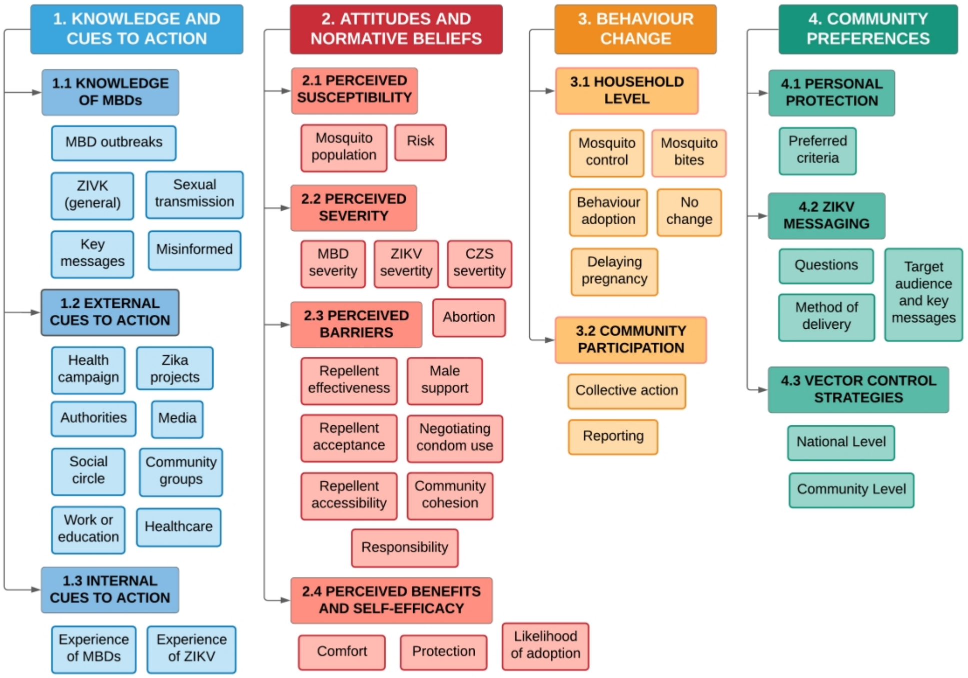

Figure 2. Concept map of key, major and minor themes for community knowledge, attitudes and perceptions of Zika virus and vector control strategies in Salvador and Jundiaí, Brazil. Four key and 12 major themes were mapped to determine whether they credibly fit constructs for behaviour change outlined in the Health Belief Model.[32,33] The key and major themes are further defined in Table 2.

299x212mm (300 x 300 DPI)

Supplementary File 1: Topic Guide

Bancroft *et al.* Vector control strategies in Brazil: A qualitative investigation into community knowledge, attitudes and perceptions following the 2015–16 Zika virus epidemic. *BMJ Open* 2021 [Manuscript ID: bmjopen-2021-050991]

SOCIODEMOGRAPHIC SURVEY:

Socio-demographic data collected during recruitment.
Age: 18–30 [ ] 31–49 [ ] **Socioeconomic status:** High [ ] Middle [ ] Low [ ]

Date of interview	
Location of interview	
Name of interviewer	
Name of observer/note taker	
Time interview started	
Time interview ended	

INTERVIEW OPEN

- **Introduction by interviewer to the study**
- **Review and signing of informed consent form**
- **Start recording**

[12 questions]

1. **Mosquito control by families and the community.**

   a. What do you do in your home to reduce the number of mosquitoes that exist in your region and the number of bites that you and your family receive?
   Prompts: environmental cleaning, repellents, long sleeves, screens, bed nets etc.
   b. Is there any kind of community effort to reduce mosquito outbreaks?
   Detail (investigate this aspect well).

2. **Mosquito control by local authorities**
   a. What mosquito control activities, if any, are undertaken by the local authorities in your community?

3. **Changes in mosquito control practices**
   Has there been a change in mosquito control practices in your community, and in your own personal protection, since the emergence of Zika? If so, please provide details.

4. **Preferred mosquito control activities**
   Zika mosquitoes bite during the day. Given that, what kind of mosquito control would you <u>like</u> to see? *Better detail on personal protection including clothing.*

5. **Personal protection interventions**
   a. What do you think of personal protection interventions / alternatives / practices such as mosquito repellent clothing?
   b. How likely are you to use these alternatives and what would be your considerations?
   Prompt for cost, safety, comfort, fashion, duration of effectiveness.

For peer review only

6. Concern about mosquito-borne diseases

a. In relation to various issues that you and your family have to manage on a daily basis, how much are you concerned about diseases transmitted by mosquitoes, such as dengue, chikungunya, yellow fever and Zika?
b. Are these four diseases of equal concern to you, or is one of more concern than the other three? *Detail.*

7. Knowledge about Zika

a. Turning specifically to Zika, do you know anyone personally who has had Zika?
b. If so, what is your relationship with this person / people?
c. What do you know about Zika?
d. Are there any aspects of the disease you would like to know more about?

8. Sources of knowledge about Zika

a. Where did you receive your knowledge about Zika? *(Prompt to include social media)*
b. Which of the Zika information sources do you think was the best, and which have been the least useful?

9. Messages from Zika

a. What are the main messages about Zika that you received from the authorities? *Poll for mosquito control, bite reduction and pregnancy issues.*
b. Were these messages useful for you, or not? Explain. *Prompt for understanding, action, relevance, communication channel and key messages.*

10. Postponement of pregnancy

a. Do you know women in your community who wanted to postpone pregnancy as a means of avoiding a baby with microcephaly?
b. Has this issue been a matter of concern or discussion in your community?

11. Sexual transmission of Zika

a. The Zika virus can be sexually transmitted to women by infected men. Do you think that the men in your community would be willing to practice safe sex in the recommended six months if their partner was pregnant, and they knew they were infected with Zika? *Prompt for condom use, sex without penetration etc.*
b. Can you think of any messages that could be used to encourage men recently infected with Zika to practice safer sex? [MEN ONLY]
c. The Zika virus can be sexually transmitted to women by infected men. Would you be willing to use a condom for the recommended six months if your partner was pregnant and knew you were infected with Zika?

12. Abortion

a. Are you aware of cases in your community of pregnant women who have sought abortions because they feared they were carrying a baby with microcephaly?
b. If so, what did people say about it?
c. Do people in your community agree that a woman should have the right to terminate the pregnancy in these circumstances, or not? Or do they think she should carry the baby to term even if the baby may have microcephaly?

For peer review only

CLOSURE

- **Provide an opportunity for participants to discuss and ask questions about anything about Zika that they are in doubt about.**

- **Provide an official Zika information leaflet from the Ministry of Health website.**

- **Final question:** ***Would you be willing to attend a meeting to discuss the results of our study in 2 or 3 months? If so, please provide us with your contact details so that we can contact you.***

- **Thank all participants for their involvement and valuable responses.**

For peer review only

Supplementary File 2: Consolidated criteria for reporting qualitative research (COREQ): a 32-item checklist [1]

Bancroft *et al.* Vector control strategies in Brazil: A qualitative investigation into community knowledge, attitudes and perceptions following the 2015–16 Zika virus epidemic. *BMJ Open* 2021 [Manuscript ID: bmjopen-2021-050991]

**Domain 1:** Research team and reflexivity	Response		Referenced
**1.** **Interviewer/ facilitator**<br>Which author/s conducted the interview or focus group?	Yes	**Salvador lead:** Jorge Iriart<br>Facilitators: Vera Lucia Zaher-Rutherford, Tania Boccia, Mônica Manir.<br>**Jundiaí lead:** Eduardo Massad (Principal investigator)<br>Facilitators: Ana Maria Rico, Greice Bezerra Viana, Fernanda Macedo da Silva Lima.	p.6 (123-125)<br>p.20 (573-575)<br>p.20 (578)
**2.** **Credentials**<br>What were the researcher's credentials?	Yes	**Grace Power:** Project Manager at the Global Vector Hub, London School of Hygiene & Tropical Medicine (LSHTM), UK.<br>**Dani Bancroft:** MSc student, Department of Public Health, Environments and Society, Faculty of Public Health and Policy, LSHTM, UK.<br>**Robert Jones:** Research Fellow in Department of Disease Control, Faculty of Infectious and Tropical Diseases, LSHTM, UK.<br>**Jorge Iriat:** Associate Professor, Institute of Collective Health (ISC), Federal University of Bahia, Brazil.<br>**Eduardo Massad:** Professor, School of Medicine, University of São Paulo and Fundação Getulio Vargas, Brazil.<br>**Raman Preet:** Research Coordinator, Department of Epidemiology and Global Health, Faculty of Medicine, Umeå University, Sweden.<br>**John Kinsman:** Associate Professor, Department of Epidemiology and Global Health, Faculty of Medicine, Umeå University, Sweden.<br>**James Logan:** Head of Department of Disease Control, Faculty of Infectious and Tropical Diseases, LSHTM, UK.<br>**Interview facilitators:** local MDs, nurses, psychologists and sociologists.	All authors:<br>p.20 (578-582)
**3.** **Occupation**<br>What was their occupation at the time of the study?	Yes		Facilitators:<br>p.6 (111-112)<br>p.20 (573-575)

For peer review only

**4.**	**Gender**<br>Was the researcher male or female?	Yes	Both Salvador and Jundiaí interview teams consisted of one male coordinator and three female interview facilitators.	p.20 (573-575)
**5.**	**Experience and Training**<br>What experience or training did the researcher have?	Yes	The principal investigators in Salvador and Jundiaí are native Brazilian Portuguese speakers familiar with the local context of Zika virus in Bahia and São Paulo. The ZikaPLAN team carried out training and pilot testing of instrument with LSHTM visiting researchers. This was designed following 17 in-depth interviews with health professionals, including Salvador health professionals working in a Primary Care Unit and in private clinics, and community leaders, with three religious leaders from Kardecism, Candomblé (an Afro-Brazilian religion) and an evangelical Christian church.	p.6 (123-128)<br>p.20 (578-582)
**6.**	**Relationship**<br>Was a relationship established prior to study commencement?	No	No prior relationship was established.	N/A
**7.**	**Participant knowledge**<br>What did the participants know about the researcher? e.g. personal goals, reasons for doing the research	Yes	There were no direct benefits to participating in the study. Participants were provided information on the study objectives and relevance of the research, and a leaflet on Zika virus published by the Brazilian Ministry of Health at the end of the study.	N/A
**8.**	**Interviewer**<br>What characteristics were reported about the interviewer/facilitator?	Yes	The principal investigators in Salvador and Jundiaí are native Brazilian Portuguese speakers familiar with the local context of Zika virus in Bahia and São Paulo states. Interview facilitators were also local to the study sites.	p.6 (111-112)<br>p.6 (123-125)

**Domain 2:** Study design		Response		Referenced
**9.**	**Methodological orientation and Theory**<br>What methodological orientation was stated to underpin the study? e.g. content analysis.	Yes	Thematic analysis guided by Braun and Clarke (2006).[2]	p.6 (134)
**10.**	**Sampling**<br>How were participants selected?<br>e.g. purposive, convenience, consecutive.	Yes	Purposive sample for women of reproductive age (18–49). Not all men recruited into the study were the intimate partners of female participants.	p.6 (115-120)

For peer review only

**11.**	**Method of approach**<br>How were participants approached?<br>e.g. face-to-face, telephone, email.	No	Face-to-face recruitment at outpatient clinics, NGO settings and through researcher networks in the community.	p.6 (115-120)<br>p.6 (125-128)
**12.**	**Sample size**<br>How many participants were in the study?	Yes	A total of 120 participants: 103 women in focus groups (60 in Jundiaí and 43 in Salvador) and 17 men in semi-structured interviews (9 in Jundiaí and 8 in Salvador).	p.2 (18-19)<br>p.7 (141-145)<br>p.7 (147)
**13.**	**Non-participation**<br>How many people refused to participate or dropped out? Reasons?	No	The original study protocol proposed 6–8 women per focus group. Salvador groups ranged from 4–7. For Jundiaí, the size of number of participants in each focus group was not provided for data analysis.	p.7 (141-145)<br>[Table 1, p.7]
**14.**	**Setting of data collection**<br>Where was the data collected?<br>e.g. home, clinic, workplace	Yes	In Salvador, interviews were conducted in outpatient rooms at Primary Care Clinics (FGDs) and at private residences (for men). FGDs in Jundiaí were conducted at an NGO-run community centre and in University Hospital faculty buildings.	p.6 (117-120)
**15.**	**Presence of non-participants**<br>Was anyone else present besides the participants and researchers?	Yes	Visiting LSHTM researchers observed the interviews. No non-ZikaPLAN staff were present for the interviews.	p.6 (124-125)<br>p.20 (573-575)<br>p.20 (578)
**16.**	**Description of sample**<br>What are the important characteristics of the sample? e.g. demographic data, date	Yes	Interviews took place between March and August 2017. Sociodemographic data was not collected during all interviews; stratified age groups were provided for the majority of female FGDs but not for male participants.	p.6 (106-108)<br>p.6 (115-117)<br>p.7 (143-145)<br>p.18 (526-528)
**17.**	**Interview guide**<br>Were questions, prompts, guides provided by the authors? Was it pilot tested?	Yes	The topic guide, which includes questions, prompts and the sociodemographic data collected is provided in Supplementary File 1. This was pilot tested during training of interview facilitators with LSHTM research team present.	p.6 (108-111)<br>p.6 (124-125)<br>[Supplementary file 1]
**18.**	**Repeat interviews**<br>Were repeat interviews carried out? If yes, how many?	No	No follow up interviews were carried out, although all interview participants were invited to attend a follow-up session in September 2017 for dissemination of initial findings.	p.6 (128-130)<br>[Supplementary file 1]
**19.**	**Audiovisual**<br>Did the research use audio or visual recording to collect the data?	Yes	The source data was audio recordings that were transcribed into Brazilian Portuguese by the Brazil ZikaPLAN team. This was then translated into English, with excerpts of transcripts verified for accuracy and credibility by the University College London Digital Media translation service. The source data was not shared for data analysis.	p.6 (111-112)<br>p.18 (523-526)<br>p.20 (578-580)

For peer review only

**20.**	**Field notes** Were field notes made during and/or after the interview or focus group?	Yes	ZikaPLAN observers and facilitators took field notes during the sessions.	N/A
**21.**	**Duration** What was the duration of the interviews or focus group?	Yes	Each interview was arranged to last 60–90 minutes. Timestamps for interviews were not shared for analysis, but the wordcount of each transcript was presented in Table 1.	p.7 (142-145) [Table 1, p.7]
**22.**	**Data saturation** Was data saturation discussed?	Yes	Yes, regarding participant responses to question 5 in the topic guide on novel repellents for personal protection.	p.19 (539-541) [Supplementary file 1].
**23.**	**Member checking** Were transcripts returned to participants for comment and/or correction?	No	No, although all interview participants were invited to attend a follow-up session in September 2017 for dissemination of initial findings.	p.6 (128-130) [Supplementary file 1]

**Domain 3:** Analysis and findings		Response		Referenced
**24.**	**Coders** How many data coders coded the data?	Yes	One researcher for initial coding and three authors of one full FGD transcript. The principal investigators in Brazil carried out an initial analysis of transcripts following data collection. The data was then passed on to LSHTM for independent data analysis. The initial coding framework was presented to the principal investigators in Brazil for confirmability and triangulation purposes prior to theme generation.	p.6 (128-130) p.6 (134-136) p.18 (523-526)
**25**	**Coding tree** Did authors provide a description of the coding tree?	Yes	The full codebook is provided in Supplementary File 3. A summary table of the key and major themes and a concept map of minor themes are provided in the manuscript.	p.7 (152-154) [Table 2, p.8] [Supplementary file 3]
**26**	**Derivation of themes** Were themes identified in advance or derived from the data?	Yes	Coding was derived from the data. Theme generation was mostly inductive, with some deductive elements from grouping of codes together as responses to a certain question in the topic guide. Major themes were later mapped against constructs in a pre-defined conceptual framework for behaviour change for a potential fit (Rosenstock's Health Belief Model).	p.5 (89-92) p.6 (133-138) p.7 (148-154) [Figure 1; Figure 2] p.18 (528-532)
**27.**	**Software** What software, if applicable, was used to manage the data?	Yes	Microsoft Excel was used to record sociodemographic data for each interview and observations, as well as administrative data, such as wordcount, date and file names for the Brazilian and English transcripts as an audit trail. NVivo 12 (QSR International, 2012) was used for coding and mapping Figure 2. Figure 2 was later redesigned in Lucidchart (Lucid Software Inc., 2021).	p.6 (133-134) [Figure 1; Figure 2]

For peer review only

**28.**	**Participant checking** Did participants provide feedback on the findings?	No	At the end of each interview participants were invited to consent for their contact information to be collected to disseminate the research findings. However, participant checking was not possible for this study.	p.6 (128-130) [Supplementary file 1]
**29.**	**Quotations presented** Were participant quotations presented to illustrate the findings? Was each quotation identified?	Yes	Quotations in the manuscript were identified by focus group or interview site and number (unit of analysis), with the corresponding age group (18–30 or 31–49) in Table 1.	[Results section] p/6 (106-107) p.7 (141-145) [Table 1, p.7]
**30.**	**Consistency** Was there consistency between the data presented and the findings?	Yes	–	p.18 (528-533) p.19 (559-560)
**31.**	**Clarity of major themes** Were major themes clearly presented in the findings?	Yes	A concept maps for themes was produced and this was used to navigate description of findings in relation to one another.	p.6 (137-138) p.7 (152-154) [Figure 2; Table 2, p.8]
**32.**	**Clarity of minor themes** Is there a description of diverse cases or discussion of minor themes?	Yes	Key and major themes are defined in Table 2 in the manuscript, and minor themes described in the findings. All themes are defined fully in the codebook (Supplementary File 3).	p.7 (148-154) [Table 2, p.8] [Results section] [Supplementary file 3]

1 Tong A, Sainsbury P, Craig J. Consolidated criteria for reporting qualitative research (COREQ): a 32-item checklist for interviews and focus groups. *International Journal for Quality in Health Care* 2007;19(6):349–357. doi:10.1093/intqhc/mzm042

2 Braun, V, Clarke V. Using thematic analysis in psychology. *Qual Res Psychol* 2006;3:77–101. doi:10.1191/1478088706qp063oa

For peer review only

Supplementary File 3: Final transcript coding framework (Codebook)

Bancroft *et al.* Vector control strategies in Brazil: A qualitative investigation into community knowledge, attitudes and perceptions following the 2015–16 Zika virus epidemic. *BMJ Open* 2021 [Manuscript ID: bmjopen-2021-050991]

**1. KNOWLEDGE**	Knowledge of MBD and ZIKV at the time of the study, and cues to action which are recalled stimuli for a decision-making process that may lead to behaviour change.[32]
**1.1 Knowledge of MBDs**	Depth of understanding of ZIKV/MBDs, vector control and misinformation.
Key messages	Reponses to Question 9 in the topic guide: "What are the main messages about Zika that you received from the authorities?" (Poll for mosquito control, bite reduction and changes in behaviour for reproductive health).
MBD outbreaks	General knowledge on other mosquito-borne diseases: yellow fever, chikungunya, dengue fever. For example, references to outbreaks and epidemics, changes in prevalence/incidence, pathophysiology and vaccination campaigns.<br>Excluded: comments where ZIKV is the focus (coded as 'ZIKV General'), unless being compared to other MBDs.
Misinformed	Comments made by participants that may indicate misinformation or uncertainty around key messages related to MBDs.
Sexual transmission	Knowledge related to sexual transmission of ZIKV of both the participant and others in their social circle.<br>Excluded: content of messaging related to sexual transmission (coded as 'Key messages').
ZIKV (General)	Other knowledge related to ZIKV that does not fall into codes sexual transmission, severity of ZIKV symptoms, perceived risk (susceptibility), or experience of ZIKV (internal cues to action).
**1.2 External cues to action**	Stimuli from members of participants social network, the media, healthcare providers, the workplace or other community groups that trigger a decision-making process to seek additional information, engage in vector control or mosquito-bite reduction strategies, or other health seeking behaviours.
Health campaign	Alerts, visits from health agents for risk communication, billboards, posters and pamphlets, or messaging in the media explicitly described by the participant as being official public health information.
Zika Projects	The *Zika Project*, official NGO or volunteer projects taking place in hospitals (not always clear).<br>Excluded: activities identified as being conducted by local or national authorities (e.g. City Hall, Ministry of Health).
Healthcare	Accessing different forms of healthcare, such as maternity services, community clinics, dentists etc.<br>Excluded: experiences of having ZIKV or other MBDs, descriptions of symptoms of poor health (coded 'Other poor health').

For peer review only - http://bmjopen.bmj.com/site/about/guidelines.xhtml

Media	Parent code for references to media. Excluded: Official health campaign content (when clearly identified).
Broadcast media	Any media source that has been broadcast for entertainment purposes, such as television soaps and radio, or TV advertisements and print media, such as magazines and newspapers.
Online and social media	Casual or purposeful research online: accessing websites that may provide information about ZIKV. Messages and advertisements through social media, such as WhatsApp, Facebook, Instagram etc.
Social circle	Friends, neighbours, family members. Excluded: co-workers or acquaintances in formal settings (e.g. university, volunteer groups)
Work or education	Parent code for references to formal settings. Excluded: volunteering positions (e.g. in hospitals or ZIKV projects).
Higher Education	Participant is a current or former university/college student where ZIKV messaging has been delivered as part of a formal curriculum. Or there have been opportunities to access lectures and seminars on the epidemic.
Schools	Recalling experience of formal education for participants (e.g. high school). Or messages that children in the participants social circle have passed on to the participant informally.
Workplace	Participant either works in healthcare, formal education (teachers) or other profession where Zika messaging has been delivered at their workplace (e.g. works for the City Hall).
Community groups	Observing preventive activities or other stimuli in the community: informal groups (e.g. women's groups, gangs), community volunteer groups, gangs, centres of worship, neighbourhood associations, sports teams (e.g. capoeira, football) etc.
Government	National, state and municipal levels of government responsible for defining activities and protocols for *Aedes* interventions, including *"budget, personnel, technical guidelines, approved substances, routines, evaluation, and relationships with other sectors, such as education and public health"*.[4]
Local authorities	Aldermen, City Hall urban planning including waste management services. Health agents from the City Hall. Excluded: 'health agents' described as being from an NGO, Ministry of Health or other national body.
National authorities	References to the national government: politicians, deployment of the army, legislation and policy makers, the Ministry of Health (e.g. official surveillance staff from the Brazilian MoPH) or other national bodies.
No action	No vector control strategies are recalled to have taken place in the community, except for examples of vector control activities that have taken place more than one year prior to the start of epidemic in 2015.

For peer review only - http://bmjopen.bmj.com/site/about/guidelines.xhtml

**1.2 Internal cues to action**	Personal or secondary experience of confirmed/suspected cases of MBDs provide a stimulus for a decision-making process that leads to health seeking behaviour.
Experience of other MBDs	Confirmed or suspected cases of non-ZIKV mosquito-borne arboviruses by the participant or in the participant's social network.
Experience of Zika	Confirmed or suspected cases of ZIKV infection of the participant or in the participants social network.
Other poor health	Discussion of poor health that might be: non-communicable (e.g. disability or chronic conditions); related to non-ZIKV pregnancy complications; infectious diseases such as measles and H1N1 viruses; and other vector borne diseases such as Leptospirosis, tick-borne diseases, Chaga's disease etc. Excluded: MBDs.

**3. ATTITUDES & NORMATIVE BELIEFS**	Personal attitudes are internal assessments of knowledge and cues to action for MBD preventive behaviours. Normative beliefs may inform personal attitudes according to how others perceive the behaviour in a social setting, such as the community.[1]
**3.1 Perceived Susceptibility**	A subjective assessment of risk of ZIKV infection or a CZS pregnancy. Combines with perceived severity for perceived threat.[2]
Mosquito population	Comments on the burden of the mosquito population in a specific geographical area, mosquito physiology and behaviour. Other observations made by the participant or members of the participants social circle on the activity of mosquitoes in that area.
Risk response	Perceived risk of ZIKV transmission and CZS. For example: the periodomicile does not have a large mosquito population; the participant is not pregnant or has undergone the menopause; perceptions that the risk of contracting ZIKV to be very low. (Also includes responses to question 5 of the topic guide).
**3.2 Perceived Severity**	A subjective assessment of the severity of ZIKV and potential consequences of infection or a CZS pregnancy. "The combination of perceived severity and perceived susceptibility is referred to as perceived threat".[2]
CZS severity	Experience of caring for a child with microcephaly in the in the participants social network.<br>Perceptions of the severity of microcephaly in the community, e.g. the burden of care giving for a child with microcephaly (the financial or social implications). Excluded: comments around male support to care for a child with CZS.
ZIKV Severity	Perceptions related to the severity of symptoms of ZIKV. Comments about concern or even fear related to ZIKV. Excluded: comments about CZS caregiving.
Other MBD Severity	Perceptions related to the severity of symptoms of other MBDs. Comments about concern or fear related to other MBDs. Excluded: Perceptions of poor health due to non mosquito-borne arboviruses.

For peer review only

For peer review only - http://bmjopen.bmj.com/site/about/guidelines.xhtml

**3.3 Perceived Barriers**	An individual's assessment of the obstacles to ZIKV preventive behaviours, including condom use to prevent sexual transmission, mosquito bite-reduction and vector control strategies.
Abortion	Awareness of individuals in the community that have terminated a pregnancy due to ZIKV or has undergone an abortion themselves as a result of concern of giving carrying a microcephaly child. Also includes community perspectives on the acceptability of abortion. Excluded: rights to abort and legislation.
Abortion rights	Participant responses to Question 12 in the topic guide: "Do people in your community agree that a woman should have the right to terminate pregnancy in these circumstances? Or do you understand that she should carry the pregnancy through to the end even if the baby has microcephaly?"
Depends on circumstances	More consideration around abortion. Comments that it is both acceptable and unacceptable, with examples of scenarios where abortion may be necessary or comments such as 'it's difficult' or 'it's complicated'. Includes discussion of financial circumstances and male partner support to evidence reasoning (only in reference to abortion). Excluded: caring for a child with CZS.
Opposed to abortion	Explicit opposition to the rights to abort. May cite religious grounds and morality e.g. perceptions of foetal viability and human rights. Normative beliefs around responsibility of pregnant mothers and their male partners. Unspecified negative responses, or strong opposed even when prompted by thee interviewer about microcephaly.
Supports rights to abort	Explicit support for the right to choose abortion. May express the need for legislative change, or cite perceptions of women's rights and autonomy regarding reproductive health.
Unclear response to abortion	Conflicted, contradictory or unintelligible response. May indicate discomfort expressing personal attitudes that conflict with the majority position.
Repellent acceptance	Parent code for likelihood of community acceptance of novel repellents adoption (response to question 5 of the topic guide).
Appearance response	Aesthetic criteria related to the perception of wearing novel novel repellents in the community (e.g. smell, fashion).
Comfort response	Negative responses related to comfort of repellent clothing such as overheating, restricting physical movement and allergies or discomfort caused by repellent products.
Repellent effectiveness	Responses related to perceived effectiveness of novel repellents for mosquito bite reduction. Scepticism or expression of interest may be contingent on how effective novel repellents are in practice (response to question 5 of the topic guide).

For peer review only

Repellent accessibility	Parent code for perceptions of the ability to access novel novel repellents (response to question 5 of the topic guide).
Affordability response	Comments related to cost of novel novel repellents being a barrier to their adoption.
Availability response	Comments related to local availability of repellent tools for purchase, such references to vendor stock outs and likelihood of vendors in their community to sell novel repellent tools like clothing. Also included are comments around provision of novel repellents as gifts-in-kind from NGOs or the local or national authorities (e.g. through Bolsa Familia).
Awareness response	Participants awareness of novel repellent tools for personal protection. Comments about being unaware or vague.
Community cohesion	Social cohesion is defined as the *"extent of connectedness and solidarity among groups within society"*,[3] such as support from the community for vector control or being able to seek social support when unwell. Comments about absent or poor relationships with neighbours, or not allowing unsolicited calls to household due to concerns about neighbourhood violence
Responsibility	Observation about participants expressing frustration over current preventive practices or ZIKV messaging, or being unable to negotiate shared responsibility for communal spaces for vector control. Blame of third parties or authorities.
Internal responsibility	Expressing perceived locus of control for behaviour change lies with individual.
External responsibility	Expressing that the perceived locus of control in relation to behaviour change around ZIKV and messaging as lying further upstream, such as with authorities (local, national).
Male support	Perceptions of male partners and the level of support participants feel they have from partners for ZIKV prevention. Perceptions of other male members of participants social circles, including family members, including normative beliefs related to gender (e.g. machísimo). Excluded: references to condom negotiation.
Negotiating condom use	Responses to question 11 of topic guide: "Do you think that the men in your community would be willing to practice safe sex (condom use, sex without penetration)? Do you think that if a man knew he was infected he would use a condom for six months?"

For peer review only - http://bmjopen.bmj.com/site/about/guidelines.xhtml

**3.4 Perceived benefits and self- efficacy**	"Perceived benefits refer to an individual's assessment of the value or efficacy of engaging in a health-promoting behaviour to decrease risk of disease."[2] Self-efficacy refers to an individual's perception of their competence to successfully undergo a behaviour change.[2]
Comfort	Positive perceptions of novel repellents use such as avoiding discomfort from bed nets, overheating from having to close windows and doors at night time, 'stickiness' or dislike of wearing topical repellents and allergic reactions (if referring to clothing).
Protection	Responses to question 5 of the topic guide related to enhanced protection of themselves or others in their social network from MBD infection. e.g. during pregnancy, family members such as children or the elderly.
Likelihood of adoption	Willingness or likelihood to adopt novel repellents. Describes being motivated or unmotivated to take responsibility for household level behaviours or community participation to reduce transmission of ZIKV. Excluded: change in behaviour that has happened.
Negative response	Unspecified negative response to Q5 of the topic guide indicating disinterest or not willing to adopt novel repellents.
Positive response	Unspecified positive response to question 5 of the topic guide indicating willingness or interest to adopt novel repellents.

**3. BEHAVIOUR CHANGE**	Behaviour changes attributed to the ZIKV epidemic, existing MBD preventive behaviours or no changes to mosquito population control or bite-reduction strategies, including use of novel repellent tools.
**3.1 Household Level**	Practices to prevent mosquito breeding sites, mosquito-bite reduction and mosquito entry to the household.
Mosquito bites	Preventive practices taken personally to reduce risk of mosquito bites.
Avoidance behaviour	Avoiding certain times of day or areas known to have more mosquitoes. Closing of windows or doors to prevent mosquito entry.
Bed nets, screens	Insecticide treated or untreated mosquito bed nets, window or door screens to prevent mosquito entry.
Electronic devices	Plug in mosquito repellent devices, air conditioning and fans, electric 'racket' killing devices, sonic devices.
Long clothing	Covering up with long sleeves or legs to prevent exposed skin to mosquitoes.

For peer review only - http://bmjopen.bmj.com/site/about/guidelines.xhtml

Other topical products	Applying moisturiser, sun screen or other topical lotions that are not manufactured to function as mosquito repellents.
Repellents	Chemical or citronella repellents, room sprays or alternative methods like burning coils, egg shells, cardboard etc. Excluded: electronic plug-in repellents or sonic devices.
Supplements	Participants describe taking oral supplements due to belief this will reduce likelihood of mosquito bites (e.g. vitamin B complex).
Mosquito population control	Parent code for preventive practices related to vector control in the household.
Animals	Wild dogs, pets or other non-arthropod animals. Coded for potential implications for One Health.
Garbage disposal	Further detail relating to garbage collection or recycling to prevent water accumulation.
Hygiene	Using soap, scrubbing surfaces, applying disinfectant, sweeping and references to hygiene and cleanliness.
Insecticide	Water treatments to stop larval growth cycle (larvicides), or spraying chemical insecticides indoors or around the periodomicile.
Stagnant water	Practices to prevent pooling of water in the periodomicile: filling plant pots or receptacles with sand; removing rubble; turning over pots and drinks bottles; wiping condensation down from surfaces, or other measures to encourage drainage and prevent stagnancy.
Behaviour adoption	Behaviour change attributed to ZIKV; including comments on increased or decreased frequency of an activity.
Delaying pregnancy	Decision to prevent or delay pregnancy, detailing methods that include use of contraceptives, non-penetrative sex, abstinence etc. Also referrals to members of the social circle or their wider network that delayed pregnancy. Excluded: abortion.
No change	Behaviours were practiced before ZIKV epidemic, or no adoption of preventive practices since the ZIKV epidemic.
**3.2 Community Participation**	Participant has engaged with others in the community, describing activities for collective action for vector control since the arrival of the ZIKV epidemic.
Collective Action	Engaging with others for activities specific to vector control, e.g. consulting with neighbours or community groups, exchanging advice with members of their immediate social circle.

For peer review only

Reporting	Reporting of sources of concern for mosquito control (e.g. communal spaces and garbage, larval growth) to landlords or building maintenance staff, local authorities, health agents or other third parties in position of power.

**4. COMMUNITY PREFERENCES**	Expressed needs or elaboration of preferences for mosquito-abatement products, or coordination of vector control strategies and health promotion related to ZIKV.
**4.1 Personal protection**	Novel topical mosquito repellents, repellent-impregnated clothing or other wearables (e.g. plastics) designed to repel and prevent mosquito bites.
Preferred criteria	Preferred criteria for novel repellents and repellent wearables that would encourage adoption, such as responses relating to comfort, appearance, affordability, effectiveness and other responses to question 5 of the topic guide.
Suggestions	Responses where participant mention a criterion for novel repellents not coded for in the other responses, e.g. suggestions for alternative repellent products (e.g. microencapsulated bracelets). Any other responses to question 5 of the topic guide.
**4.2 ZIKV Messaging**	Preferred risk communication and community engagement for MBD surveillance, mosquito bite-reduction and vector control strategies. Responses to: “Which of the Zika information sources do you think was the best and which was the least useful?”
Preferred delivery	Preferred format, frequency and source of delivery of risk communication (e.g. social media, in person).
Preferred target audience and messaging	Preferred target for risk communication and community engagement where participants express there is the most need (e.g. men, school children) and preferred key messages or specific topics related to ZIKV and MBDs.
Questions	Expressing lack of understanding or requests for clarification on topics related to ZIKV or other MBDs.
**4.3 Vector control**	Preferred activities for mosquito population control; perceptions of where the responsibility lies for vector control.
Community Level	Suggestions for action related to community groups, local authorities or within their local social network. e.g. health inspections or appointment of community members for capacity building and mobilisation of funding.
National Level	Preferred activities at the national level. For example, suggestions for action related to government policy and legislation, funding, public health campaigns or vaccine research and development.

For peer review only - http://bmjopen.bmj.com/site/about/guidelines.xhtml

**Discard pile**	Participant responses do not answer the topic guide questions or are considered relevant to the research question to justify creation of a new code.

1 Cislaghi B, Heise L. Theory and practice of social norms interventions: Eight common pitfalls. *Global Health* 2018;14(1):1–10.

2 Champion VL, Skinner CS. The Health Belief Model. In: Glanz, K., Rimer, B.K. & Viswanath, K., eds. Health Behavior and Health Education: Theory, Research, and Practice. San Francisco: Jossey-Bass 2008:45–66

3 Manca AR. Social Cohesion. In: *Encyclopedia of Quality of Life and Well-Being Research* Dordrecht: Springer Netherlands; 2014 [cited 2020 Sep 23]. p. 6026–8.

4 Carvalho MS, Honorio NA, Garcia LMT, *et al.* Aedes ægypti control in urban areas: A systemic approach to a complex dynamic. *PLoS Negl Trop Dis* 2017;11(7):1–15.

For peer review only

For peer review only - http://bmjopen.bmj.com/site/about/guidelines.xhtml
